# Ultra-Sensitive All-Polymer Near-Infrared Photodetectors via Van der Waals Layered Triple Heterojunction

**DOI:** 10.34133/research.0939

**Published:** 2025-10-03

**Authors:** Lei Guo, Meiyu He, Jiayue Han, Xingwei Han, Chao Han, Lixin Liu, Xiutao Yang, He Yu, Jun Gou, Jun Wang

**Affiliations:** ^1^ School of Optoelectronic Science and Engineering, University of Electronic Science and Technology of China, Chengdu 610054, P. R. China.; ^2^ State Key Laboratory of Electronic Thin Films and Integrated Devices, University of Electronic Science and Technology of China, Chengdu 610054, P. R. China.

## Abstract

All-polymer near-infrared (NIR) organic photodetectors (OPDs) offer exceptional stability and stretchability, making them highly promising for next-generation wearable electronics, biomedical sensing, and imaging applications. However, their practical implementation remains hindered by limitations such as low responsivity, high noise, and limited sensitivity, highlighting the critical challenge of optimizing the active layer structure to enhance device performance. In this study, we propose a van der Waals layered triple heterojunction (LTHJ) structure fabricated via water transfer printing (WTP) to reduce trap density, improve interfacial quality, optimize charge transport pathways, and enhance carrier dissociation and extraction efficiency. The LTHJ OPD exhibits simultaneously low noise and high responsivity, achieving ultra-low dark current (0.38 pA at −0.1 V; 2 pA at −2 V), an ultra-high switching ratio (>10^9^), and a specific detectivity exceeding 10^14^ Jones. To the best of our knowledge, this performance represents one of the best-reported all-polymer OPDs to date, providing a novel strategy for developing high-performance polymer-based photodetectors. Furthermore, we demonstrate the potential of LTHJ OPDs in optical integrated sensing and communications (O-ISAC) by achieving obstacle-penetrating optical wireless communication (OWC) and long-distance misalignment photoplethysmography (PPG) signal monitoring, further underscoring their applicability in next-generation intelligent sensing and complex communication environments.

## Introduction

Near-infrared (NIR) photodetectors have extensive scientific and industrial applications in environmental monitoring, artificial vision, medical imaging, security systems, and optical communications, leading to a rapidly growing demand [1–3]. However, current NIR photodetectors primarily rely on silicon or inorganic compound semiconductors such as InGaAs and HgCdTe [4,5], which require high-purity materials and complex fabrication processes, resulting in high production costs. Organic photodetectors (OPDs) have garnered substantial interest due to their unique advantages, including flexibility, wearability, solution processability, and tunable spectral response, making them promising alternatives to silicon-based photodiodes [6–10]. Among OPDs, all-polymer photodetectors, composed of π-conjugated polymer donors and polymer acceptors, exhibit superior mechanical stretchability, excellent morphological stability, and compatibility with industrial manufacturing. These advantages position all-polymer photodetectors as promising candidates for commercialization in portable health monitoring, optical imaging, and optical communication [11,12]. However, the performance of all-polymer optoelectronic devices, such as photodetectors and solar cells, still lags behind that of small-molecule acceptor- and perovskite-based devices [13–16]. For instance, perovskite solar cells have achieved a power conversion efficiency of 29.1% [17], and small-molecule acceptor-based organic solar cells have widely surpassed 20% [18–21], whereas all-polymer solar cells have yet to reach 20% [22,23]. The limited availability of polymer acceptors and challenges in controlling the morphology of all-polymer blends further hinder the performance of all-polymer OPDs, which often suffer from high dark current. This issue is primarily attributed to increased thermal emission, high trap-state density, pronounced charge recombination, and restricted charge transport, posing substantial challenges for further development [24,25].

To further enhance the sensitivity of all-polymer NIR OPDs, it is crucial to optimize interfacial engineering and device architecture within the all-polymer blend system to reduce energetic disorder and trap states. Although this process is challenging, it can substantially improve the device’s detection performance. Active layer modulation is an effective strategy for enhancing OPD performance. Traditional OPDs primarily employ bulk heterojunction (BHJ) or planar heterojunction (PHJ) structures [26,27], but these architectures present a trade-off between achieving low dark current and high responsivity, making optimization difficult [28,29]. In recent years, the pseudo-planar heterojunction (PPHJ) structure has emerged as one of the most advanced optimization strategies in organic solar cells [30], achieving remarkable progress and demonstrating substantial advantages. The PPHJ structure effectively facilitates exciton dissociation, charge generation, and extraction while also outperforming conventional BHJ devices in terms of power conversion efficiency, stability, transparency, and flexibility [31,32]. With continued research, the PPHJ structure has gradually been introduced into OPDs [33]. Organic thin films fabricated via the layer-by-layer process exhibit vertical phase separation, which promotes carrier generation and transport while effectively suppressing charge recombination. This leads to reduced dark current and enhanced device sensitivity. Additionally, approaches such as annealing process optimization and material modification have also been reported to achieve vertical phase separation, further reducing dark current [34–36]. Maintaining a high optical response while reducing the dark current by optimizing the structure of the active layer is an important research direction to improve the sensitivity of OPDs, which is highly valuable and challenging to research.

In this study, we constructed a van der Waals (vdW) multilayer structure layered triple heterojunction (LTHJ) using a water transfer printing (WTP) process. The physical lamination effectively mitigates trap-induced chemical disorder within the BHJ layer while simultaneously enhancing the carrier injection barrier, thereby suppressing the dark current increase caused by disordered carrier injection and saturated generation current. Moreover, the LTHJ structure improves interfacial quality, minimizes the formation of interfacial trap states, and reduces trap-assisted recombination at interfaces, enabling an ultra-low dark current below the pA level. In addition, the LTHJ architecture enhances carrier transport efficiency, suppresses undesirable nonradiative recombination, and facilitates efficient exciton dissociation. As a result of these optimizations, our LTHJ OPD achieved an on/off ratio exceeding 10^9^, a detectivity surpassing 10^14^ Jones, and a linear dynamic range (LDR) of 192.87 dB. Moreover, we leveraged the high sensitivity of the LTHJ OPD to develop an optical integrated sensing and communication (O-ISAC) system and explored its applications in long-distance misalignment photoplethysmography (PPG) monitoring and optical wireless communication (OWC) through obstacles. This study presents a novel strategy for constructing high-performance all-polymer NIR OPDs and lays a solid foundation for the development of flexible and wearable devices based on highly sensitive all-polymer semiconductors.

## Results and Discussion

### Design and fabrication of the LTHJ OPD

**Fig. 1.**
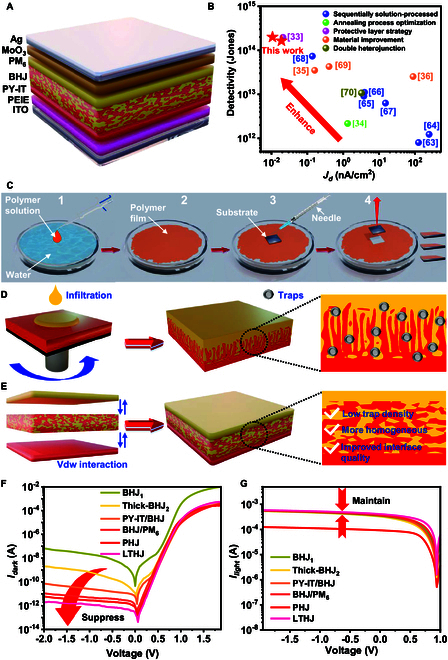
Device structure, fabrication process, and *I–V* characteristic curves. (A) Schematic diagram of the LTHJ OPD. (B) Performance comparison of D_sh_^*^ and *I*_dark_ between OPDs with a vertical phase-separated active layer structure (Table S2) and the LTHJ OPD. (C) Step-by-step schematic of the WTP process used for device fabrication. (D) Illustration of the PM_6_/PY-IT PPHJ. (E) Illustration of the LTHJ. (F) *I*_dark_ of different devices. (G) *I*_light_ of different devices under 850-nm light illumination.

The LTHJ OPD was designed to overcome the limitations of conventional BHJ or PHJ photodetectors by introducing an LTHJ with controlled vertical phase separation, improving charge transport while suppressing dark current. This study primarily employs 4 organic materials (Fig. S1). PM_6_ (poly[(2,6-(4,8-bis(5-(2-ethylhexyl)-4-fluorothiophen-2-yl)-benzo[1,2-b:4,5-b′]dithiophene))-alt-(5,5-(1′,3′-di-2-thienyl-5′,7′-bis(2-ethylhexyl)benzo[1′,2′-c:4′,5′-c′]dithiophene-4,8-dione))]) and PY-IT (poly[[12,13-bis(2-octyldodecyl)-12,13-dihydro-3,9-diundecylbisthieno[2″,3″:4′,5′]thieno[2′,3′:4,5]pyrrolo[3,2-e:2′,3′-g][2,1,3]benzothiadiazole-2,10-diyl]methylidyne[1-(dicyanomethylene)-1,3-dihydro-3-oxo-2H-inden-yl-2-ylidene]-2,5-thiophenediyl[1-(dicyanomethylene)-1,3-dihydro-3-oxo-2H-inden-yl-2-ylidene]methylidyne]) are explored in detail as examples, and their spectral absorption ranges are shown in Fig. S2. The definitions of the photodetector-related terms discussed in this study are provided in Table S1. As illustrated in Fig. 1A, the device architecture comprises an indium tin oxide (ITO)/polyethylenimine (PEIE)/PY-IT/PM_6_:PY-IT/PM_6_/MoO_3_/Ag structure. The active layer adopts an LTHJ structure consisting of PY-IT, PM_6_:PY-IT, and PM_6_. The PY-IT layer was fabricated via spin-coating, whereas the PM_6_:PY-IT and PM_6_ layers were deposited using the WTP technique (Text S1 and Materials and Methods). To characterize the multilayer structure of the LTHJ, x-ray photoelectron spectroscopy (XPS) analysis was performed. The distinct variations in fluorine (F) content from PM_6_ (C_68_H_76_F_2_O_2_S_8_) and nitrogen (N) content from PY-IT (C_110_H_138_N_8_O_2_S_6_) at different etching times (0, 450, and 900 s) confirm the well-defined layered structure, as shown in Fig. S3. Compared to other vertical phase separation strategies (Fig. 1B and Table S2), LTHJ OPD achieves lower dark current and higher sensitivity. The detailed water transfer process is illustrated in Fig. 1C and further elaborated in Text S1 and Fig. S4.

Traditional OPDs use BHJ or PHJ structures (Fig. S5). PHJ OPDs have low dark current but poor responsivity, while BHJ OPDs improve responsivity but increase dark current due to donor–acceptor mixing. To maintain responsivity while suppressing dark current, the PPHJ structure has emerged as a promising alternative in recent years. PPHJ structures combine high responsivity with low dark current by leveraging the layer-by-layer fabrication technique, in which the upper-layer material partially infiltrates the lower-layer material. This process optimizes carrier transport pathways and increases donor–acceptor interfaces (Fig. 1D). However, the infiltration-based fabrication approach presents several challenges: (a) nonuniform infiltration, (b) formation of trap states, and (c) uncontrolled phase separation. Unlike PPHJ, the LTHJ structure proposed in this work leverages vdW interactions to achieve a vertically phase-separated architecture (Fig. 1E). This 3-layer sequential fabrication approach offers superior phase separation controllability, preventing mutual interference between layers, eliminating additional trap states, and avoiding inhomogeneous mixing in the BHJ layer. As a result, it achieves lower trap density and higher interfacial quality, effectively combining the advantages of both BHJ and PHJ structures.

To investigate the impact of multilayer organic thin films on device performance, we fabricated various OPDs with different active layer structures (Fig. S6) and conducted a comparative analysis of their optoelectronic properties. Figure 1F and G compares *I*_dark_ and *I*_light_ across device architectures, showing that BHJ OPDs have high photocurrent but also high *I*_dark_, while PHJ OPDs exhibit lower dark current but reduced photocurrent. Adding a donor (PM_6_) or acceptor (PY-IT) layer to the BHJ structure substantially reduces the dark current without affecting the photocurrent. Further investigation reveals that incorporating both the PM_6_ layer and the PY-IT layer on either side of the BHJ structure further suppresses the dark current, reducing it to the pA range, with an ultra-low dark current of just 0.38 pA at −0.1-V bias. Additionally, the photocurrent remains unaffected and is even slightly enhanced, reaching approximately 1.08 times that of the BHJ OPD at −2-V bias. Overall, the LTHJ OPD further enhances the “responsivity-to-noise” performance, enabling NIR OPDs with higher on/off ratios and improved sensitivity.

### Mechanism of dark current suppression

**Fig. 2.**
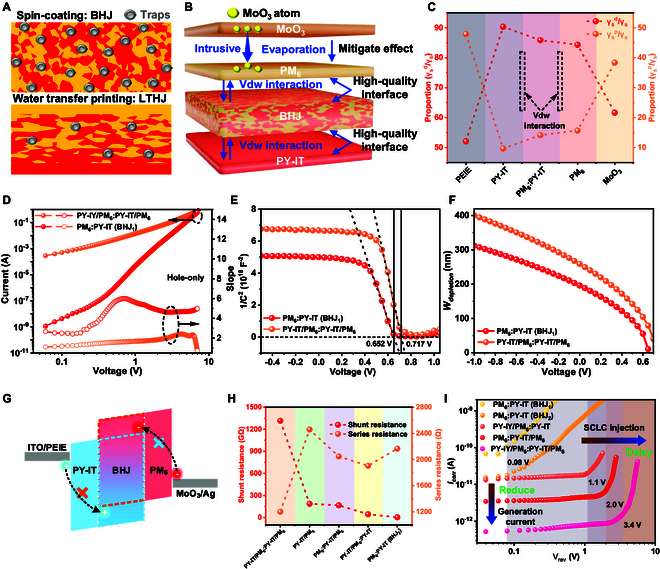
Dark current suppression. (A) Reduced trap density in LTHJ via WTP compared to BHJ. (B) Formation of high-quality interfaces in LTHJ. (C) 𝛾_s_^𝑑^/𝛾_s_ and 𝛾_s_^𝑝^/𝛾_s_ for different layers, including PEIE, PY-IT, BHJ, PM_6_, and MoO_3_. (D) *I*–*V* characteristics of hole-only devices with BHJ and LTHJ. (E) Mott–Schottky plots of BHJ and LTHJ devices at 1 kHz, with dashed lines indicating linear fitting results. (F) Depletion width (*W*_depletion_)–*V* curves of BHJ and LTHJ devices at 1 kHz. (G) New barriers are introduced through the PM_6_ layer and PY-IT layer. (H) Comparison of *R*_s_ and *R*_sh_ for different devices. (I) *I*_corr_–*V* characteristics.

In contrast to conventional spin-coating or blade-coating methods, the WTP technique enables the sequential fabrication of multilayer organic thin films without causing mutual interference while still maintaining high-quality active layers. This advantage arises from the use of vdW interactions, which, unlike covalent bonding, do not create dangling bonds at the interfaces [37,38]. Compared to spin-coated BHJ, LTHJ fabricated via WTP exhibits substantially lower trap density (Fig. 2A) and forms high-quality vdW interfaces (Fig. 2B), thereby effectively suppressing the dark current. Additionally, while MoO_3_ is an excellent hole transport layer, its thermal evaporation deposition method may cause atomic diffusion into the active layer, leading to defects [39,40]. In BHJ OPDs, MoO_3_ diffusion into the mixed donor–acceptor layer can create extra traps, disrupting morphology and charge transport. These traps capture carriers, increase thermally activated currents, and raise the dark current. In contrast, PHJ structures deposit MoO_3_ on a pure donor layer with better stability, resulting in fewer traps and more stable charge transport [41]. Therefore, the LTHJ structure improves both the internal active layer interfaces and the interface with MoO_3_. To accurately characterize the interlayer contact properties, the 2-liquid method was employed to measure the surface energy (𝛾_s_) of each layer (Text S2 and Fig. S7).

Surface energy can be decomposed into a dispersion component (𝛾_s_^𝑑^), which reflects vdW forces, and a polar component (𝛾_s_^𝑝^), which arises from short-range intermolecular interactions [42]. A high and similar ratio of the 𝛾_s_^d^/𝛾_s_ between layers indicates strong vdW interactions. As shown in Table S3 and Fig. 2C, PY-IT, PM_6_:PY-IT, and PM_6_ layers all exhibit a high 𝛾_s_^𝑑^, confirming the presence of robust vdW contacts between these layers. According to the one-dimensional drift-diffusion model proposed by Kemerink and colleagues [43], the impact of trap density and trap depth on the slope of the *I*–*V* double logarithmic curve is orthogonal. Electron-only and hole-only devices were fabricated under the same conditions (Fig. S8). As shown in Fig. 2D and Fig. S9, the LTHJ OPD exhibits substantially reduced electron and hole trap densities compared to the BHJ OPD. In addition, the LTHJ structure introduces a limited number of deep traps, which effectively capture carriers and suppress nonequilibrium hopping between localized states, thereby contributing to dark current suppression [44,45]. Electrochemical impedance spectroscopy and Mott–Schottky analysis were employed to further quantify trap densities and depletion widths (Fig. 2E and Text S3). The *C*^−2^-*V* characteristics display linear behavior within a defined voltage range, where the slope is inversely proportional to the trap density, and the voltage-axis intercept corresponds to the device built-in potential [46]. Compared to BHJ OPDs, LTHJ OPDs exhibit a lower trap density and a larger depletion width (Fig. 2F). Therefore, the suppressed trap states and enhanced external charge blocking capability in the LTHJ structure contribute to the reduction of dark current.

A more comprehensive comparison of dark-state carrier transport across various OPD architectures is presented in Text S4 and Figs. S10 and S11. Notably, the LTHJ OPD features dual carrier blocking layers (Fig. 2G), simultaneously impeding both hole and electron injection. This structural advantage substantially suppresses dark current and improves sensitivity. The effectiveness of these barriers is directly reflected in the series resistance (*R*_s_) and shunt resistance (*R*_sh_) of the device. Specifically, a higher *R*_sh_ contributes to reducing leakage current, while a lower *R*_s_ facilitates enhanced rectification behavior. The LTHJ device exhibits the lowest *R*_s_ and highest *R*_sh_ among the tested configurations (Table S4, Fig. 2H, and Figs. S12 and S13), leading to an improved rectification ratio and reduced dark current. These trends are in agreement with both experimental measurements (Fig. S14) and theoretical predictions based on the diode equivalent circuit model (Text S5) [47]. To further analyze the effect of introducing PM_6_ and PY-IT layers on both sides of the BHJ layer in suppressing dark current, it is essential to consider the potential influence of intrinsic resistance, which may obscure the true source of dark current suppression. To eliminate the impact of intrinsic resistance and better understand the differences in dark current behavior (Fig. 1F), we employed the corrected current–voltage (*I*_corr_–*V*_rev_) analysis to provide a clearer interpretation of the dark current origins (Fig. 2I) [48]. *I*_corr_ is used to exclude the effects of series resistance (*R*_s_) and shunt resistance (*R*_sh_) (Text S6). The values of *R*_sh_ (Fig. S12) and *R*_s_ (Fig. S13) were extracted from the differential resistance derived from the dark current–voltage curves. In the low-bias region, the dark current is primarily governed by trap-assisted generation, where the trap density determines the carrier generation rate. As the voltage increases, the current transitions into the non-ohmic regime, dominated by space charge limited current (SCLC) injection. The reduced *I*_corr_ at low bias in the LTHJ OPD indicates effective trap-state passivation, consistent with our earlier trap density analysis. Furthermore, the delayed onset of SCLC suggests a higher injection barrier, corroborating the conclusion that the LTHJ structure effectively suppresses nonequilibrium carrier injection. Overall, the LTHJ OPD leverages a vdW multilayer architecture to enhance injection barriers, suppress disordered carrier injection, and reduce trap-state formation, thereby minimizing dark current from both injection and trap-assisted recombination.

### Mechanism for maintaining high photocurrent

A series of strategies have been proposed to suppress the dark current in OPDs, but these often affect the photoresponse of the OPD [28,48,49]. For example, the thick-film strategy limits the final responsivity due to the low carrier mobility in the semiconductor material [48]; the PHJ strategy, with fewer donor–acceptor interfaces, affects the effective separation and extraction of photogenerated carriers [49]; the polydimethylsiloxane (PDMS)-based printing of barrier layers introduces interface quality issues, which reduce the responsivity [28]. The strategy proposed in this paper effectively suppresses dark current while maintaining the original light response performance.

**Fig. 3.**
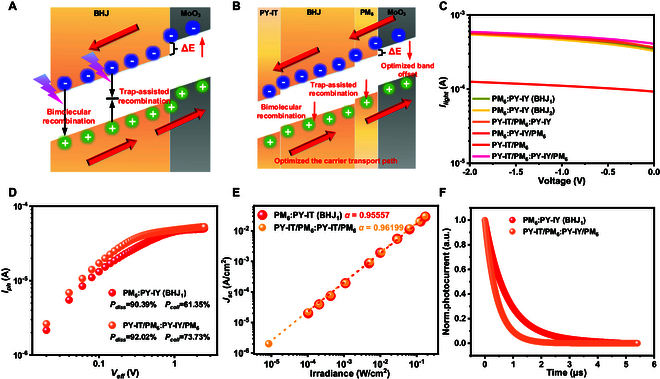
Maintain high photocurrent. (A) Bimolecular recombination and trap-assisted recombination processes in a BHJ OPD. (B) Operating mechanism of the LTHJ OPD. (C) *I*_light_ of different devices under 850-nm light illumination at reverse bias. (D) *I*_ph_ as a function of *V*_eff_. (E) *J*_SC_ versus light intensity. (F) TPC measurement.

The conventional BHJ structure often suffers from photocurrent losses arising from both trap-assisted recombination and bimolecular recombination (Fig. 3A). In contrast, the LTHJ structure is capable of effectively suppressing both types of recombination (Fig. 3B) while simultaneously enabling optimization of the band offset, thereby promoting charge carrier separation and transport. Specifically, the reduction in bimolecular recombination is achieved by improving interface quality and optimizing carrier transport paths. Trap-assisted recombination is minimized by reducing trap density through the WTP process. Additionally, the LTHJ structure introduces a small number of deep traps (Fig. 2D and Fig. S9). Photogenerated carriers are typically excited into high-energy states, which leads to trap-free transport behavior initially. These high-energy carriers are less likely to be captured by deep traps, allowing them to participate more effectively in conduction, thereby enhancing *I*_light_. The presence of an appropriate number of deep traps reduces *I*_dark_ while maintaining a high *I*_light_ [50]. Moreover, the LTHJ OPD exhibits a stronger built-in electric field, which facilitates exciton dissociation and improves carrier transport efficiency (Fig. 2E).

Under 850-nm illumination, the charge transport and extraction pathways of different device architectures are illustrated in Fig. S15. The PHJ structure exhibits limited exciton dissociation efficiency due to the restricted donor–acceptor interface area. In contrast, the BHJ structure substantially increases the donor–acceptor interface, effectively enhancing exciton dissociation. The LTHJ structure incorporates a stable vdW multilayer design, which effectively reduces material disorder and charge trapping, establishes a more ordered charge transport network, and minimizes recombination losses. Figure 3C presents the photocurrent characteristics of different device structures under reverse bias, where the LTHJ OPD demonstrates the highest photocurrent. Its photocurrent is 4.7 times higher than that of the PHJ structure and 1.08 times higher than that of the BHJ structure (850 nm, −2 V), indicating superior carrier generation and transport characteristics. Notably, at low bias, the photocurrent is strongly influenced by built-in electric field strength, carrier recombination, and interfacial quality [51,52]. The advantages of the LTHJ structure are particularly evident in the low-bias regime.

Figure 3D illustrates the relationship between photocurrent (*I*_ph_) and effective voltage (*V*_eff_), offering valuable insight into the processes of charge generation, exciton dissociation, and carrier extraction. *P*_diss_ and *P*_coll_ can be derived from the *I*_ph_–*V*_eff_ curves via the following [53]: $P_{\text{diss}}=\frac{I_{\mathrm{ph},\mathrm{SC}}}{I_{\mathrm{ph},\mathrm{sat}}}$, $P_{\text{coll}}=\frac{I_{\mathrm{ph},\text{max}}}{I_{\mathrm{ph},\mathrm{sat}}}$. Here, $I_{\mathrm{ph},\mathrm{SC}}$ is the short-circuit current, $I_{\mathrm{ph},\text{max}}$ is the current at the maximum power point, and $I_{\mathrm{ph},\mathrm{sat}}$ is the saturation current. As *V*_eff_ increases, *I*_ph_ rises in both BHJ and LTHJ devices, primarily due to the suppression of nongeminate recombination and enhanced charge collection efficiency. The LTHJ OPD demonstrates higher *P*_diss_ (92.02%) and *P*_coll_ (73.73%) compared to the BHJ counterpart (90.39% and 61.35%, respectively), indicating that the LTHJ architecture facilitates more efficient exciton dissociation and carrier extraction. According to the power-law dependence ($J_{\mathrm{sc}}\propto P_{\text{light}}^{\alpha }$) [46,54], the α values for BHJ and LTHJ OPDs are 0.95557 and 0.96199 (Fig. 3E), respectively, showing sublinear behavior due to bimolecular recombination.

The higher α value of the LTHJ OPD suggests reduced bimolecular recombination losses and improved charge transport, consistent with the *I*_ph_–*V*_eff_ analysis. In addition to bimolecular recombination, trap-assisted recombination also contributes to nongeminate losses. To probe this, transient photocurrent (TPC) measurements were conducted [46,55]. As shown in Fig. 3F, the LTHJ OPD exhibits a shorter charge extraction time compared to the BHJ OPD, confirming further suppression of trap-assisted recombination in the LTHJ configuration. Overall, the LTHJ OPD maintains superior photocurrent performance, unaffected by dark current suppression, and even slightly outperforms the BHJ device. This enhancement can be attributed to more efficient charge transport, reduced nongeminate recombination, and improved exciton dissociation dynamics.

### Comprehensive characterization of LTHJ OPD

**Fig. 4.**
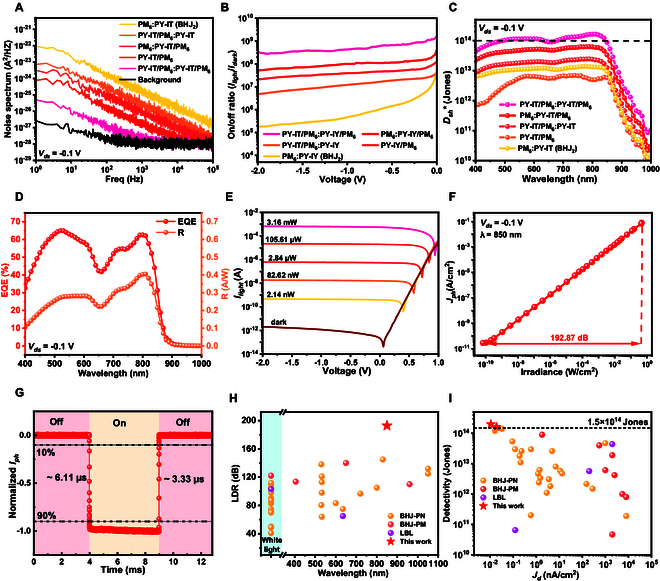
Photodetection performance. (A) Noise spectrum of different devices. (B) On/off ratio (*I*_light_/*I*_dark_) comparison among different devices. (C) *D*_sh_* of different devices. (D) EQE and *R* of the LTHJ OPD at −0.1 V. (E) *I*–*V* characteristics in the dark and at various intensities of 850 nm. (F) LDR of the LTHJ OPD. (G) Response times of the LTHJ OPD. (H) Comparison of LDR between all-polymer OPDs (Table S5) and the LTHJ OPD. (I) Comparison of *D*_sh_* and *I*_dark_ between all-polymer OPDs (Table S5) and the LTHJ OPD.

To gain a clearer and more detailed understanding of the detection performance of the LTHJ OPD, key device parameters were further investigated in Fig. 4 to evaluate its application potential. Figure 4A illustrates the noise spectral density as a function of frequency for different devices, showing a trend consistent with the variation in dark current (Fig. 1F). Compared to the conventional BHJ structure, the LTHJ structure substantially reduces noise levels, exhibiting a lower noise spectral density than the PHJ structure. Notably, the noise curve of the LTHJ structure approaches the background noise level, indicating that it has reached the detection limit of the measurement system. Theoretically, this suggests that the LTHJ structure should achieve an even lower noise spectral density. This is also a key factor contributing to the lower D* value obtained from noise spectral density measurements compared to *D*_sh_* calculated from dark current. The relatively high dark current in the BHJ structure results in a low on/off ratio. Introducing a pure donor or acceptor layer on either side of the BHJ effectively reduces the dark leakage current while preserving good photoresponse, thus improving the on/off ratio. Based on this approach, the LTHJ structure achieves the lowest dark current and thus the best switching performance, with an on/off ratio exceeding 10^9^ under continuous 850-nm illumination (~142.5 mW/cm^2^) at 20 °C and 34% RH (Fig. 4B). Figure 4C and Fig. S16 show the *D*_sh_* and D* of different devices, respectively. Due to the lower photoresponse of the PHJ OPD, it exhibits the lowest detectivity, while the BHJ OPD achieves slightly higher detectivity. Introducing either a donor or acceptor layer on both sides of the BHJ structure effectively improves the device’s detectivity, with the LTHJ OPD demonstrating the best performance. Across a broad spectral range, its detectivity exceeds 10^14^ Jones, with a peak value of 1.64 × 10^14^ Jones at 810 nm, highlighting its outstanding photodetection capabilities.

The external quantum efficiency (EQE) and *R* spectra (Fig. 4D) demonstrate outstanding NIR performance, achieving over 60% EQE and an *R* exceeding 0.4 A/W. The LTHJ OPD exhibits an exceptionally low dark current, maintaining values below 1 pA at low bias (−0.8 V < *V*_ds_ < 0 V), and even at −2 V, the dark current remains as low as 2 pA, which is among the best-reported performances in all-polymer photodetectors to date (Fig. 4E). The *I*–*T* curve at 0 V further confirms the ultra-low dark current of the LTHJ OPD (Fig. S17). As shown in Fig. S18, the *D*_sh_* decreases monotonically with increasing reverse bias. At 0-V and −0.1-V bias, the peak *D*_sh_* values reach 2 × 10^14^ and 1.64 × 10^14^ Jones, respectively, and maintain values above 10^14^ Jones across a broad spectral range. Even at a relatively high reverse bias of −2 V, the device retains a detectivity of 8.93 × 10^13^ Jones, demonstrating excellent sensitivity. The noise spectral density of the LTHJ OPD under different voltages is shown in Fig. S19. Figure S20 presents the D* calculated from the noise spectral density. Generally, D* is slightly lower than *D*_sh_* due to the inclusion of additional noise sources in the noise spectral density calculation. Furthermore, the LTHJ OPD’s noise level is already close to the instrument’s background noise (Fig. 4A). These combined effects result in *D*_sh_* being approximately one order of magnitude higher than D*. Benefiting from the ultra-low dark current and excellent photocurrent characteristics, the LTHJ OPD achieves an impressive LDR of 192.87 dB (Fig. 4F), ensuring superior weak light detection capability (Fig. S21).

The transient response characteristics of the LTHJ OPD are depicted in Fig. 4G, showing rise and fall times of 6.11 and 3.33 μs, respectively, which meet the requirements for real-time high-speed photodetection applications. As shown in Fig. S22, the −3-dB bandwidth of the LTHJ OPD is approximately 173 kHz. Additionally, Fig. S22B further illustrates the photoresponse signals of the LTHJ OPD at 640 Hz and 160 kHz. Compared to conventional all-polymer photodetectors, the LTHJ structure enhances device performance, demonstrating superior performance in key parameters such as LDR, dark current, specific detectivity, and responsivity (Fig. 4H and I and Figs. S23 and S24). Moreover, the performance of the LTHJ OPD in terms of dark current and detectivity surpasses that of conventional inorganic photodetectors such as Si PD and InGaAs PD (Fig. S25 and Text S7). These advantages highlight the great potential of LTHJ OPDs for high-sensitivity photodetection applications.

**Fig. 5.**
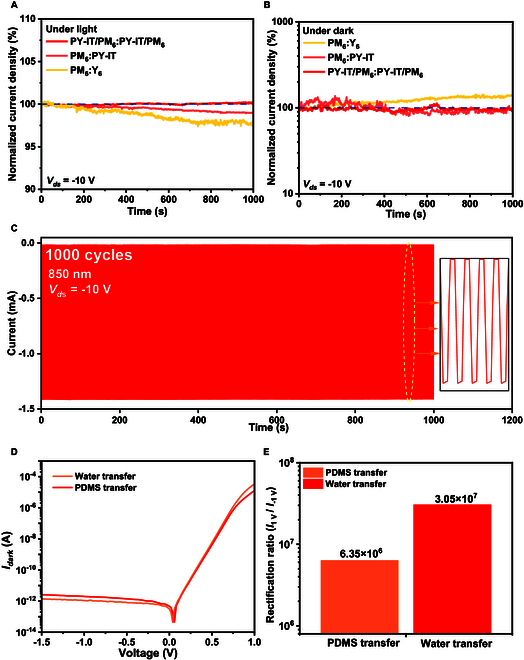
Stability analysis. (A and B) Electrical stability of different devices under a continuous −10-V bias, both (A) under illumination and (B) in the dark. (C) *I*–*T* response of the device under 850-nm cyclic illumination for 1,000 cycles at *V*_ds_ = −10 V. (D and E) Dark current characteristics and rectification ratios of LTHJ OPDs fabricated using PDMS and WTP.

One of the key advantages of all-polymer OPDs is their superior electrical stability, and the LTHJ structure further enhances this property (Fig. 5A and B and Fig. S26). Figure 5C further demonstrates the stable operation of LTHJ OPD over 1,000 light pulses. Furthermore, the LTHJ structure exhibits superior long-term stability compared to the BHJ structure (Fig. S27). PDMS transfer printing can also be used to fabricate LTHJ OPDs; however, it may introduce substantial surface irregularities (Fig. S28) and increased roughness, which can adversely affect OPD performance (Texts 8 and 9). Moreover, achieving a comparable dark current with PDMS transfer requires a thicker film, which inevitably reduces the rectification ratio (Fig. 5D and E) and responsivity of the OPD.

### Application: O-ISAC

**Fig. 6.**
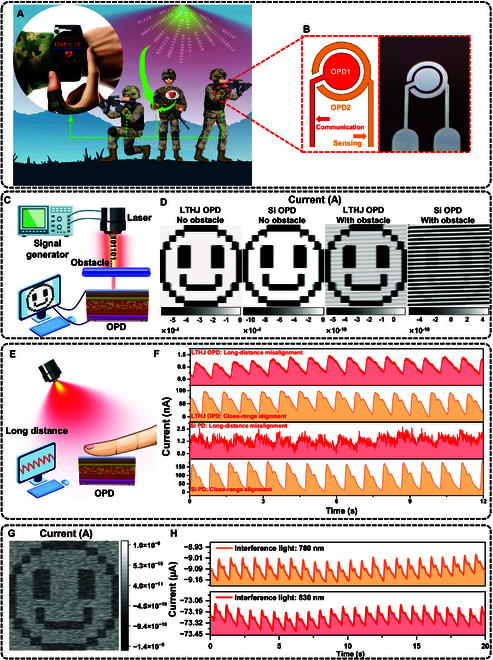
Implementation of O-ISAC using LTHJ OPDs. (A) Potential applications of O-ISAC utilizing LTHJ OPDs, including remote health monitoring and tactical communication. (B) Schematic (left) and optical image (right) of the O-ISAC system based on LTHJ OPDs. (C) Schematic of OWC with an obstacle. (D) OWC with 850-nm light produces a “smile” image using LTHJ OPD and Si PD. (E) Schematic of PPG under long-distance misalignment. (F) PPG signals collected using LTHJ OPD and Si PD under close-range alignment and long-distance misalignment with an 850-nm NIR light-emitting diode. (G) OWC under dynamic obstacle movement. (H) PPG signals acquired under interference illumination.

O-ISAC enables the deep integration of sensing and communication through optical methods. Compared to traditional radio frequency (RF)-based integrated sensing and communication technologies, O-ISAC offers wider bandwidth, lower power consumption, and stronger anti-interference capabilities, making it highly promising for future wireless communication and intelligent sensing systems (Text S10). Leveraging the high sensitivity of LTHJ OPD, O-ISAC facilitates efficient, low-power remote PPG monitoring and OWC (Fig. 6A), enabling precise real-time sensing of soldiers’ physiological conditions in complex environments while transmitting critical data via optical communication (e.g., the “Retreat!” command from headquarters). Furthermore, by utilizing NIR optical communication technology, O-ISAC (Fig. 6B and Fig. S29) enables covert, high-speed data transmission on the battlefield, effectively mitigating the risk of RF signal interception and jamming by adversaries. The OPD used for communication (Com-OPD) and the OPD used for sensing (Sen-OPD) exhibit comparable optoelectronic characteristics (Fig. S30).

Currently, Si PD is the primary choice for commercial applications, widely utilized in OWC and PPG. Figure 6C and Fig. S31A illustrate the schematic of the OWC. When there is no obstacle in the optical path, the transmitted light signal can directly reach the OPD (Fig. S31A). However, when an obstacle is present, only a weakened light signal can pass through the OPD (Fig. 6C), with the obstacle acting as an amplitude-attenuating medium. Figure 6D illustrates the performance of LTHJ OPD and Si PD in obstacle-free and obstacle-penetrating OWC. Under standard obstacle-free conditions, both LTHJ OPD and Si PD successfully transmit the intended information, represented by a “smile” pattern. However, in the presence of an obstacle, the LTHJ OPD still effectively receives and reconstructs the transmitted information, whereas the Si PD signal is heavily interfered with by noise. This result highlights the superior sensitivity of the LTHJ OPD in optical communication, particularly in challenging environments where efficient and stable signal transmission is crucial. Figure 6E and Fig. S31B demonstrates the application of OPD in PPG signal acquisition. Light transmission through biological tissue is substantially affected by attenuation, diffraction, and scattering, leading to weak physiological signal detection. Additionally, longer optical transmission paths introduce additional optical losses. For close-range alignment PPG measurements, both sensors successfully capture distinct systolic and diastolic peaks, as shown in Fig. 6F and Fig. S32. When measuring long-distance misalignment PPG signals, the LTHJ OPD still provides a clear and recognizable PPG signal, while the Si PD signal experiences severe distortion, with noise levels approaching those of the effective signal. Fast Fourier transform analysis of the PPG signal obtained from the LTHJ OPD shows a heart rate of approximately 79.5 beats per minute (Fig. S33). Further studies demonstrate that the LTHJ OPD is capable of performing reliably even in complex environments with moving obstacles and interfering light sources (Fig. 6G and H and Figs. S34 and S35).

Flexible LTHJ OPDs fabricated on polyethylene terephthalate (PET) substrates via WTP exhibit high sensitivity and ultra-low dark current, enabling applications in wearable monitoring, OWC, and optical imaging (Figs. S36 to S40). However, their flexibility is limited to preliminary bendability due to the intrinsic rigidity of PET. To verify the versatility of this strategy, we fabricated devices based on PM_6_, PYF-T-o, PTB7-TH, and PY-IT using the same method (Figs. S41 and S42). Compared to BHJ OPDs, LTHJ OPDs exhibit a substantial reduction in dark current and achieve a detectivity exceeding 10^14^ Jones. While these results strongly support the effectiveness of the LTHJ structure, further testing with additional polymer combinations is necessary to fully evaluate its universal applicability.

## Conclusion

In this work, a multilayer vdW LTHJ structure was fabricated using WTP technology, successfully enhancing the overall performance of all-polymer NIR OPDs. This structure effectively reduces defect states and leakage current while improving interfacial quality, optimizing charge transport pathways, and facilitating carrier dissociation and extraction. As a result, the LTHJ OPD achieves an ultra-low dark current in the pA range and an on/off ratio exceeding 10^9^, all while maintaining high photoresponse. Additionally, the device demonstrates a specific detectivity exceeding 10^14^ Jones in the NIR region and an exceptional LDR of 192.87 dB, achieving the state-of-the-art performance among all-polymer OPDs. Leveraging this high-sensitivity OPD, we further demonstrate O-ISAC applications, including obstacle-penetrating OWC and long-distance misalignment PPG monitoring, highlighting its potential in next-generation flexible wearable electronics and complex environment communication.

This study demonstrates the effectiveness of the LTHJ structure in specific polymer systems; however, future work should extend its application to a broader range of polymer materials to further validate its generality and stability. While short-term tests, such as 1,000-cycle evaluations, have been conducted, the long-term stability of the device under environmental stressors, including humidity, temperature cycling, and oxygen exposure, has yet to be assessed. Future research should prioritize evaluating the device’s performance under these conditions and explore additional strategies to enhance its stability. Regarding the industrial application of WTP technology, further investigations should focus on its scalability, optimizing the transfer process, improving efficiency, and reducing production costs. In conclusion, future efforts should focus on material expansion, long-term environmental stability, large-scale production feasibility, and practical application integration to facilitate the commercialization of all-polymer photodetectors based on the LTHJ structure.

## Materials and Methods

### Materials

All chemicals and reagents were obtained from commercial suppliers and used in their original form without any additional purification. PM_6_ and PTB7-TH were sourced from Organtec Ltd., while PY-IT and PYF-T-o were supplied by eFlexPV. MoO_3_ was acquired from Luminescence, and PEIE was procured from Sigma-Aldrich.

### Device fabrication

The fabrication of OPDs was conducted on ITO/glass substrates. Subsequent to a 20-min treatment with ultraviolet (UV)–ozone, the ITO/glass substrates that had been precleaned were transferred into a glovebox. PEIE solution (37 wt % in H_2_O) was diluted to 0.1 wt % using 2-propanol and stirred magnetically for 12 h. Subsequently, the diluted PEIE solution was filtered through a polytetrafluoroethylene membrane (pore size: 0.22 μm) and spin-coated onto precleaned ITO/glass substrates at 5,000 rpm for 60 s. The resulting films were then annealed on a hot plate at 100 °C for 5 min. The active layer was prepared on the PEIE layer. The 30-nm PY-IT layer was deposited by spin-coating from a 6 mg ml^−1^ solution in chloroform, followed by thermal annealing at 100 °C for 5 min. A 100-nm-thick PM_6_:PY-IT (1:1) BHJ film was transferred onto the PY-IT layer. Specifically, 35 μl of PM6:PY-IT solution (30 mg/ml) in chlorobenzene was dropped onto a water surface, forming a uniform film after drying completely. The sample was then gently pressed onto the BHJ film surface for transfer, then evacuated at −0.1 MPa for 2 min, and annealed on a hot plate at 100 °C for 5 min. A 25-nm-thick PM_6_ film was transferred onto the BHJ layer by dropping 15 μl of PM_6_ solution (20 mg/ml) in chlorobenzene onto a water surface, then evacuated at −0.1 MPa for 2 min, and annealed on a hot plate at 100 °C for 5 min. Finally, a 10-nm MoO_3_ layer and a 100-nm Ag electrode were successively thermally evaporated onto the organic layers through shadow masks, defining an active device area of 0.02 cm^2^.

### Device characterization

Electrical and optoelectronic characteristics of the devices were recorded using an FS-Pro and a Keithley 4200-SCS semiconductor parameter analyzer. The photocurrent at 850 nm was generated by a diode laser source (EGI-D2-40, NKT Photonics) with intensity adjusted through a tunable attenuator (FTB-3500-Cl-Ei, EXFO). EQE characterization was performed using a monochromatic xenon lamp setup (Gloria-X150A, Zolix), with calibration referenced to a commercial silicon detector (HAMAMATSU, S1337-1010BQ). Atomic force microscope (AFM) images were obtained using an MFP-3D-BIO system (Asylum Research), and absorption spectra were recorded on a U-3010 UV spectrometer (Hitachi). XPS measurements were conducted using an ESCALAB 250Xi spectrometer (Thermo Scientific, USA). Contact angle and surface tension measurements were performed on an SZ-CAMC32 instrument (Shanghai Xuanzhun).

Additional experimental details can be found in Texts S1 and S11.

### Industrial feasibility of the WTP process

The WTP process offers remarkable advantages in achieving high-quality multilayer organic film stacking, yet its scalability and industrial compatibility remain critical concerns. At present, the technique largely relies on manual or semiautomated transfer steps, which constrain throughput and reproducibility in large-area manufacturing. Future advancements may focus on developing automated transfer platforms integrated with optical/machine-vision inspection and artificial intelligence (AI)-driven dynamic correction to enable full-process automation and minimize human intervention. Compared with evaporation or spray-coating methods, WTP eliminates the need for high-vacuum deposition or orthogonal solvents, thereby simplifying equipment requirements, reducing chemical waste, and lowering production costs. Moreover, its excellent compatibility with curved and flexible substrates further expands its application potential. Overall, despite current challenges in automation, WTP remains a promising, cost-effective, and scalable pathway for the fabrication of high-performance multilayer organic optoelectronic devices, with strong prospects for commercialization.

## Data Availability

The data supporting the results of this study can be obtained from the corresponding author upon reasonable request.

## Supplementary Materials

Supplementary 1 Texts S1 to S11 Tables S1 to S5 Figs. S1 to S42 References [56–105]
