# Supplementary material for: Ultra-Sensitive All-Polymer Near-Infrared Photodetectors via Van der Waals Layered Triple Heterojunction
Source: Research (Wash D C). 2025 Oct 3;8:0939. doi: 10.34133/research.0939 (PMC12491782; doi:10.34133/research.0939)
Supplement: Supplementary 1 — Texts S1 to S11 Tables S1 to S5 Figs. S1 to S42 References [56–105] [file research.0939.f1.zip › Figure S35.pdf]

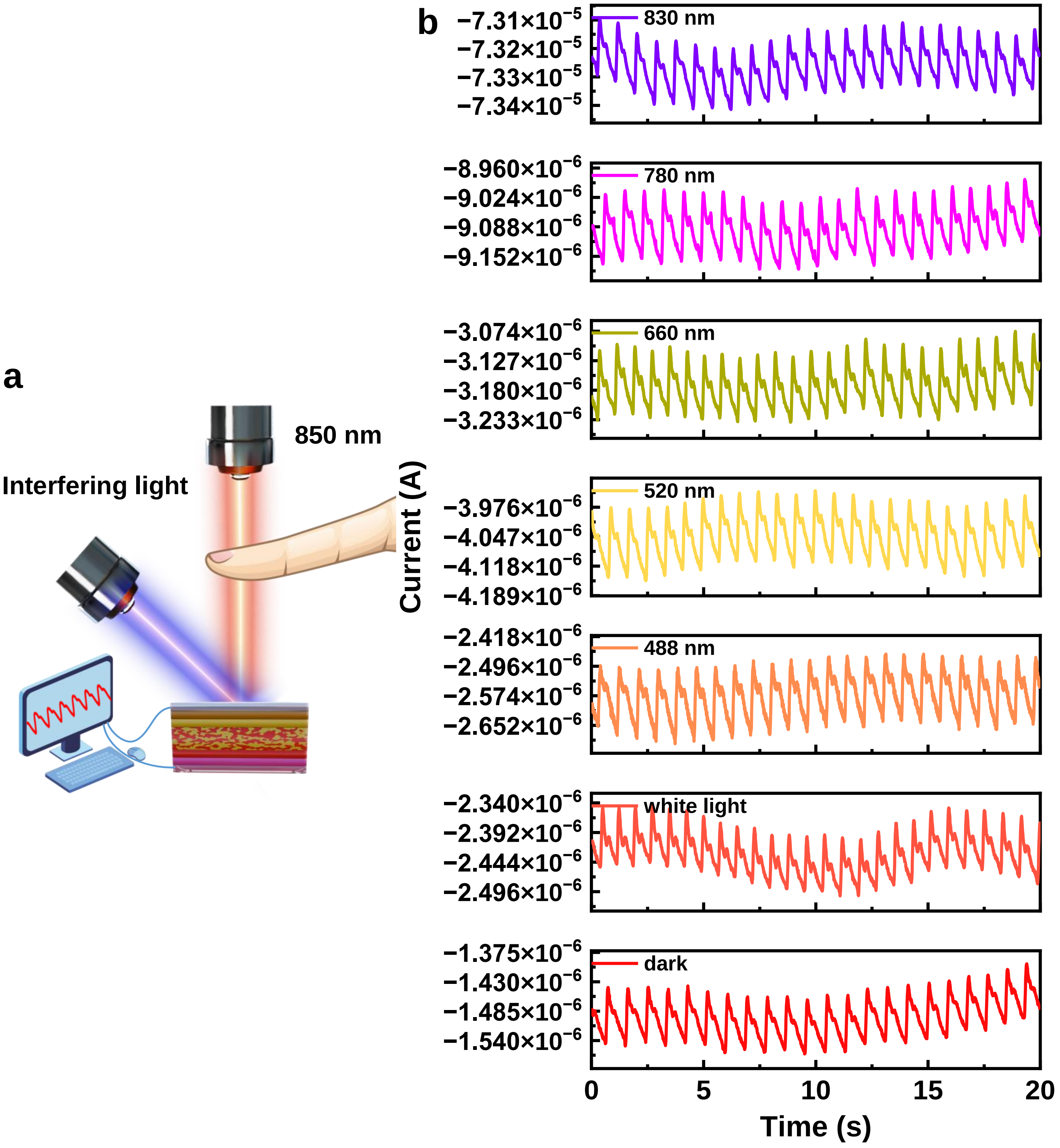
a
850 nm
Interfering light
b
Current (A)
Time (s)
830 nm
780 nm
660 nm
520 nm
488 nm
white light
dark
−7.31×10^−5
−7.32×10^−5
−7.33×10^−5
−7.34×10^−5
−8.960×10^−6
−9.024×10^−6
−9.088×10^−6
−9.152×10^−6
−3.074×10^−6
−3.127×10^−6
−3.180×10^−6
−3.233×10^−6
−3.976×10^−6
−4.047×10^−6
−4.118×10^−6
−4.189×10^−6
−2.418×10^−6
−2.496×10^−6
−2.574×10^−6
−2.652×10^−6
−2.340×10^−6
−2.392×10^−6
−2.444×10^−6
−2.496×10^−6
−1.375×10^−6
−1.430×10^−6
−1.485×10^−6
−1.540×10^−6
0
5
10
15
20
