# Supplementary material for: Ultra-Sensitive All-Polymer Near-Infrared Photodetectors via Van der Waals Layered Triple Heterojunction
Source: Research (Wash D C). 2025 Oct 3;8:0939. doi: 10.34133/research.0939 (PMC12491782; doi:10.34133/research.0939)
Supplement: Supplementary 1 — Texts S1 to S11 Tables S1 to S5 Figs. S1 to S42 References [56–105] [file research.0939.f1.zip › Figure-Supporting 1.pptx]

## Slide 1
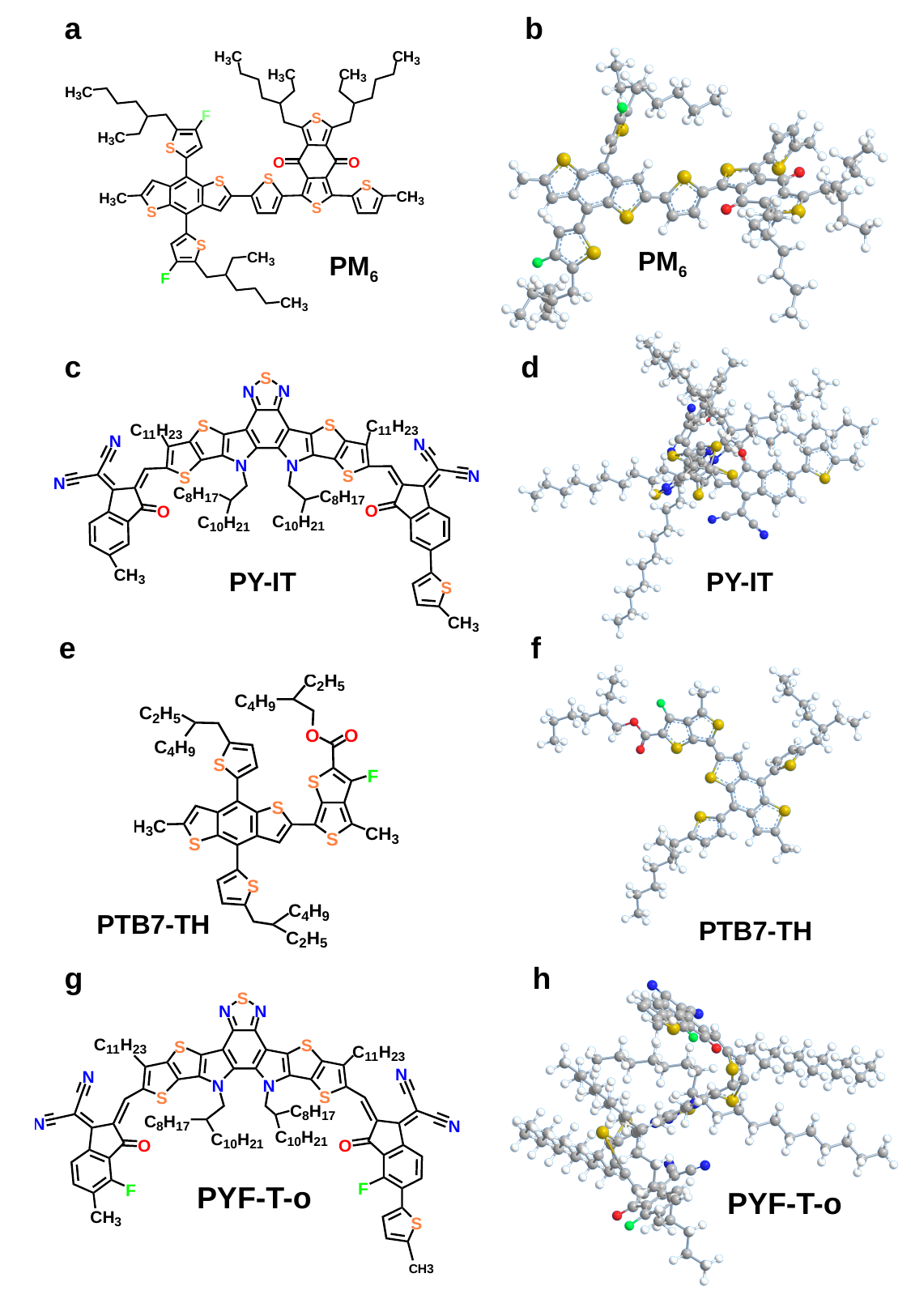

a
b
PM6
PM6
c
d
PY-IT
PY-IT
e
f
PTB7-TH
PTB7-TH
g
h
PYF-T-o
PYF-T-o

## Slide 2
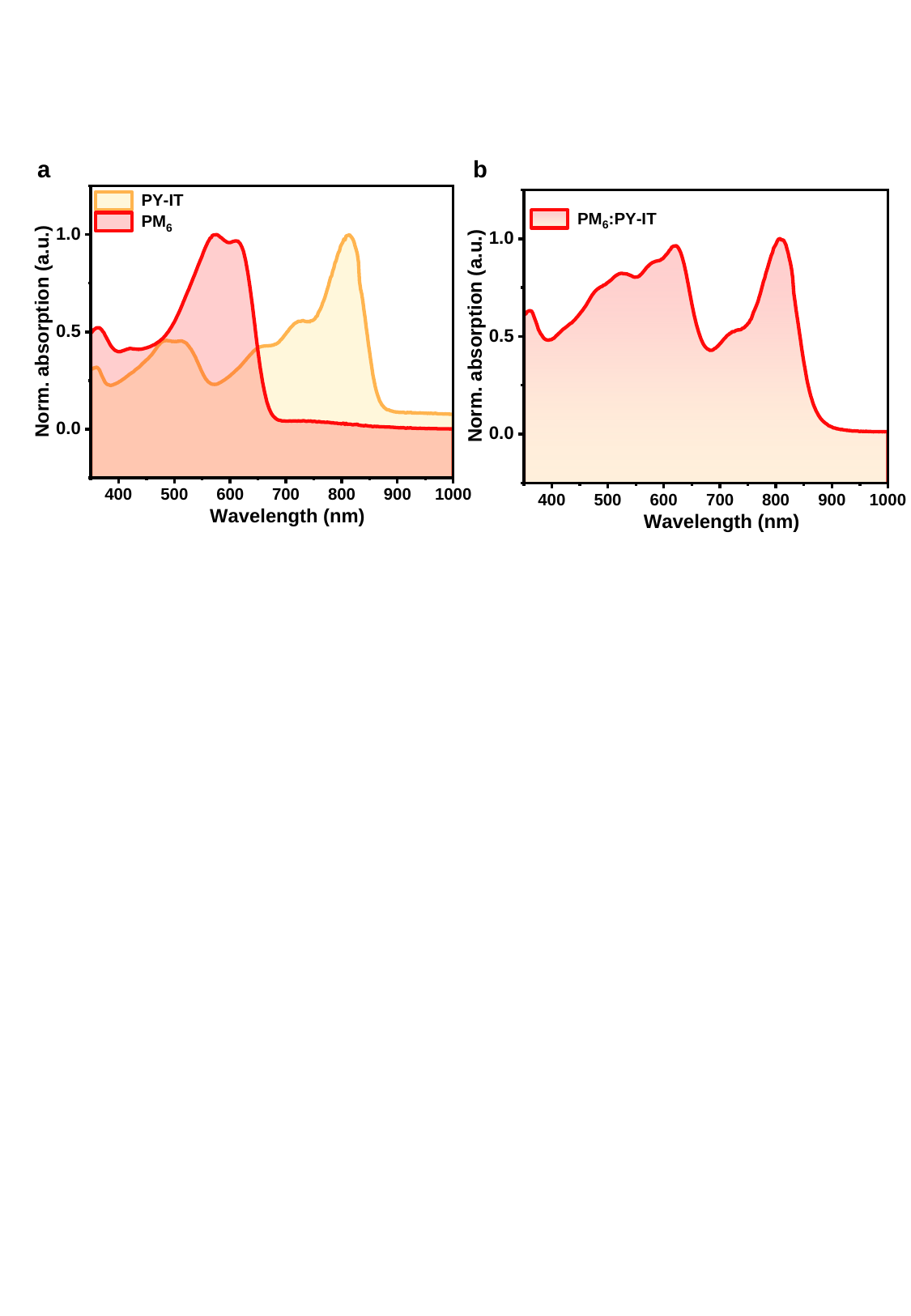

a
b

## Slide 3
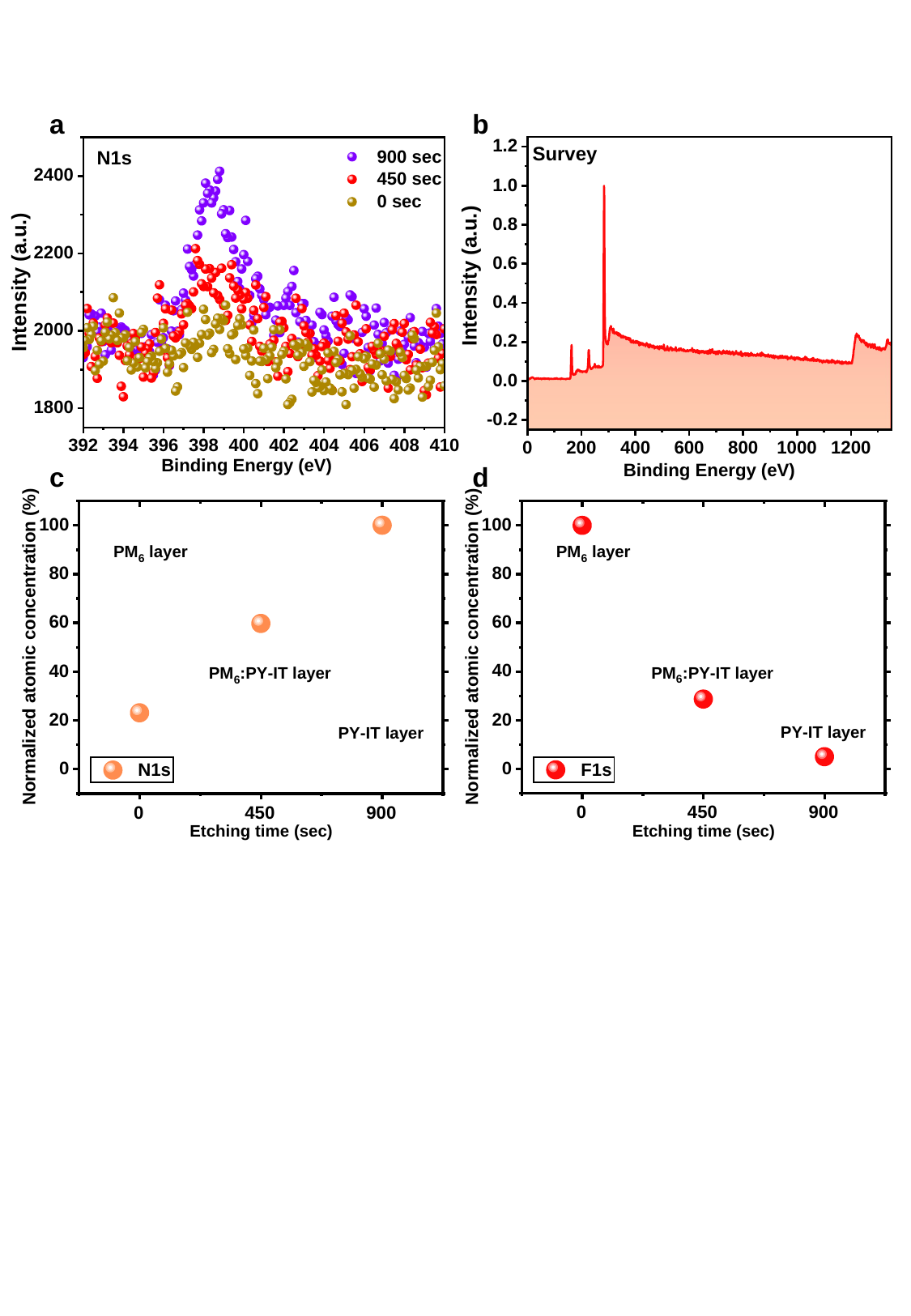

a
b
c
d

## Slide 4
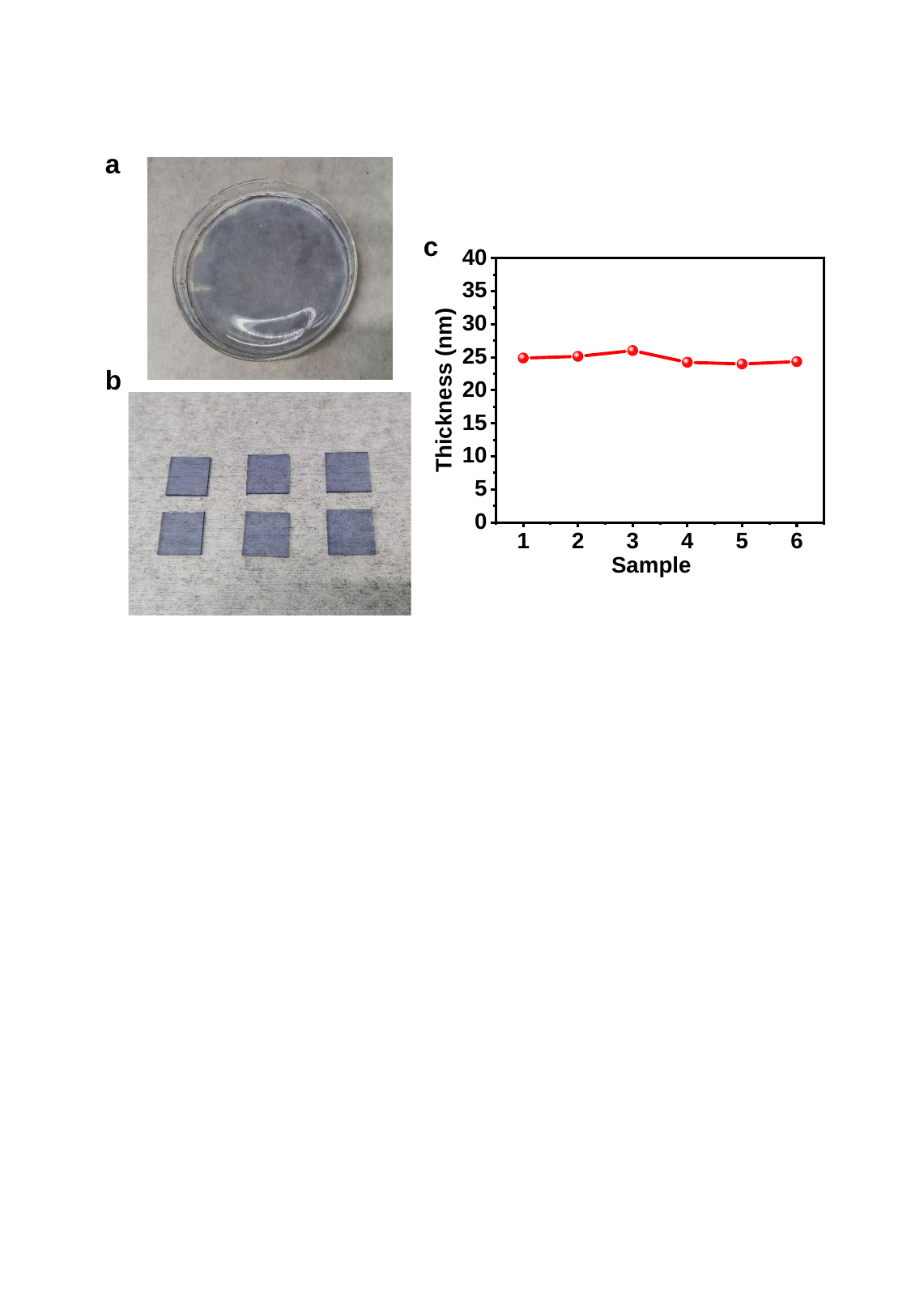

a
c
b

## Slide 5
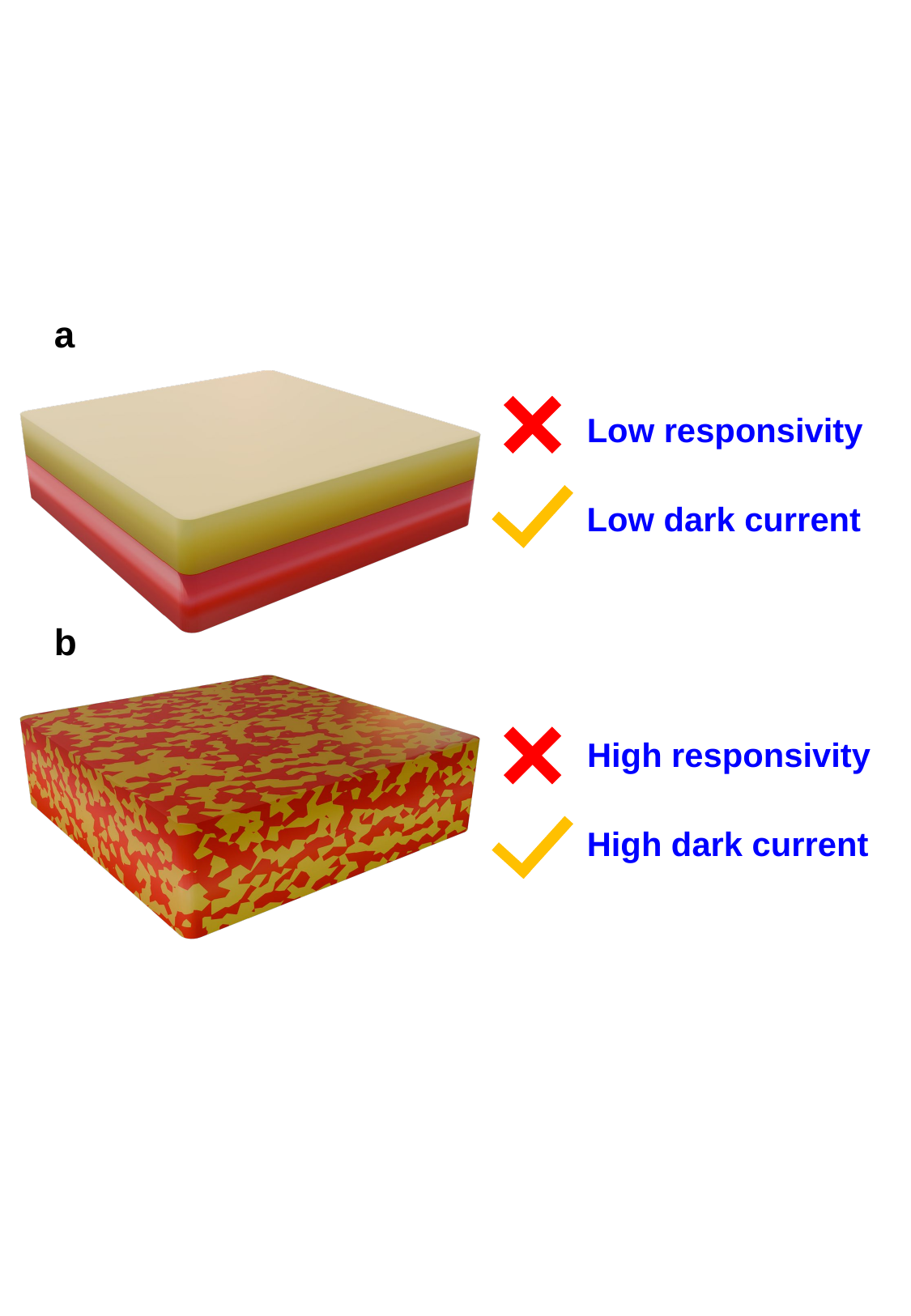

a
Low responsivity
Low dark current
b
High responsivity
High dark current

## Slide 6
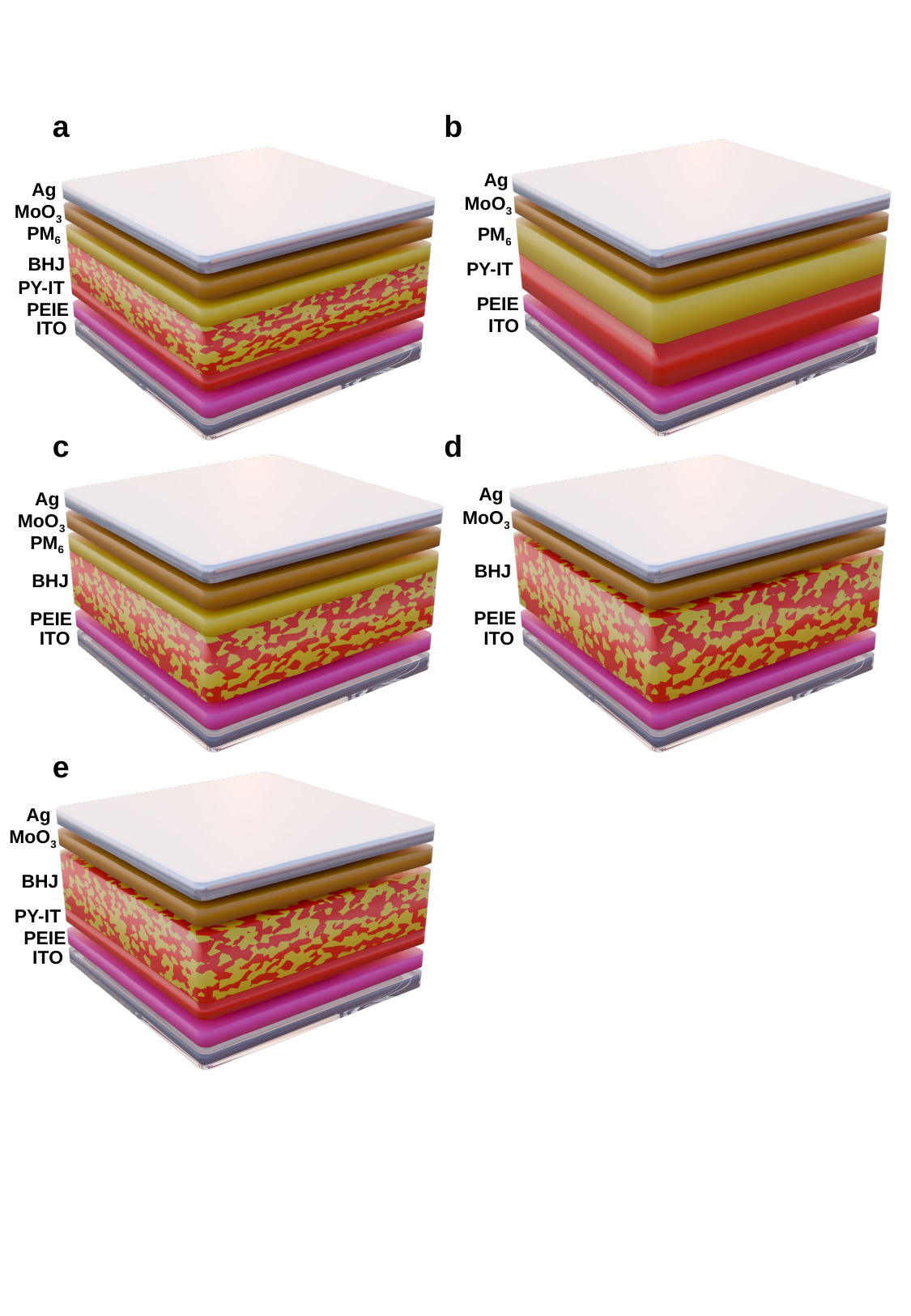

a
b
Ag
Ag
MoO3
MoO3
PM6
PM6
BHJ
PY-IT
PY-IT
PEIE
PEIE
ITO
ITO
c
d
Ag
Ag
MoO3
MoO3
PM6
BHJ
BHJ
PEIE
PEIE
ITO
ITO
e
Ag
MoO3
BHJ
PY-IT
PEIE
ITO

## Slide 7
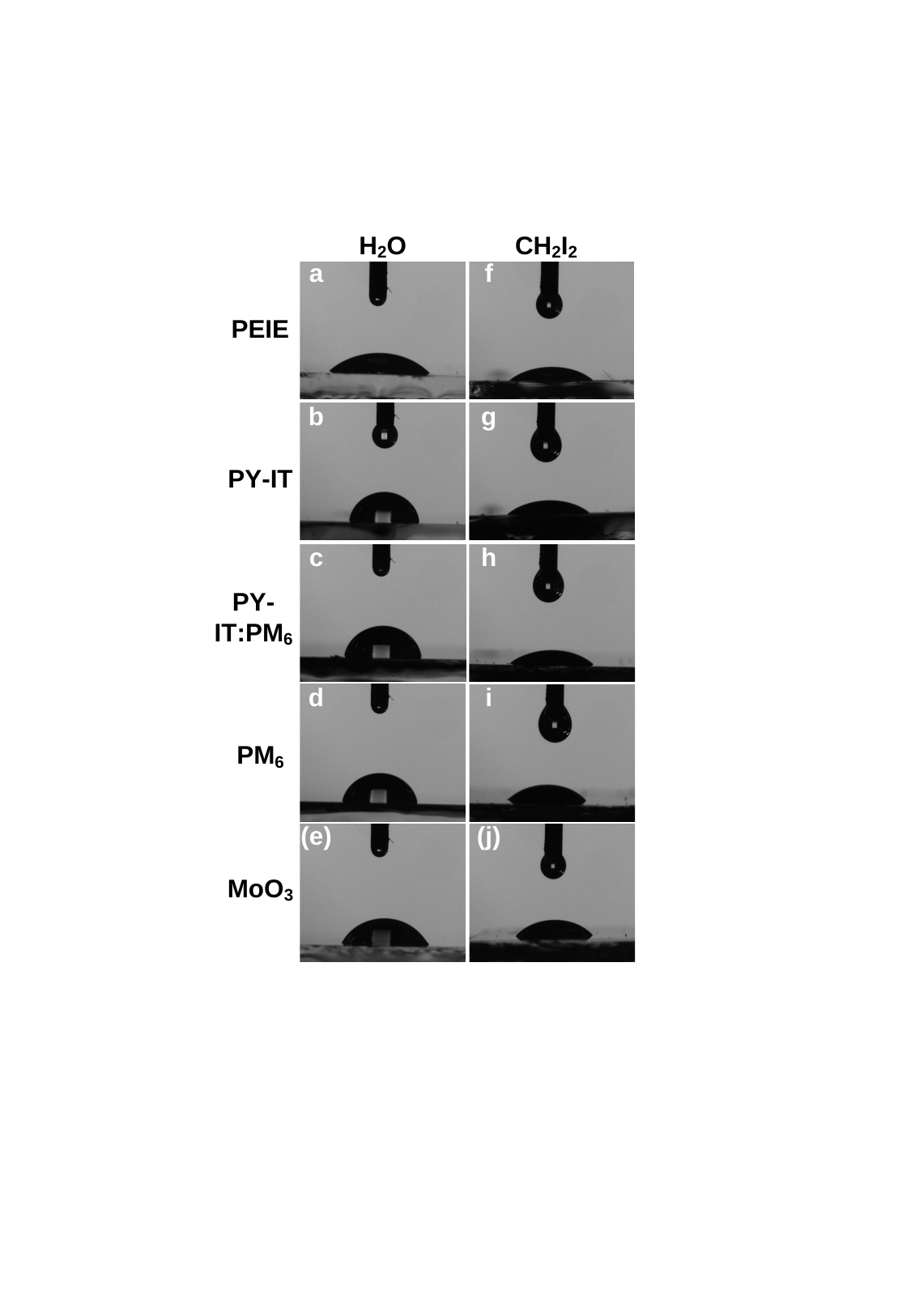

## Slide 8
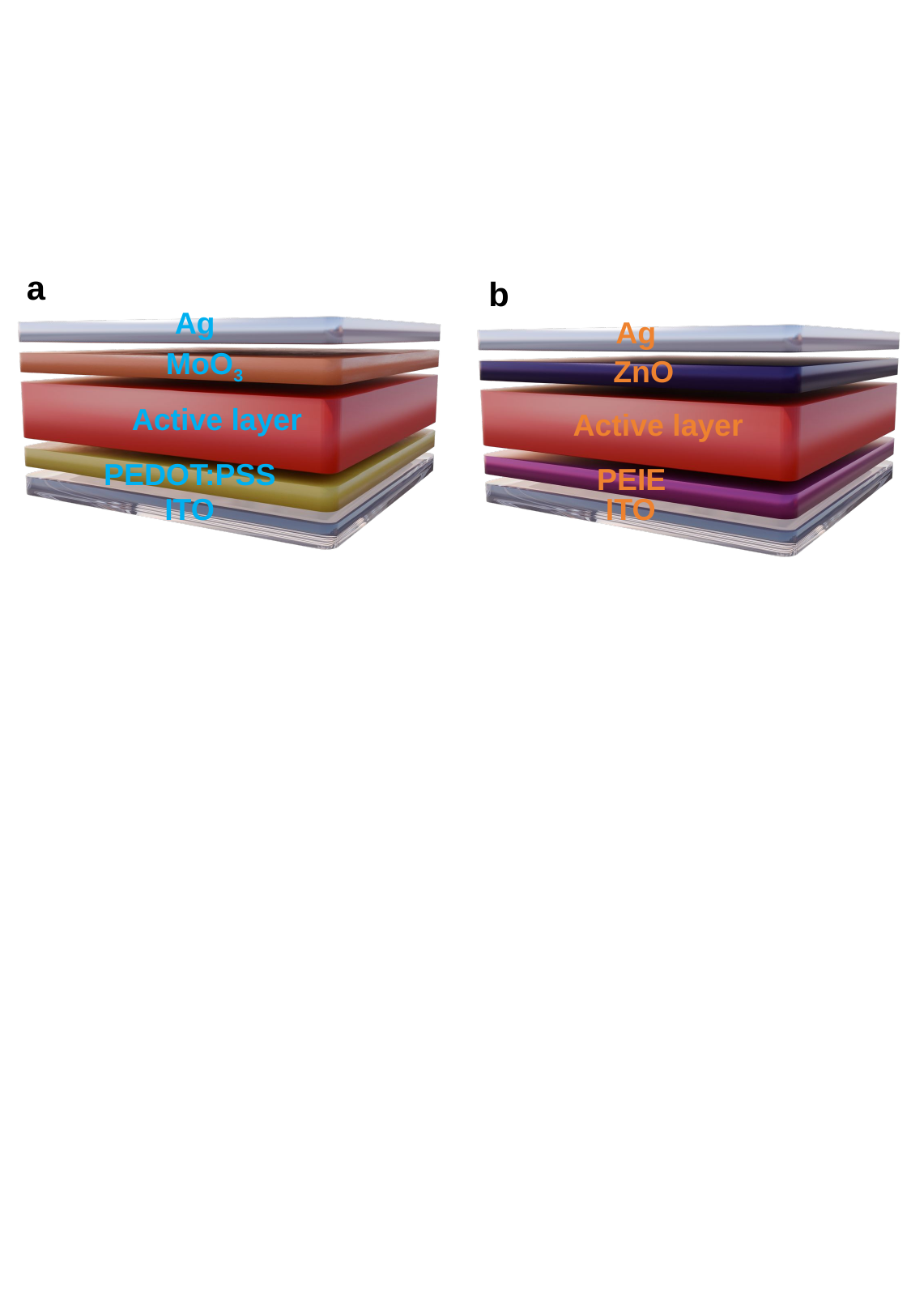

a
b
Ag
Ag
MoO3
ZnO
Active layer
Active layer
PEDOT:PSS
PEIE
ITO
ITO

## Slide 9
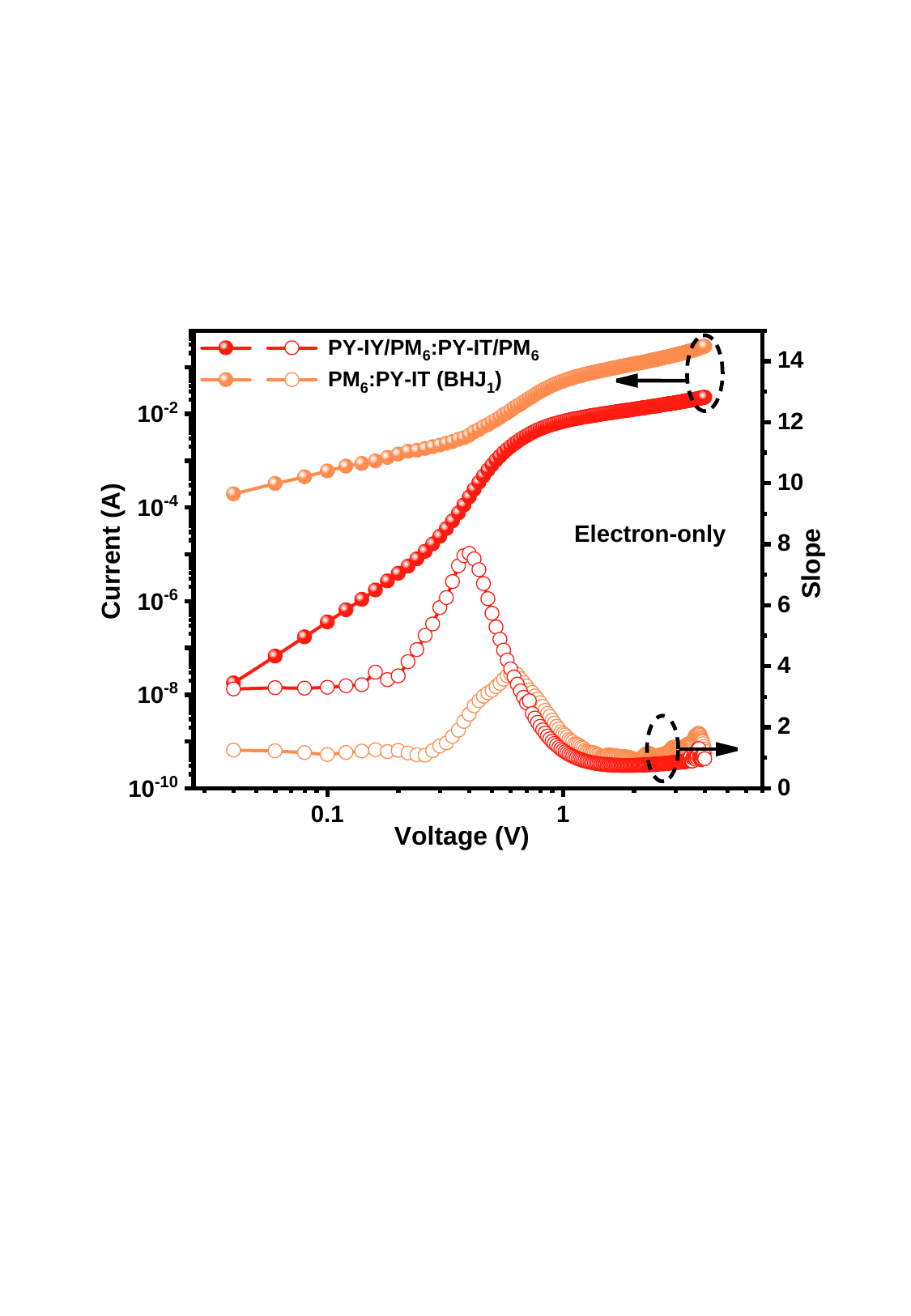

## Slide 10
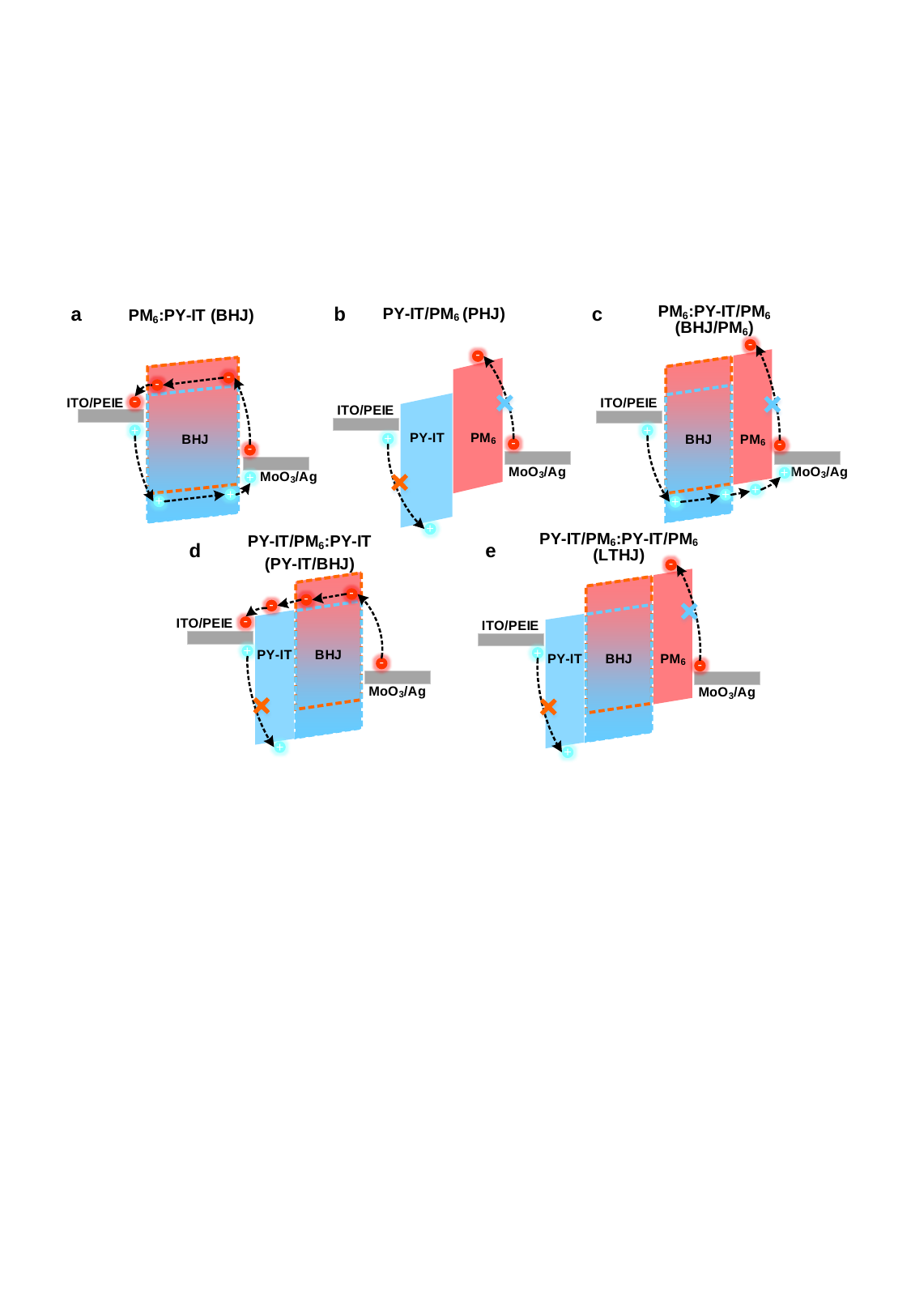

## Slide 11
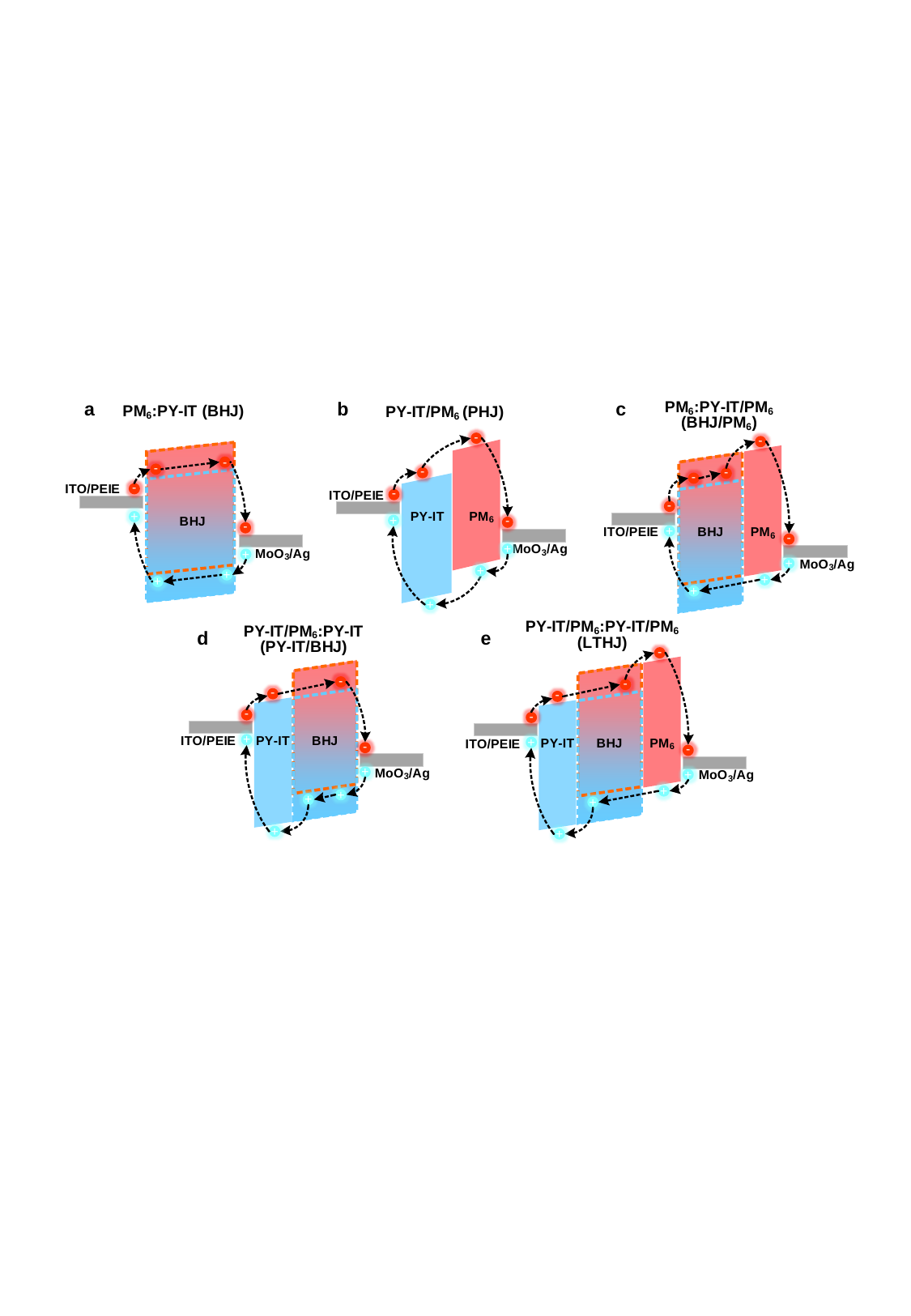

## Slide 12
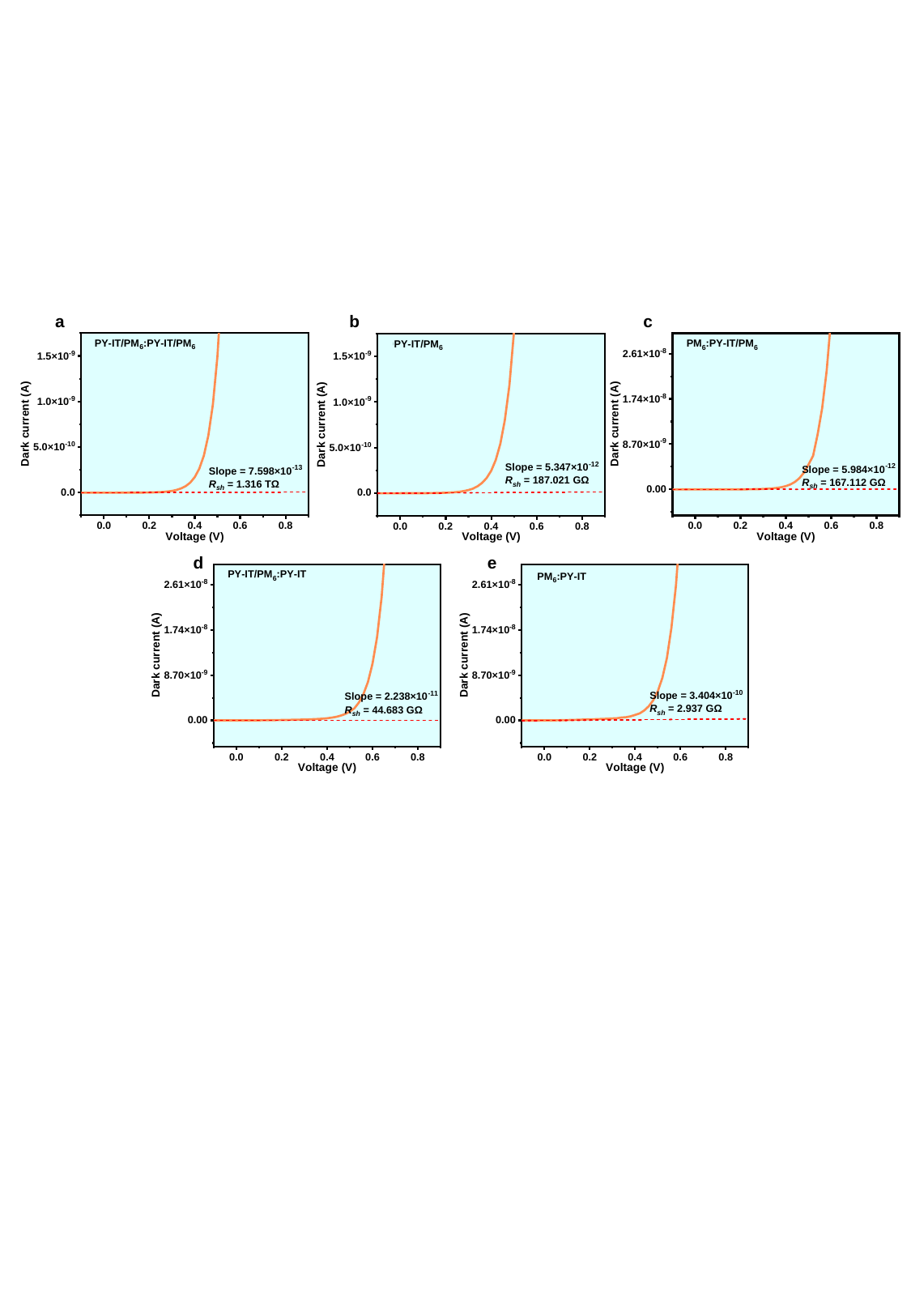

a
b
c
d
e

## Slide 13
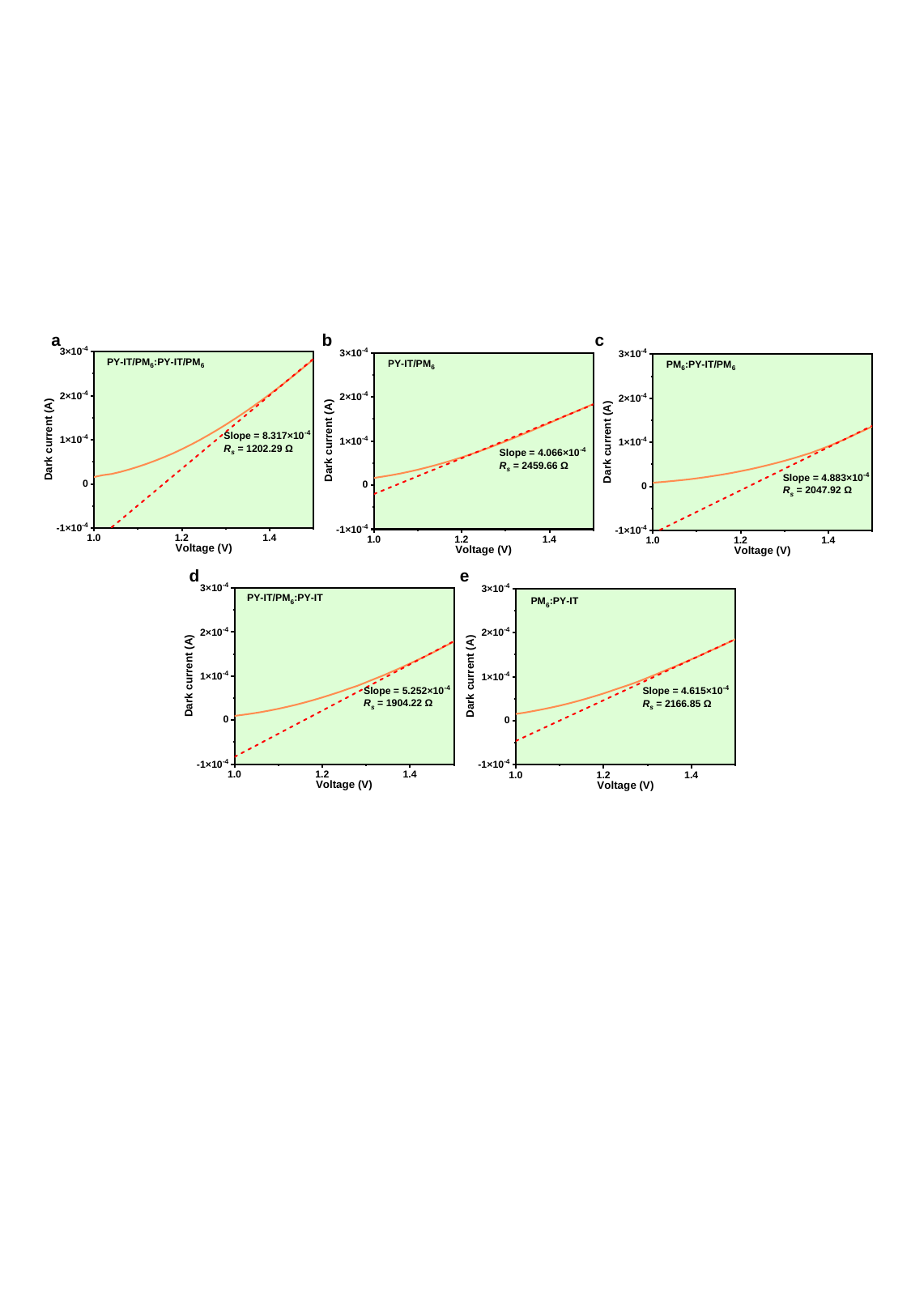

a
b
c
d
e

## Slide 14
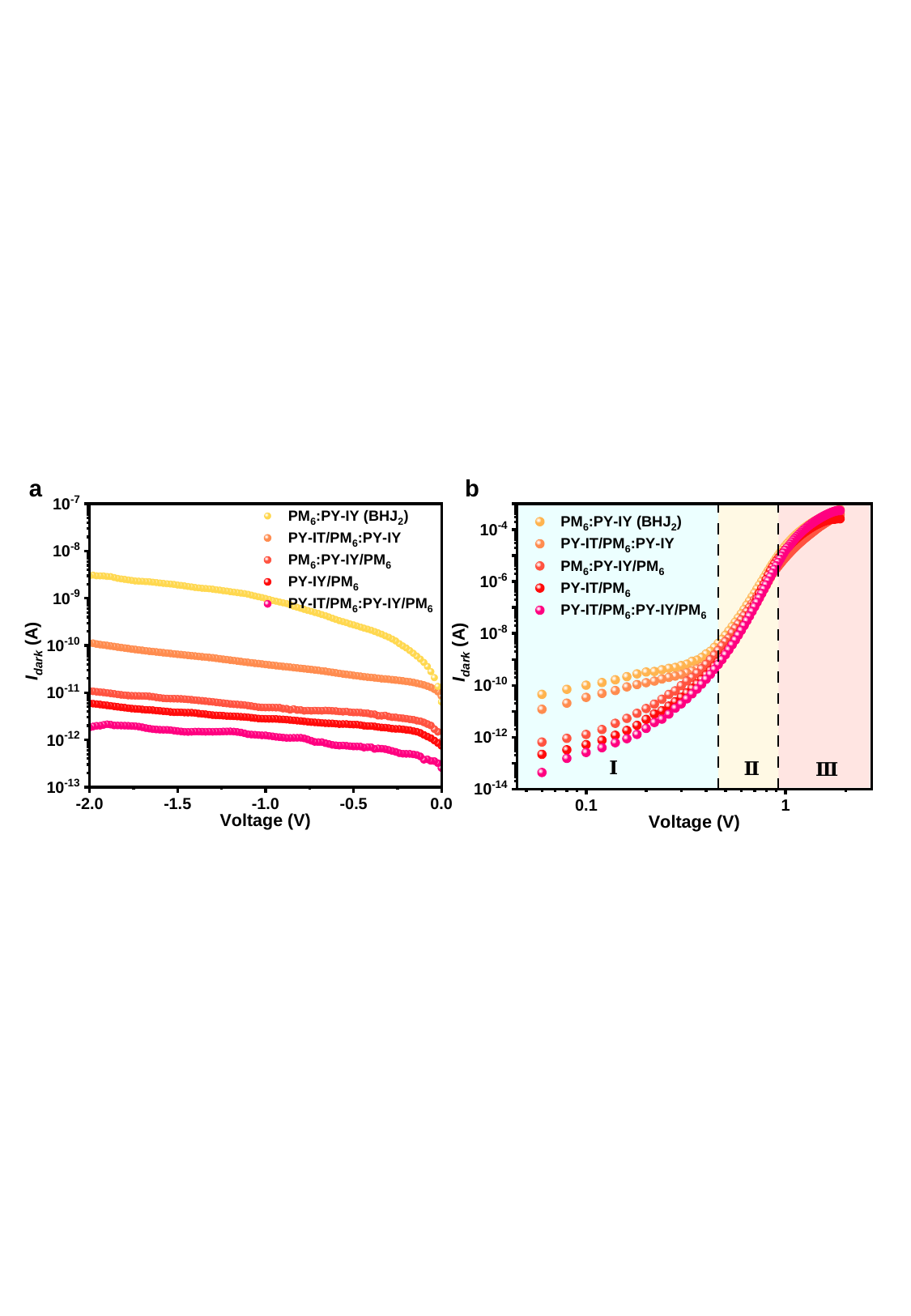

a
b

## Slide 15
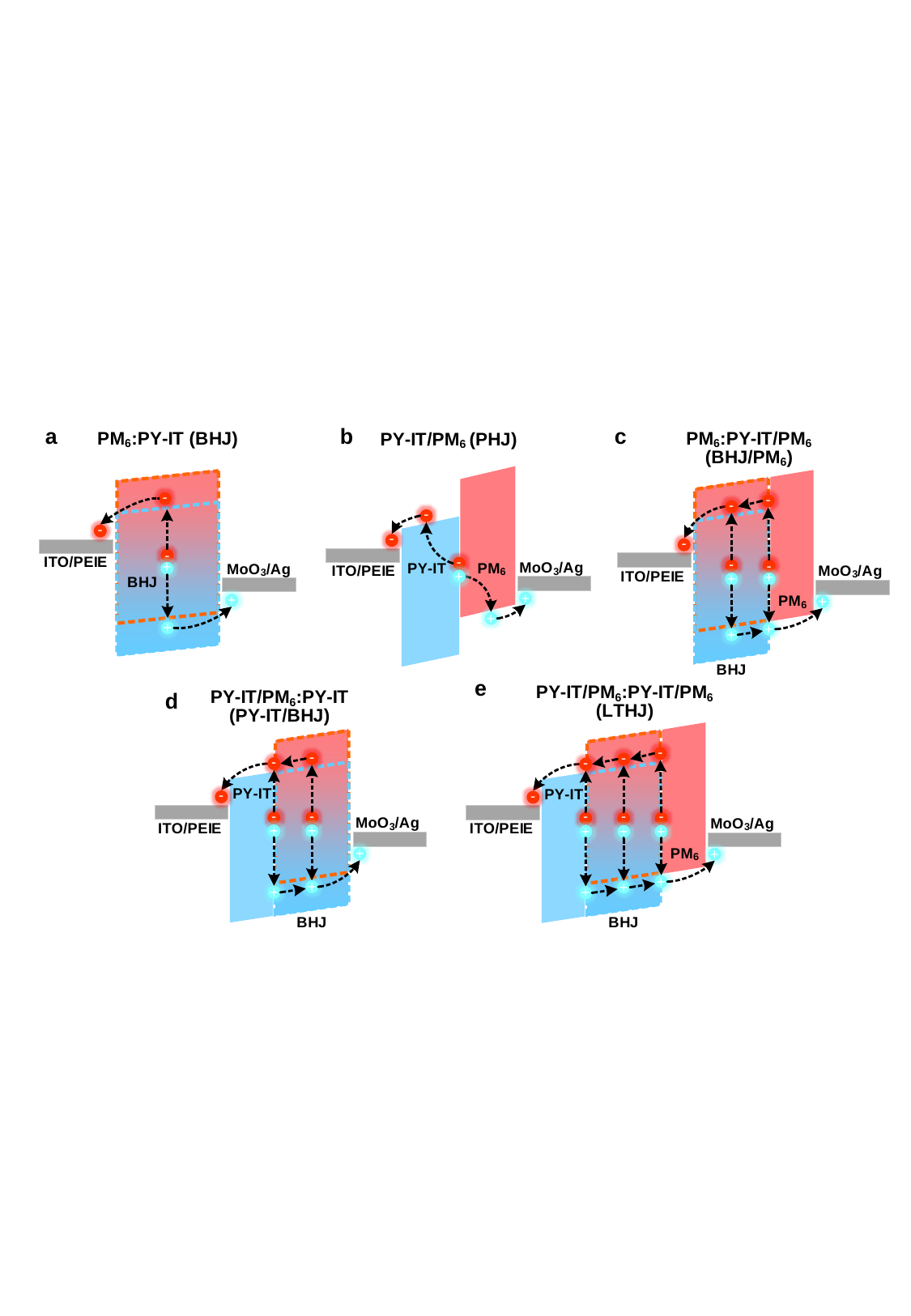

## Slide 16
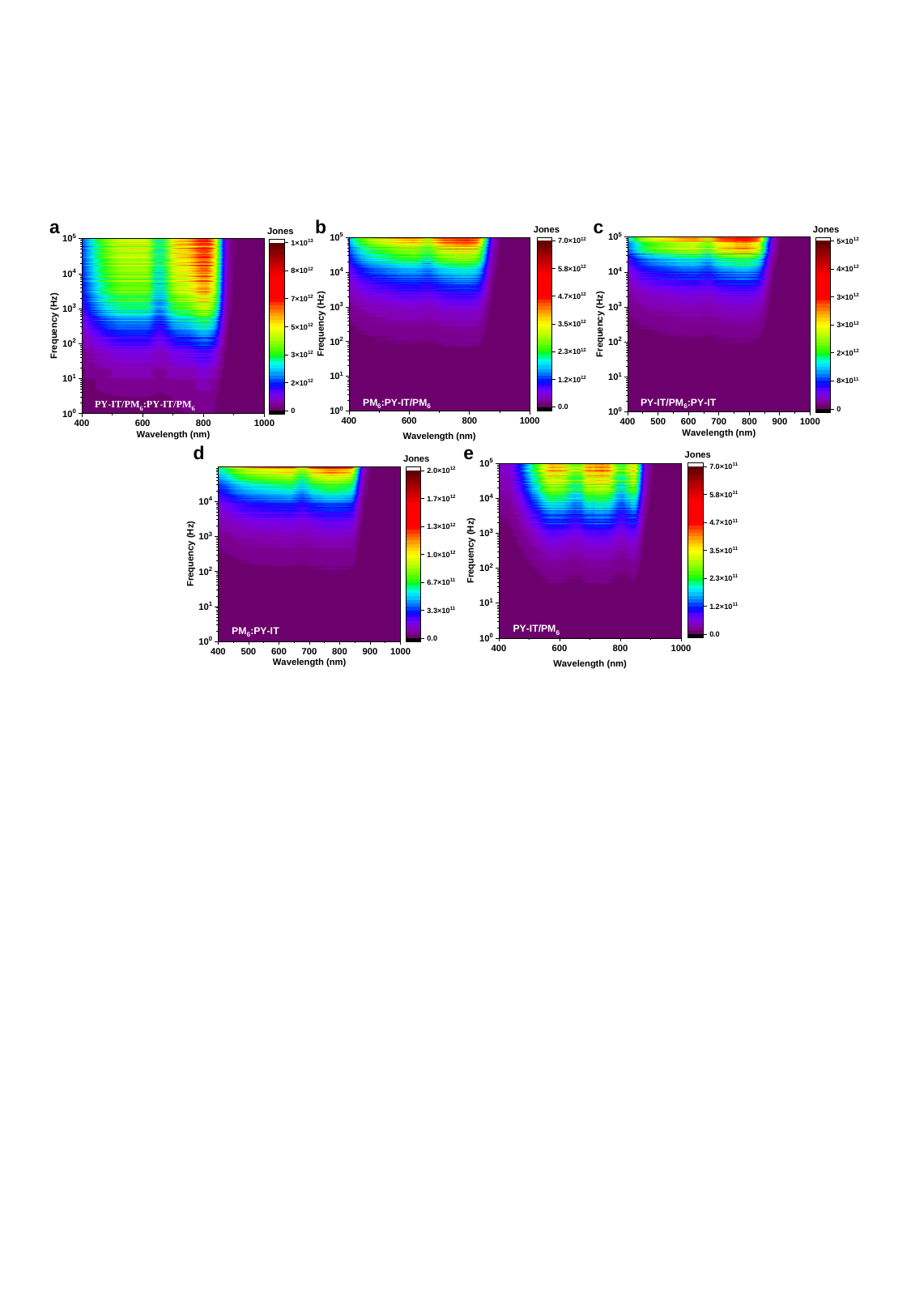

a
b
c
d
e

## Slide 17
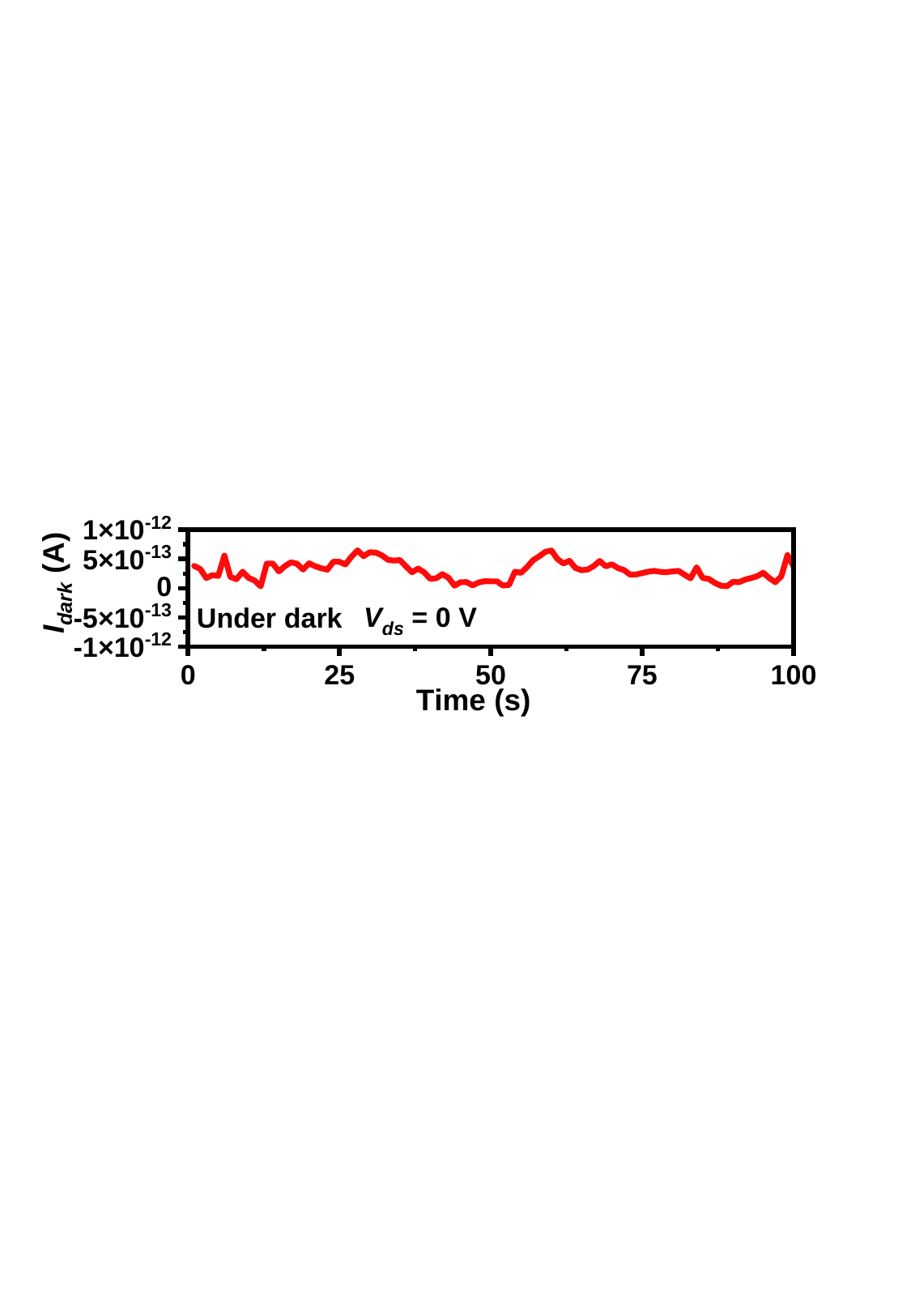

## Slide 18
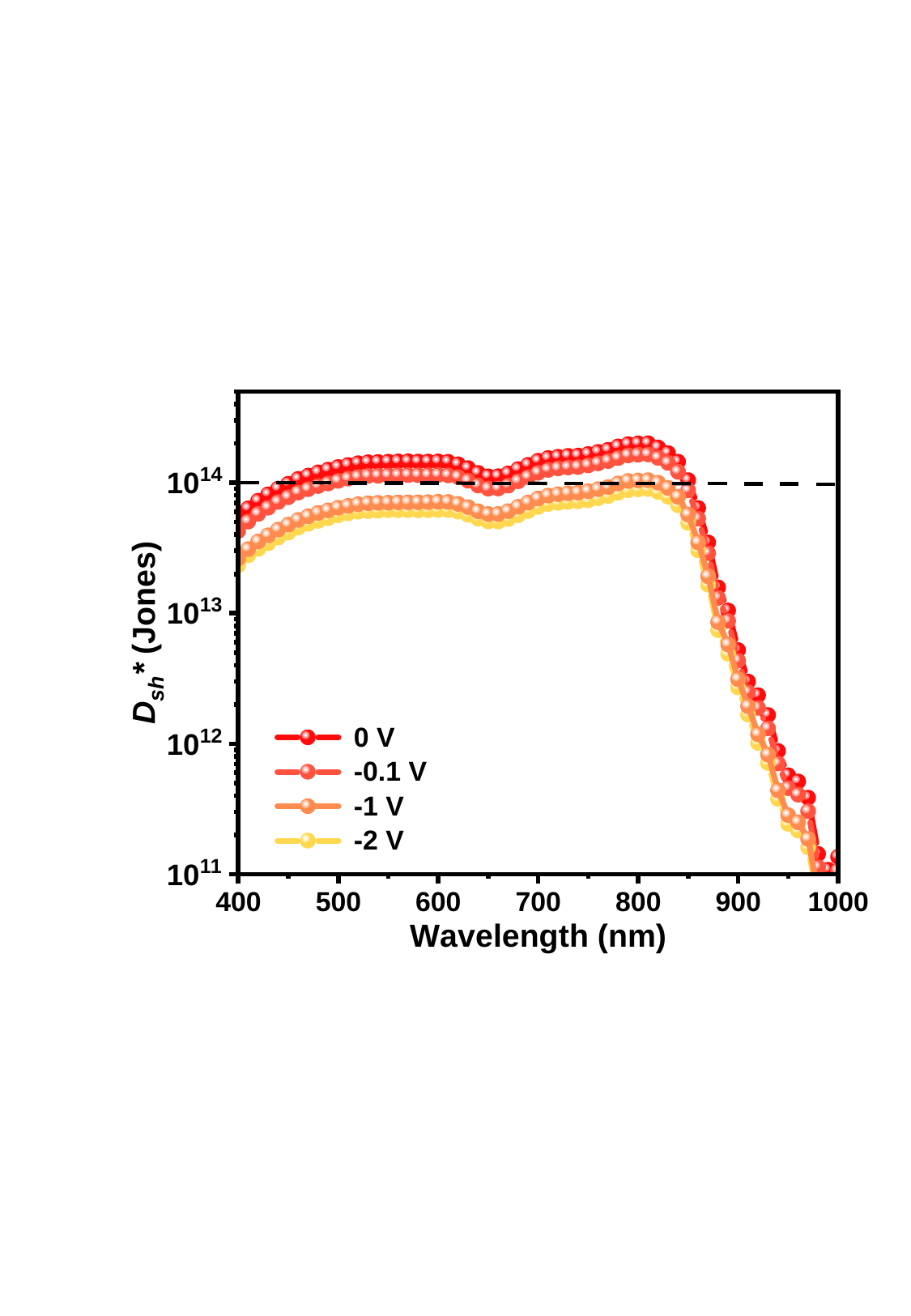

## Slide 19
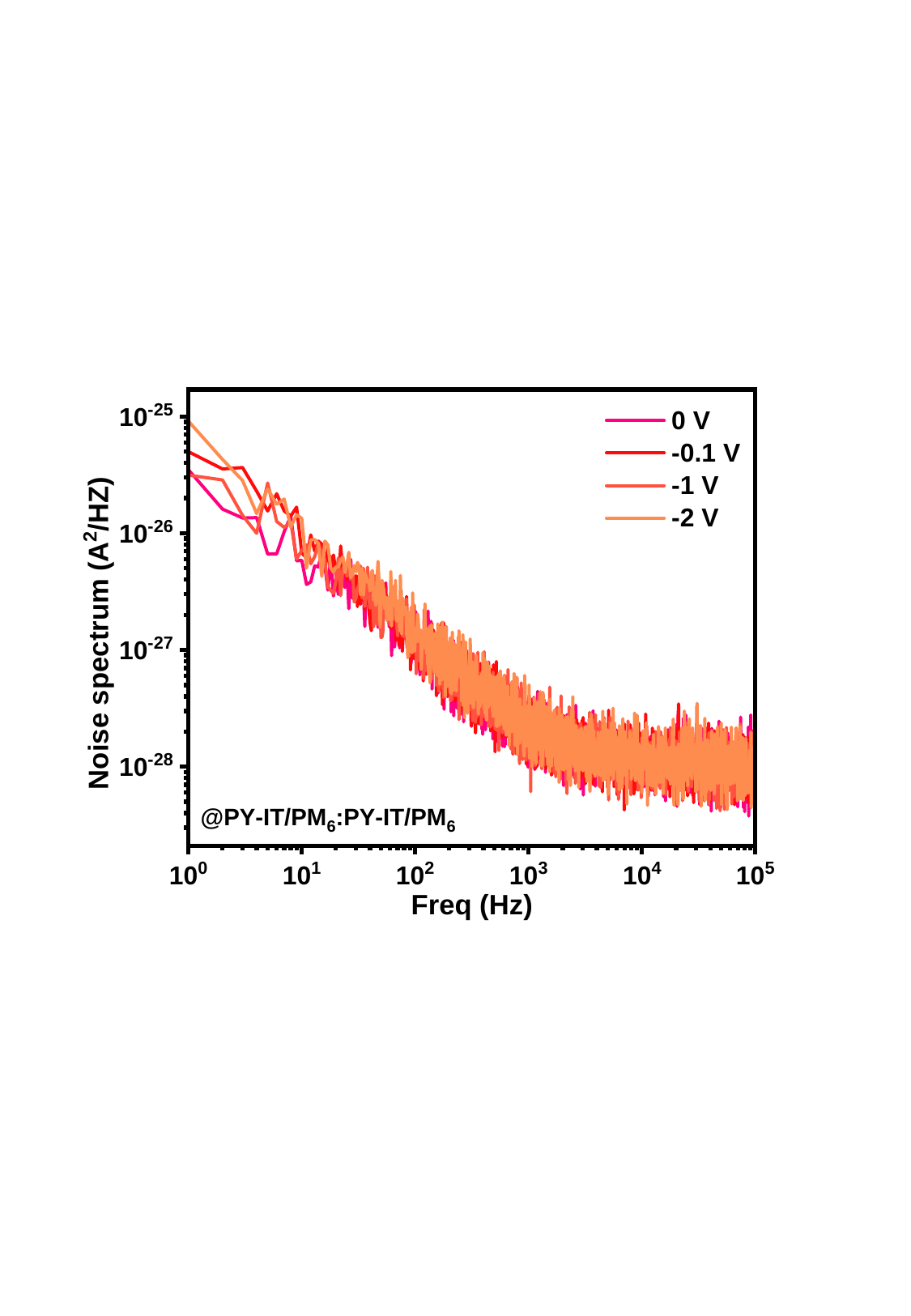

## Slide 20
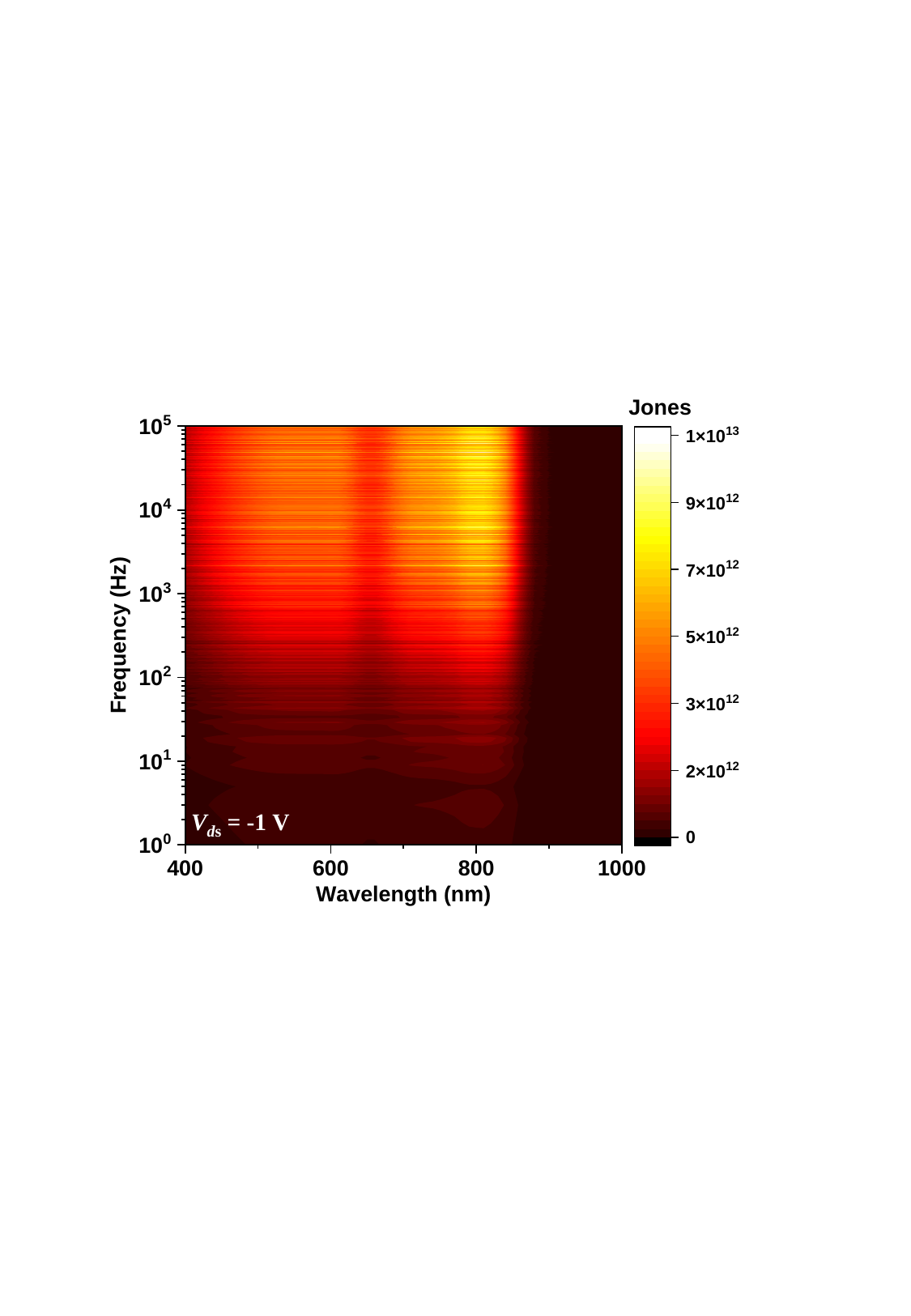

## Slide 21
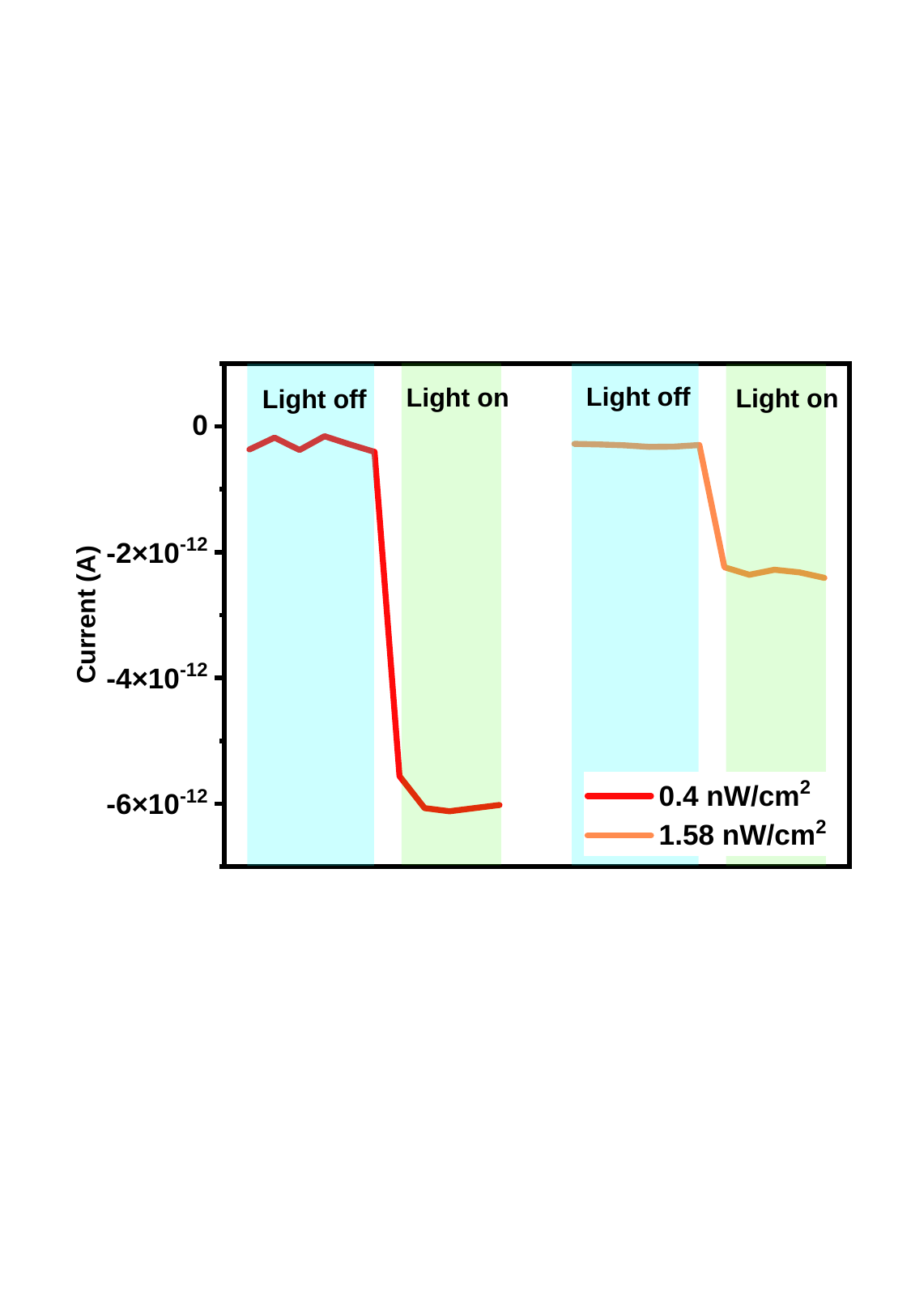

## Slide 22
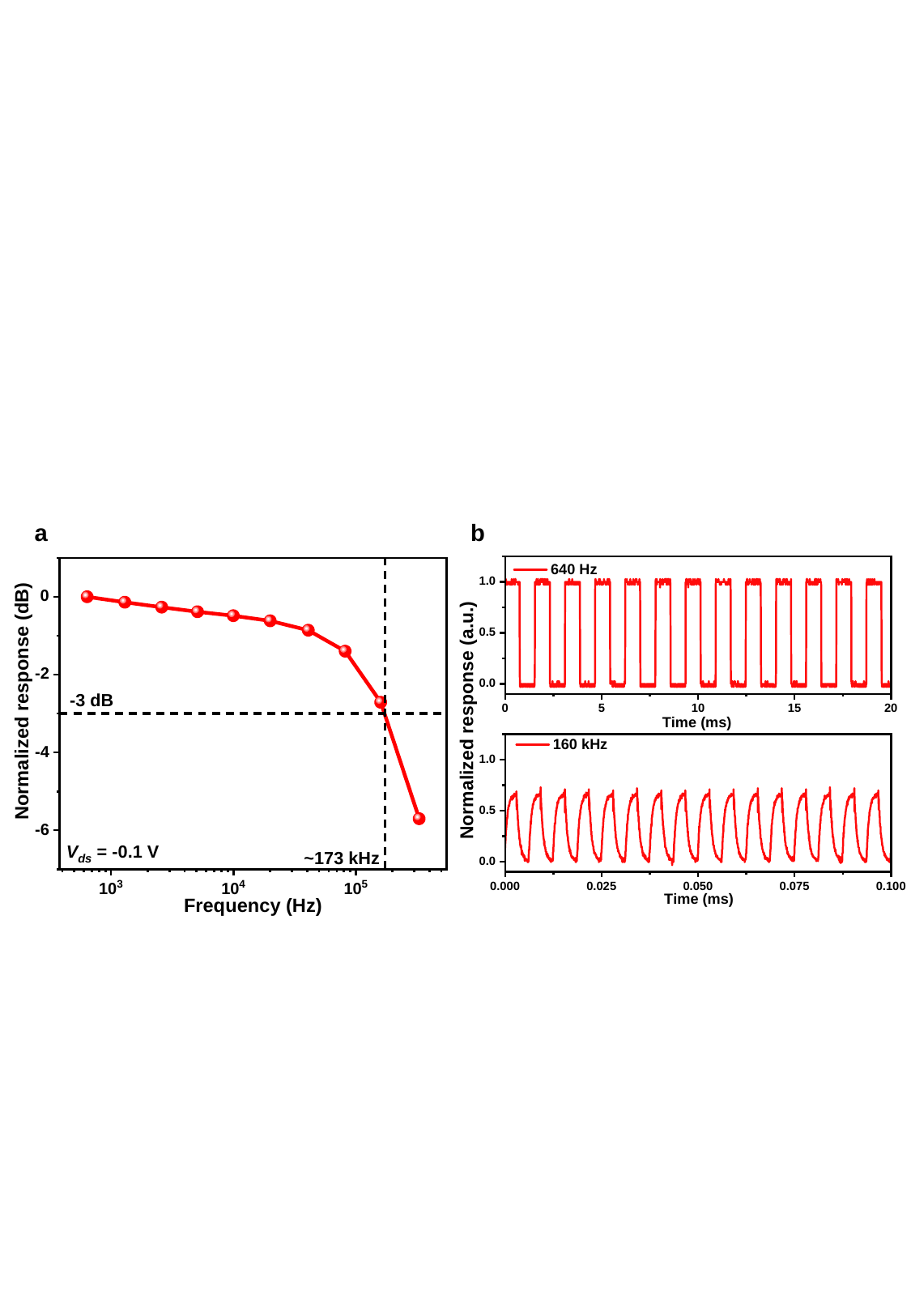

a
b

## Slide 23
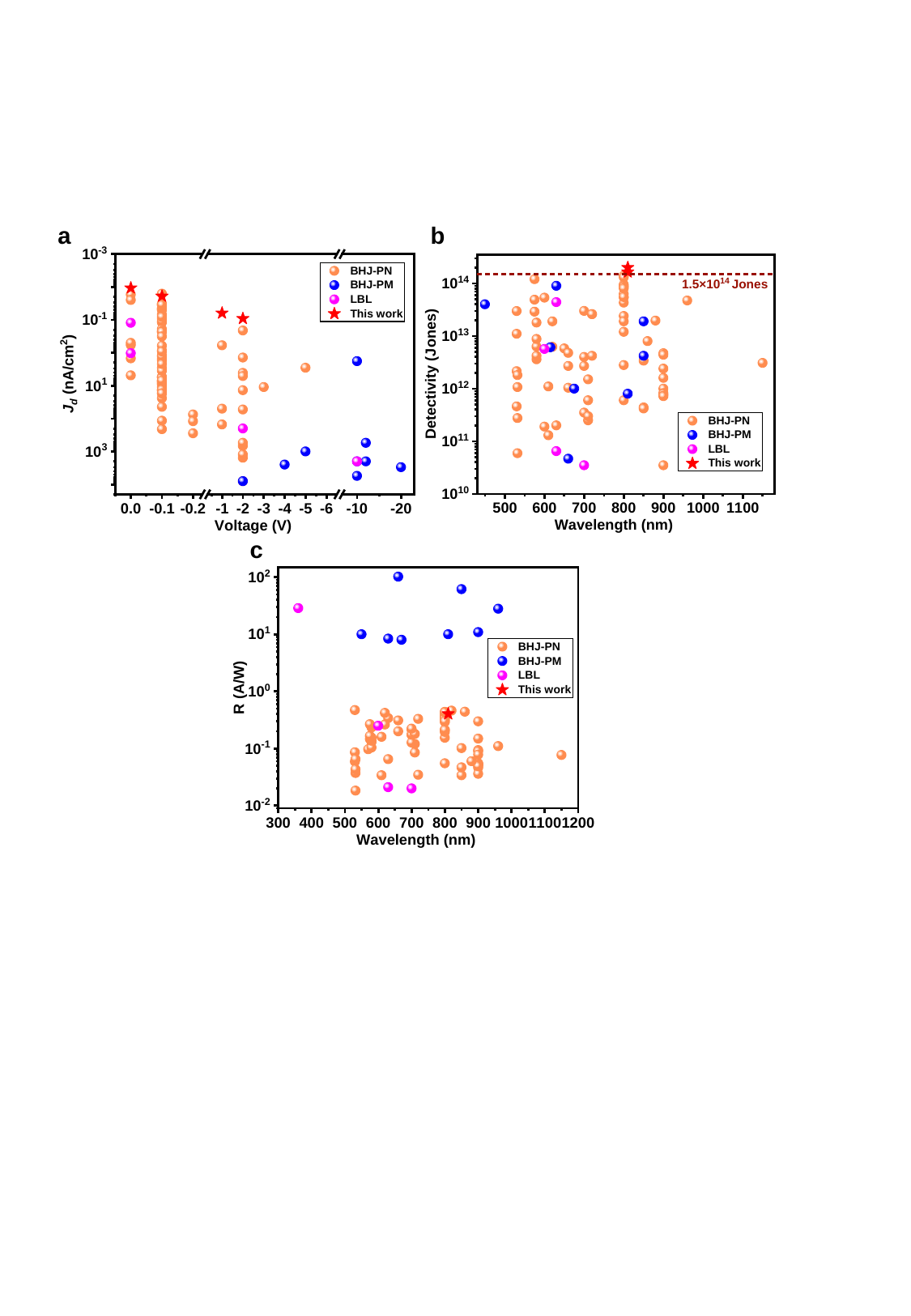

a
b
c

## Slide 24
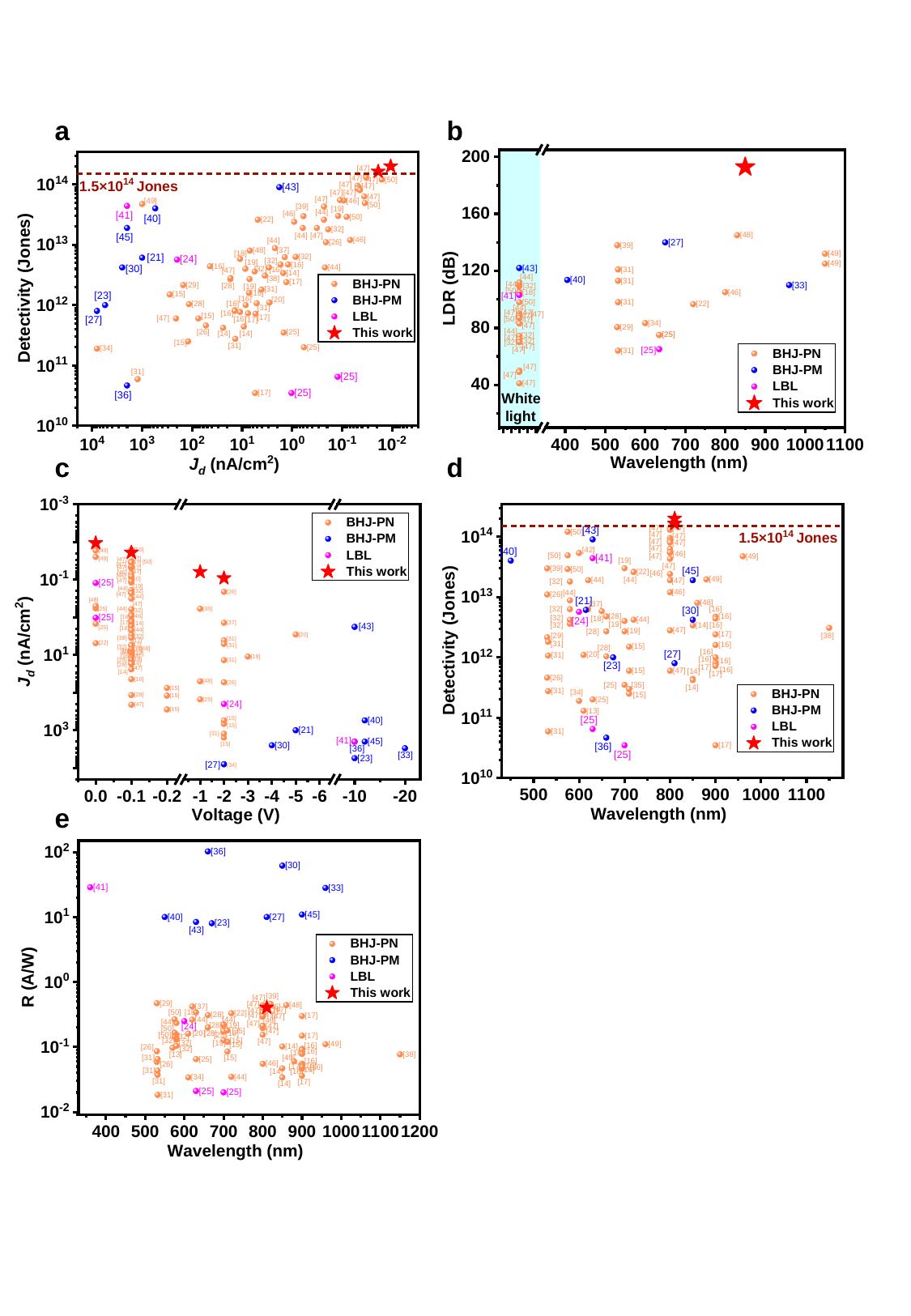

a
b
c
d
e

## Slide 25
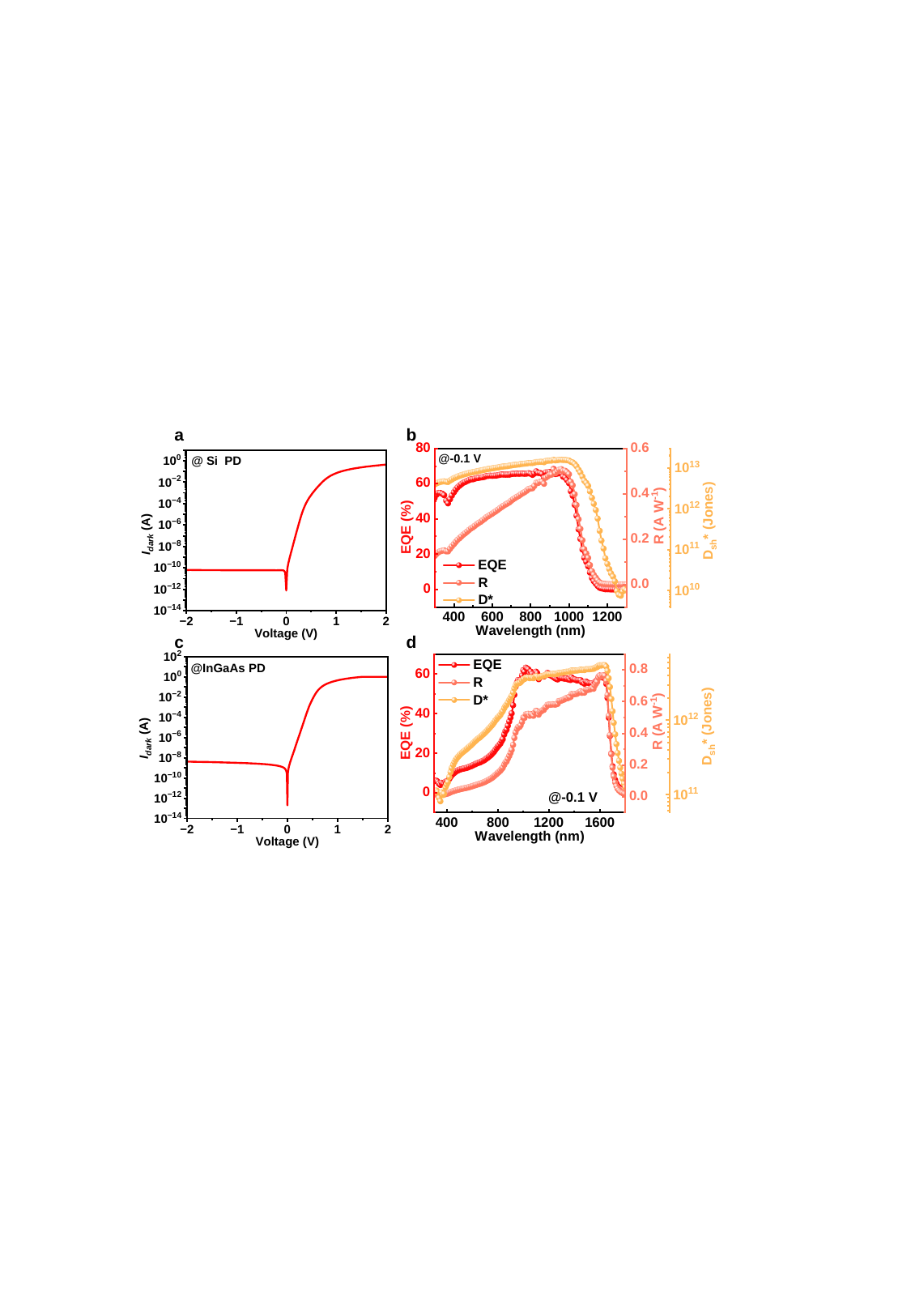

a
b
c
d
