# Supplementary material for: Ultra-Sensitive All-Polymer Near-Infrared Photodetectors via Van der Waals Layered Triple Heterojunction
Source: Research (Wash D C). 2025 Oct 3;8:0939. doi: 10.34133/research.0939 (PMC12491782; doi:10.34133/research.0939)
Supplement: Supplementary 1 — Texts S1 to S11 Tables S1 to S5 Figs. S1 to S42 References [56–105] [file research.0939.f1.zip › Figure-Supporting 2.pptx]

## Slide 1
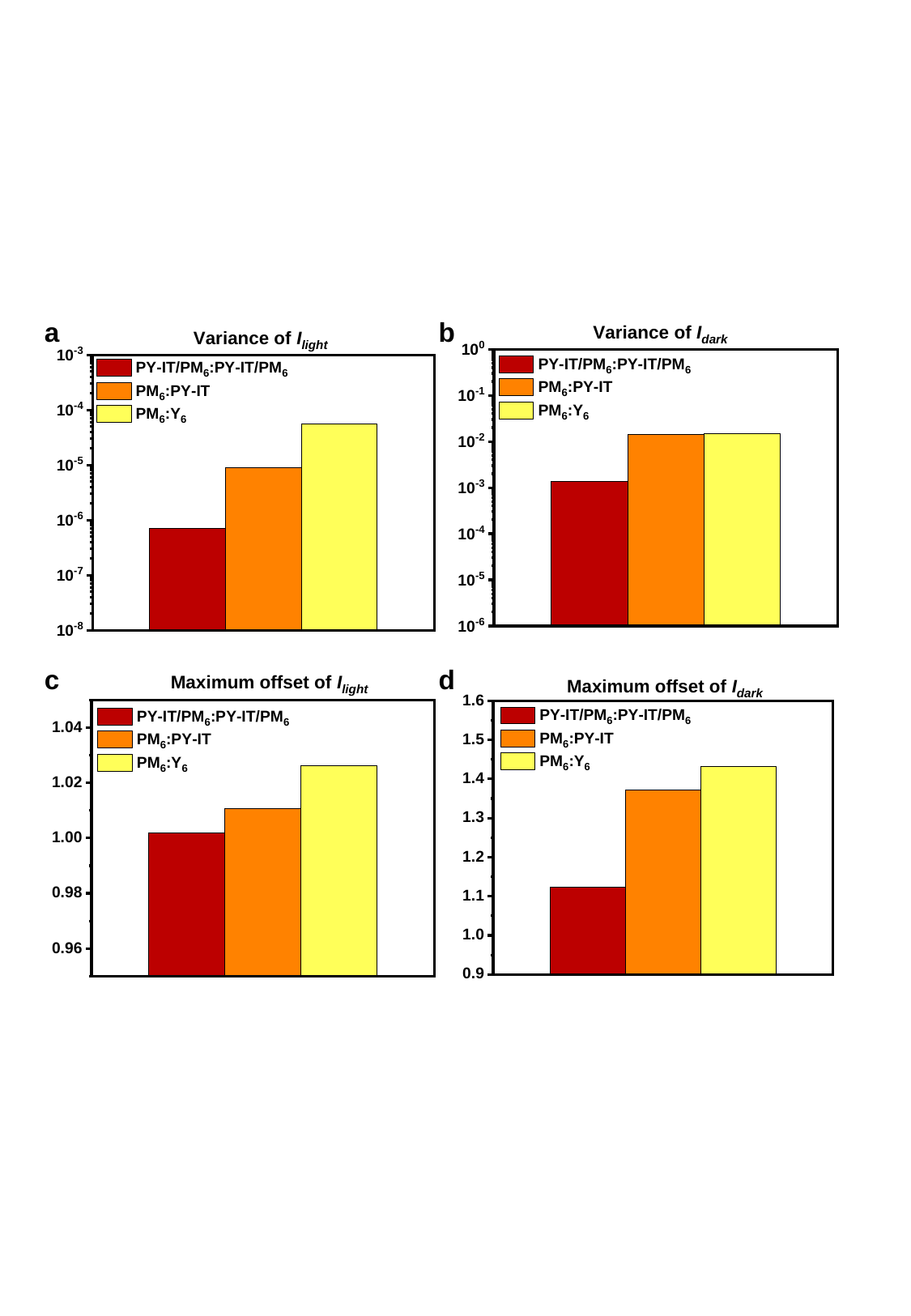

a
b
c
d

## Slide 2
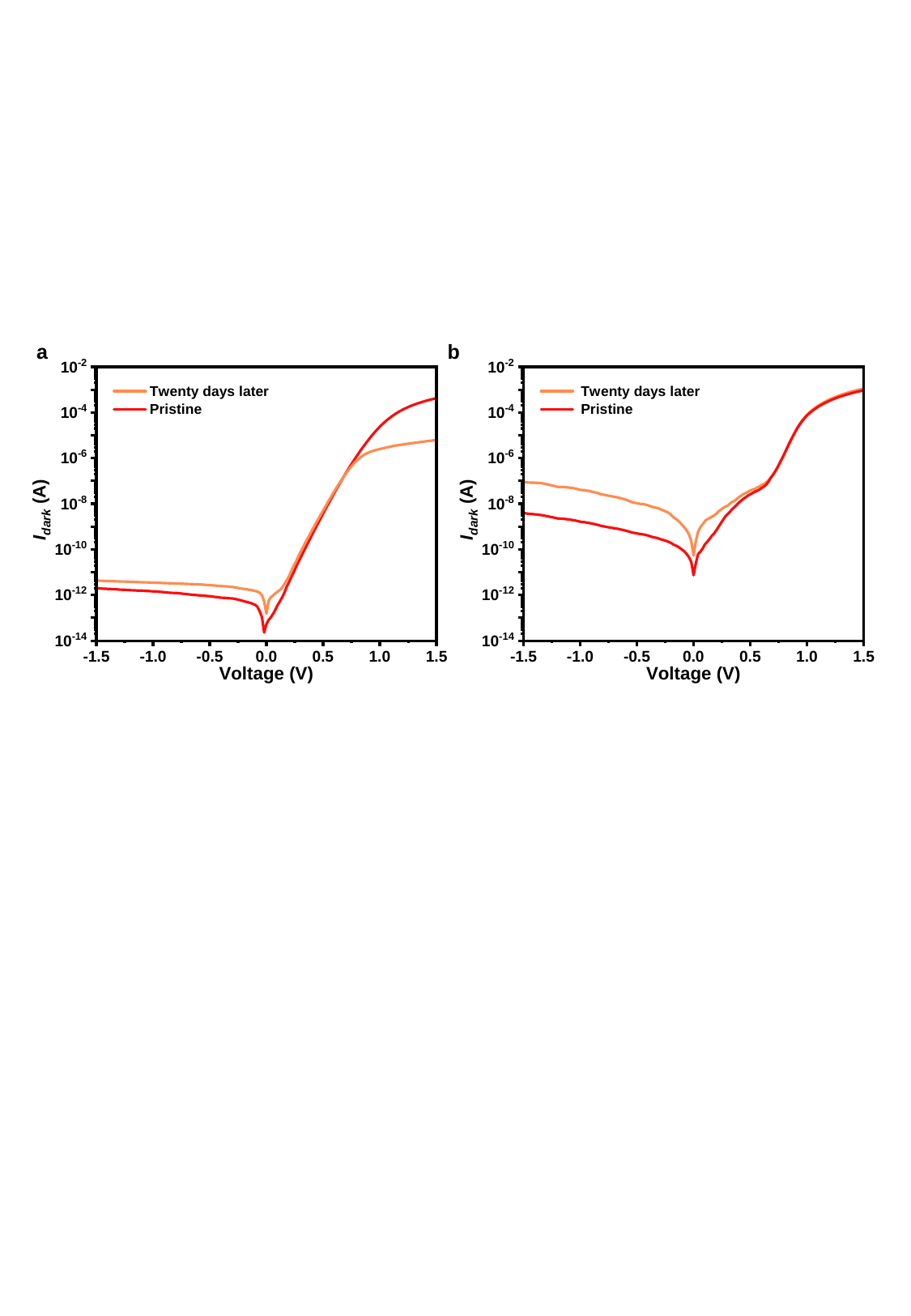

a
b

## Slide 3
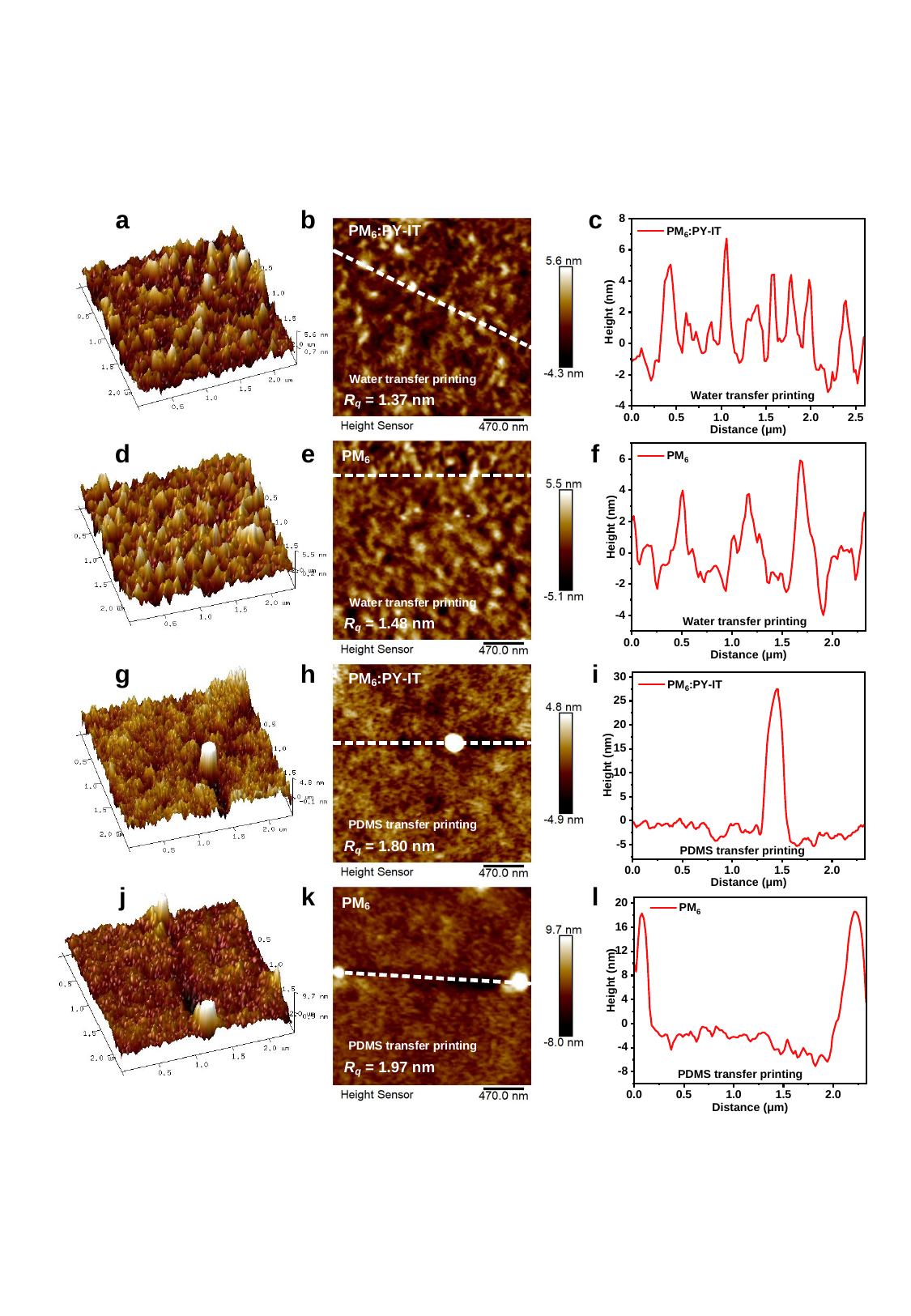

## Slide 4
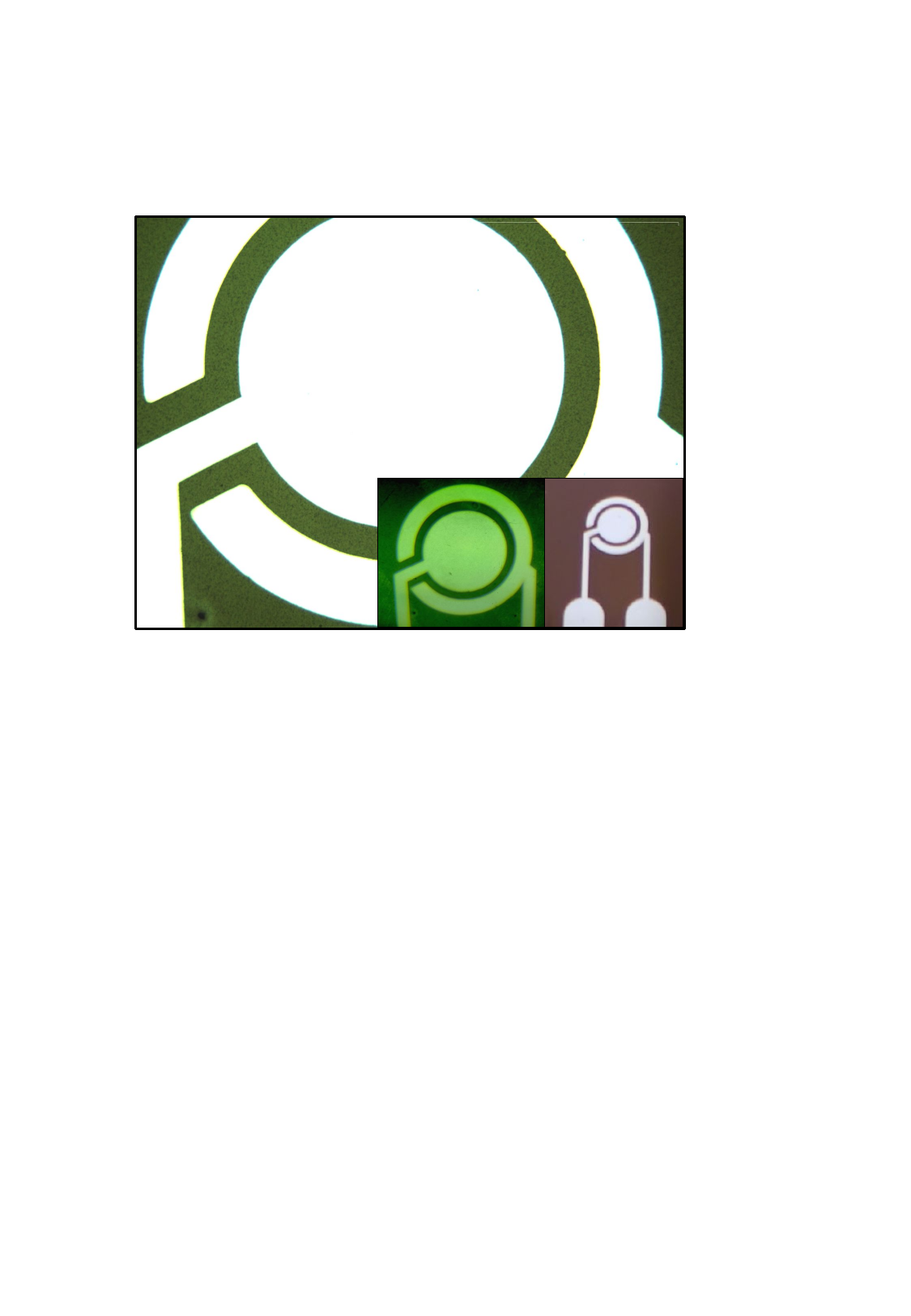

## Slide 5
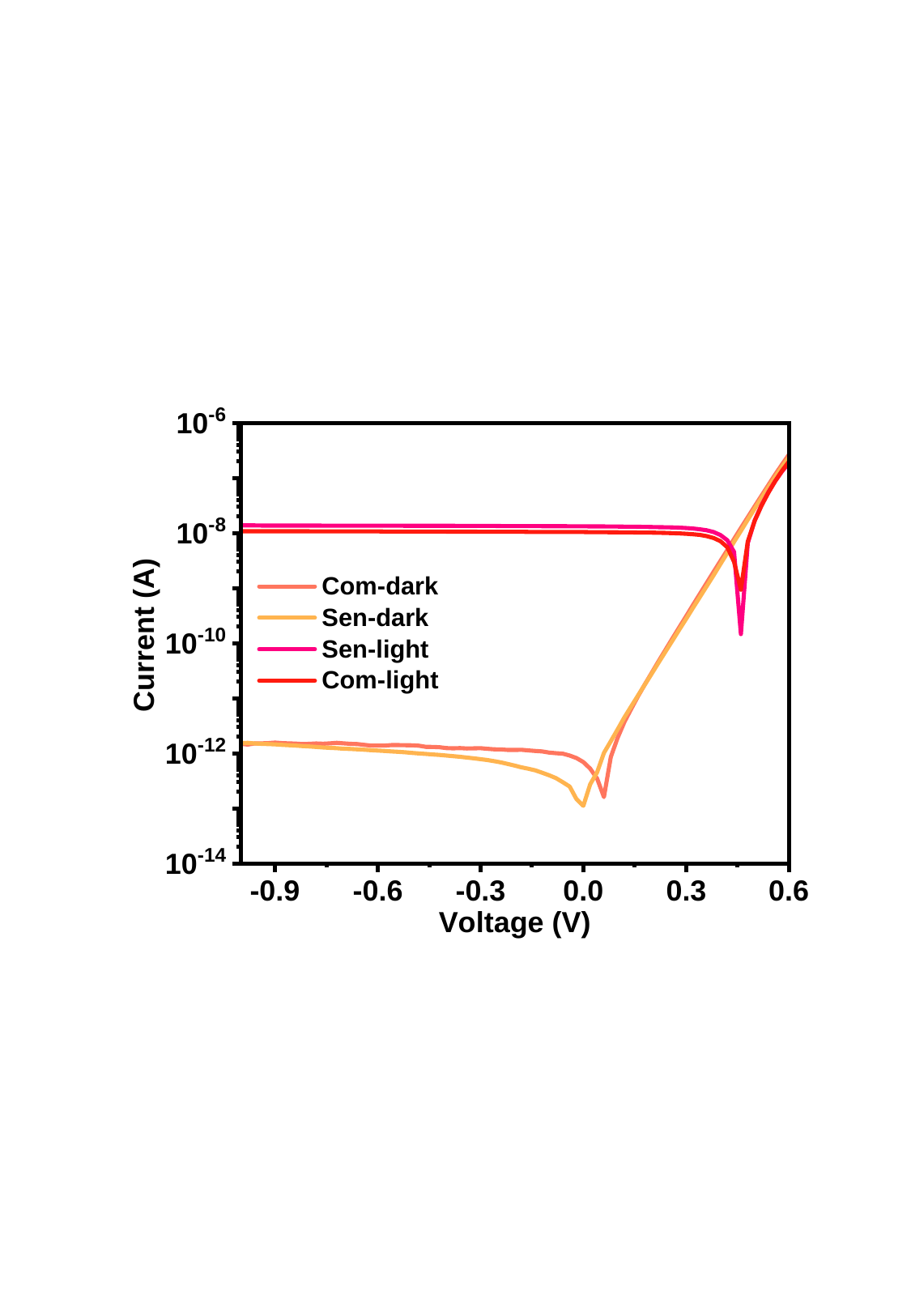

## Slide 6
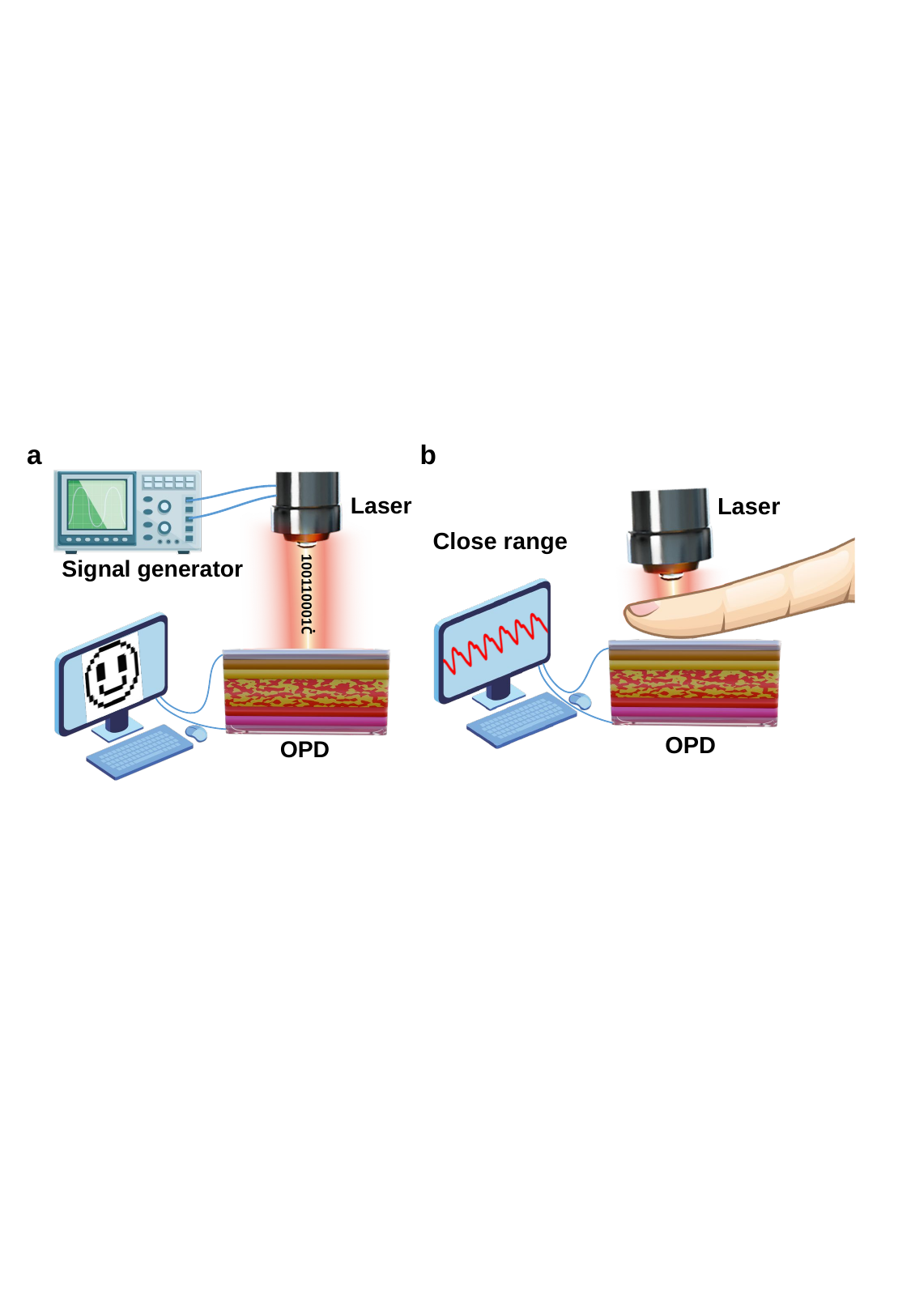

a
b

## Slide 7
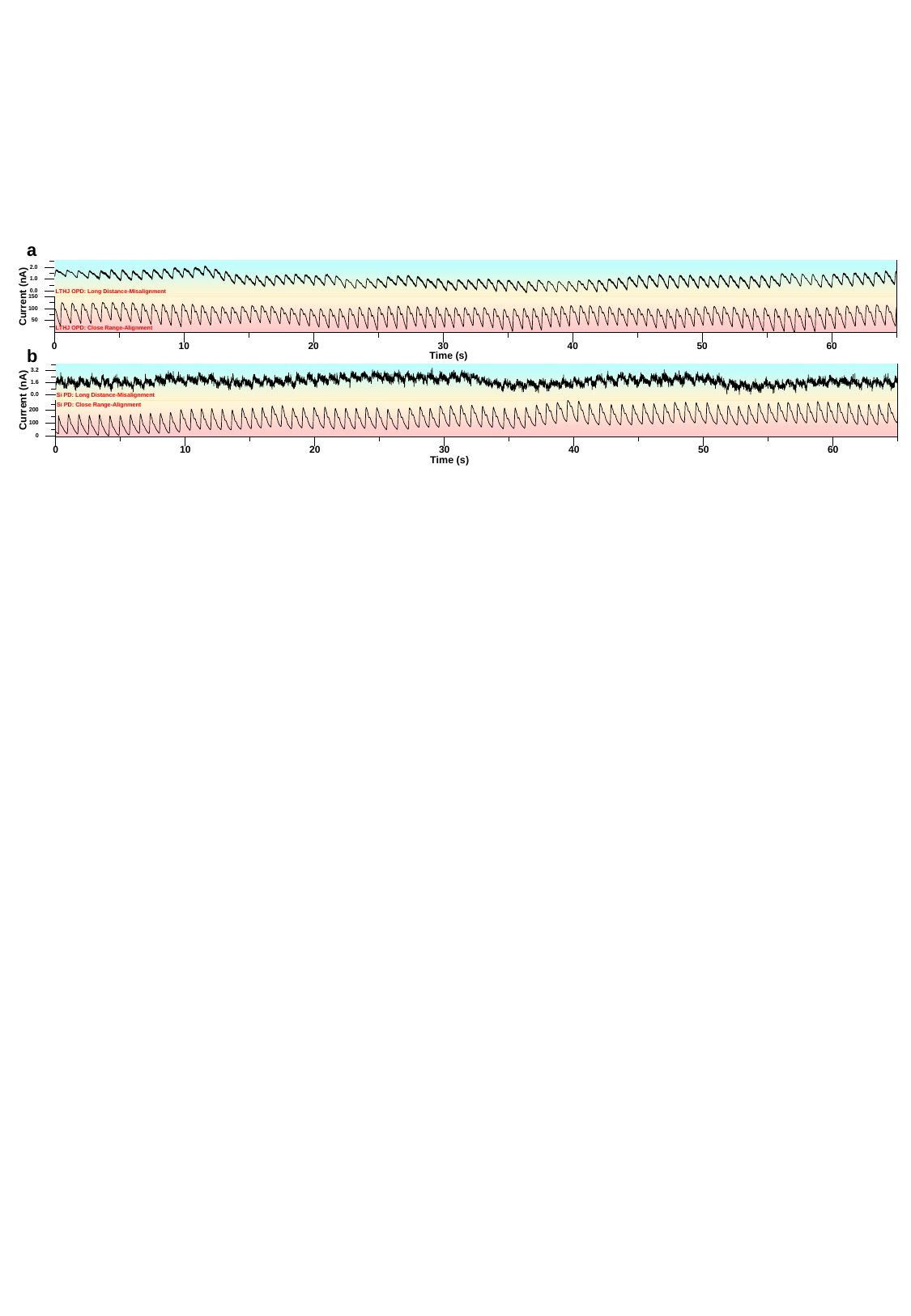

a
b

## Slide 8
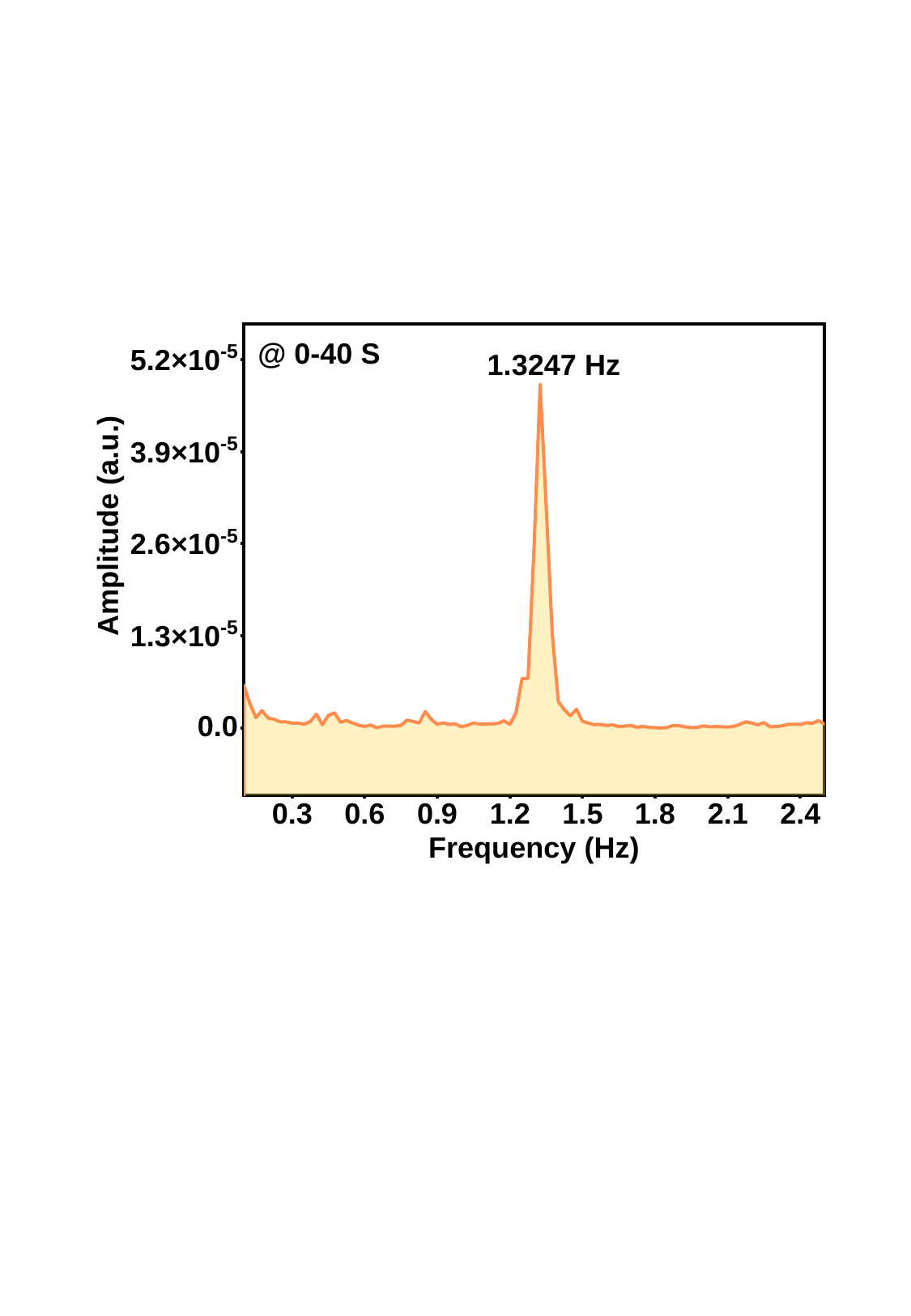

## Slide 9
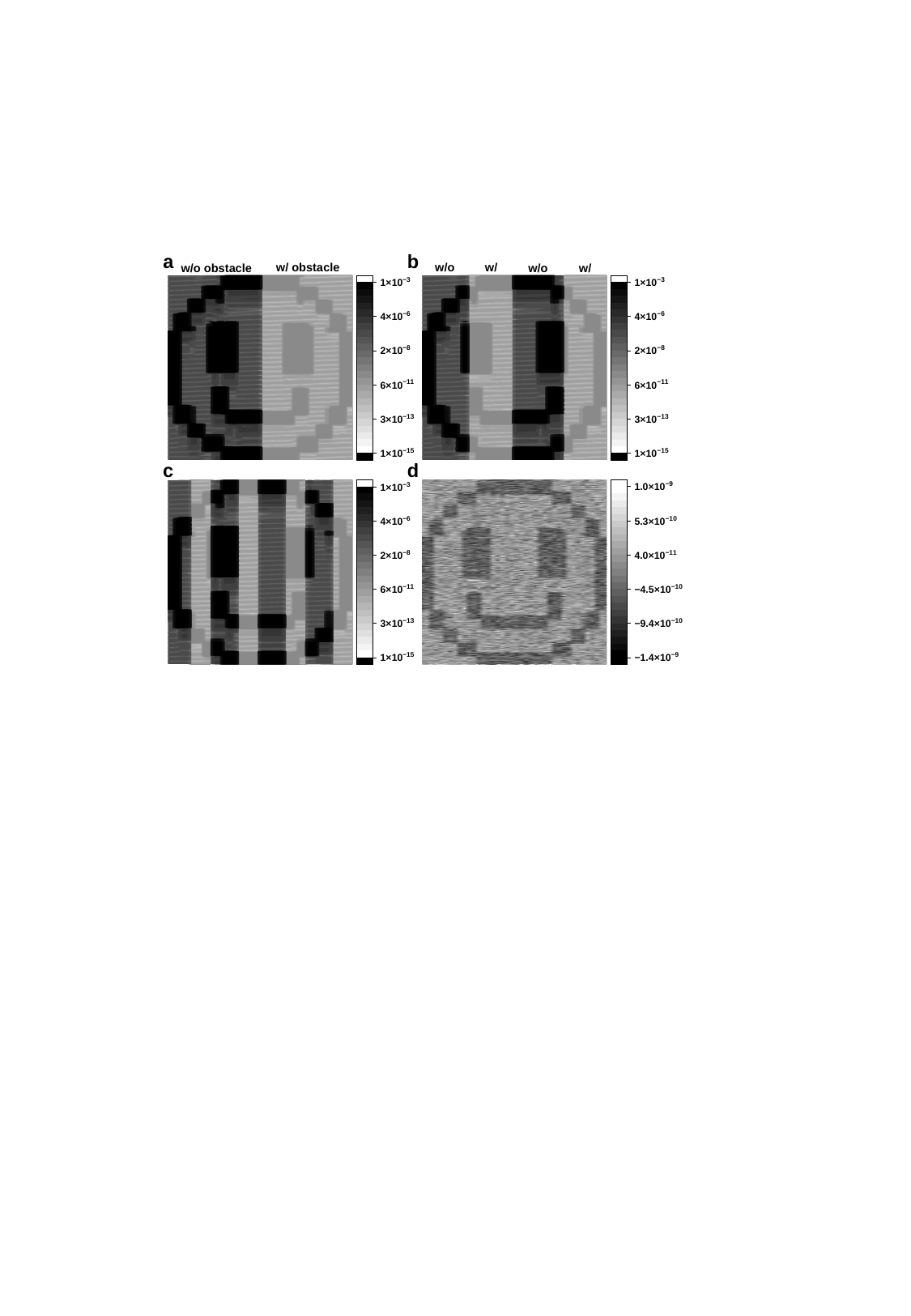

a
b
c
d

## Slide 10
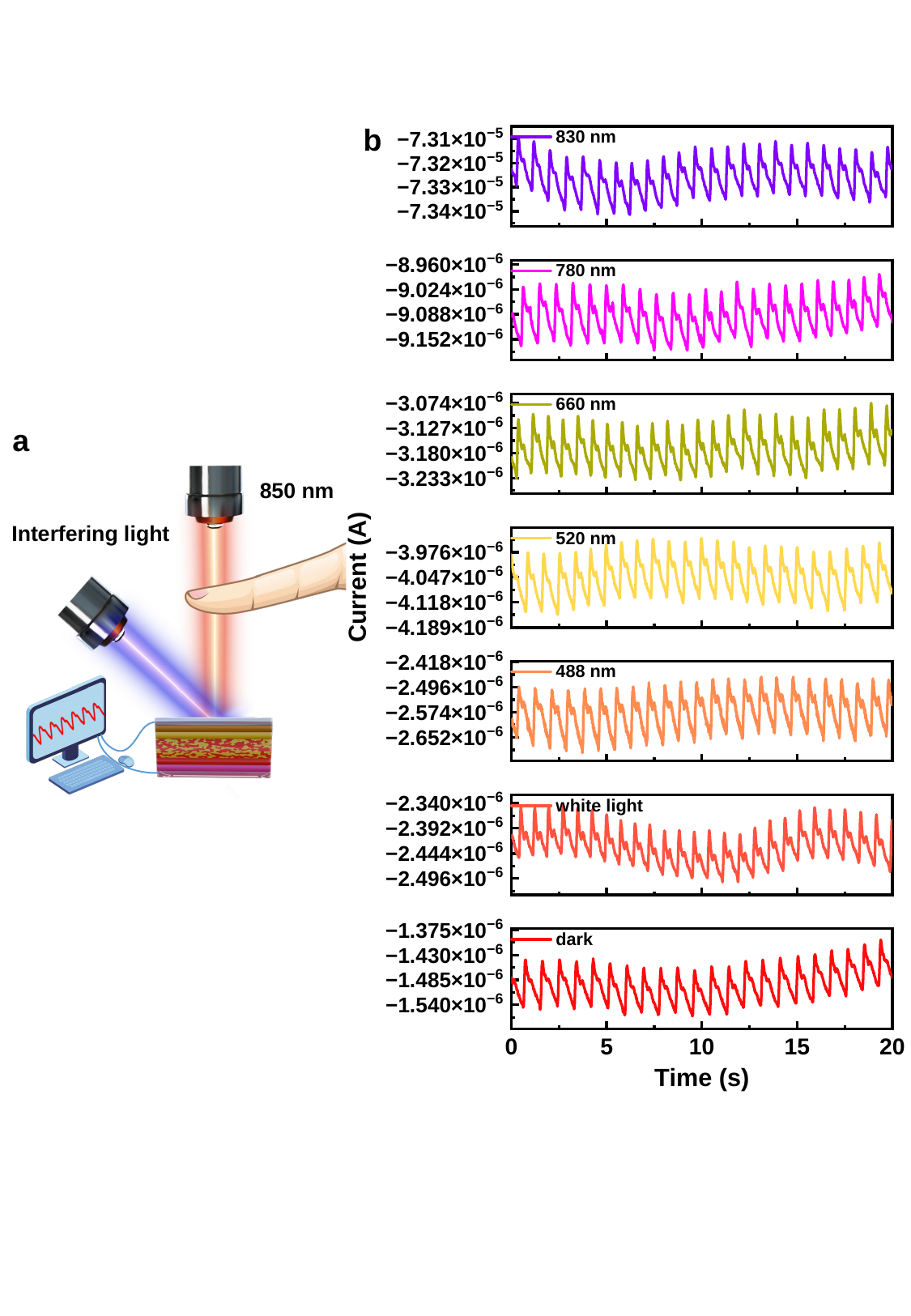

b
a

## Slide 11
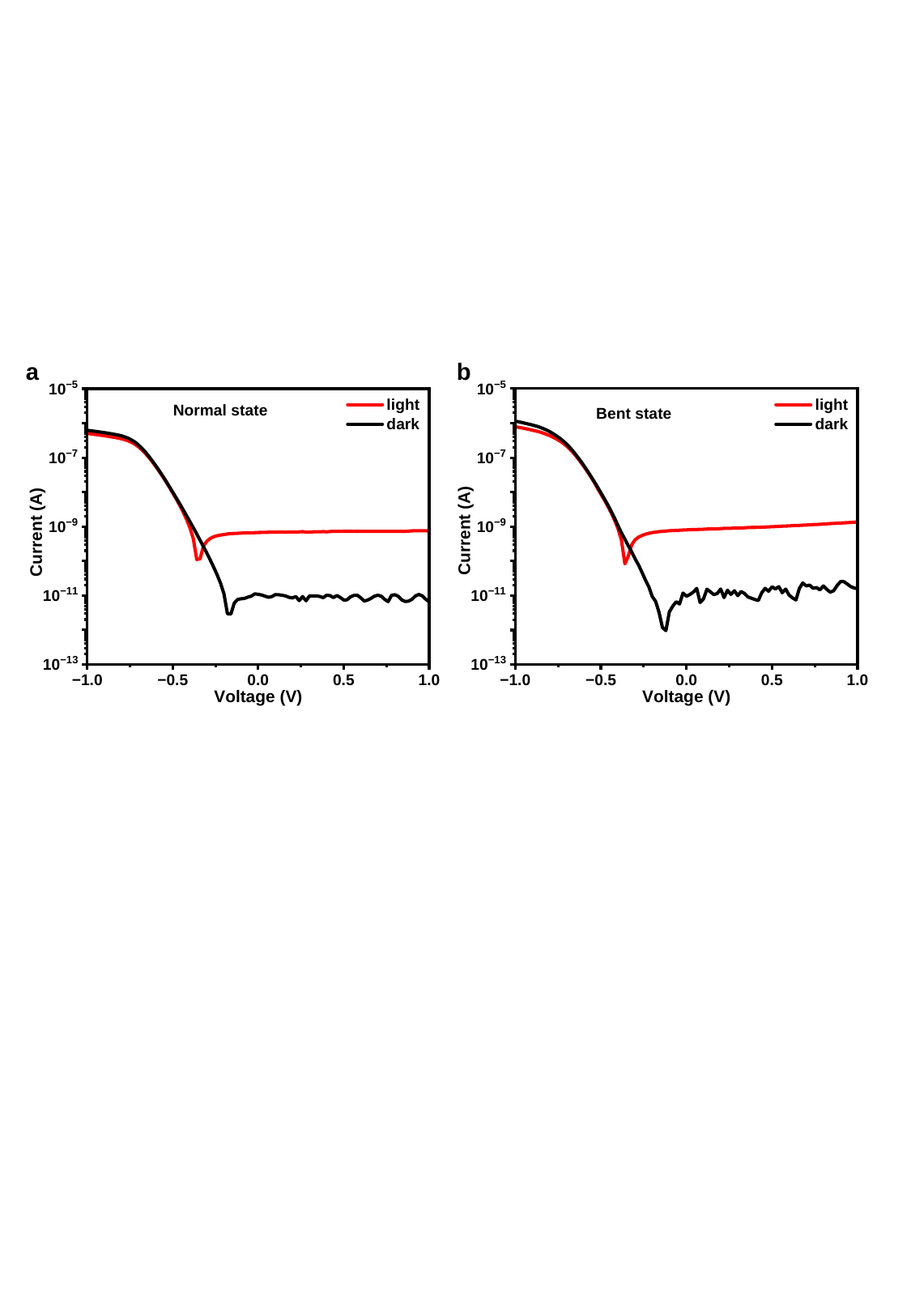

a
b

## Slide 12
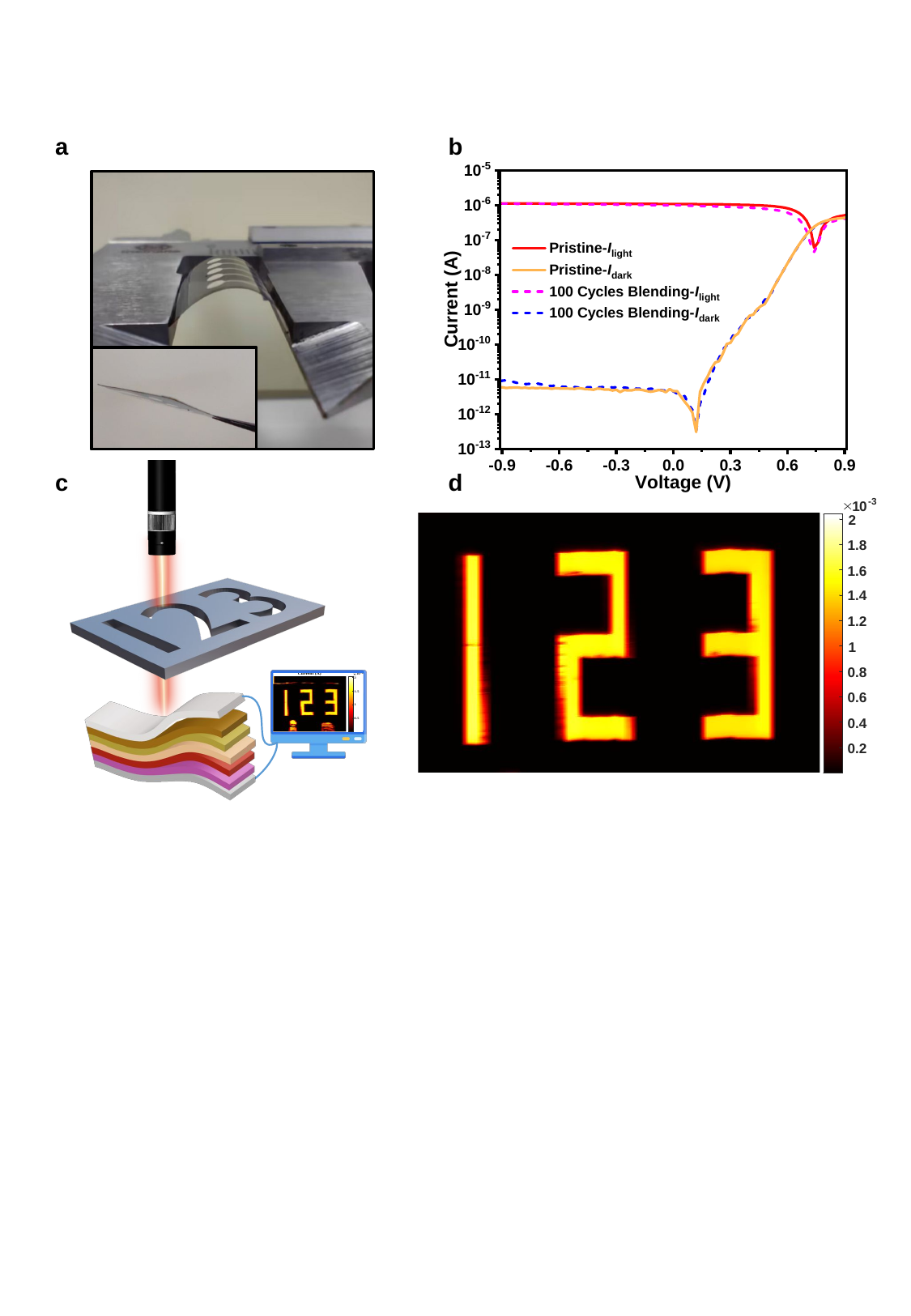

a
b
c
d

## Slide 13
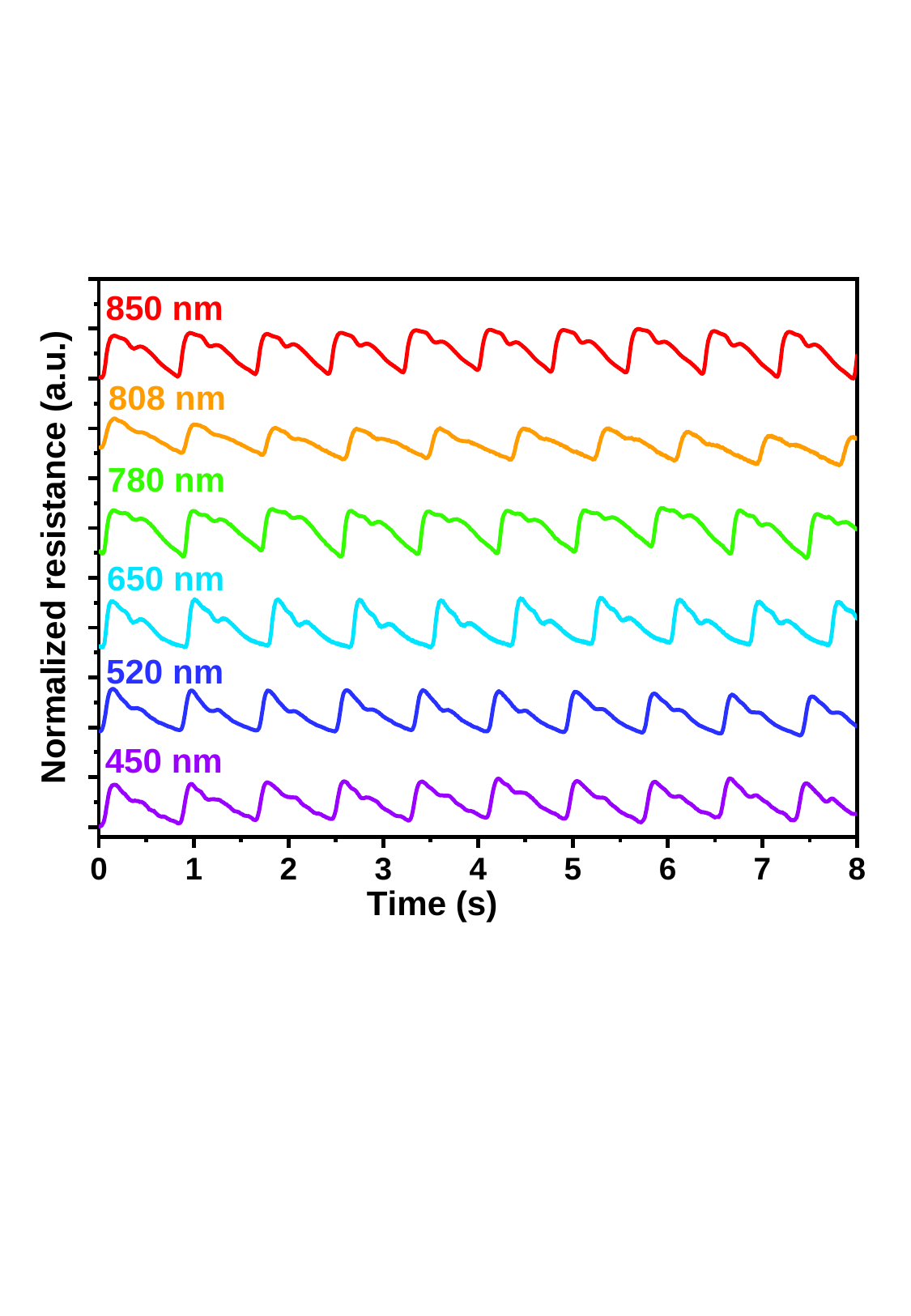

## Slide 14
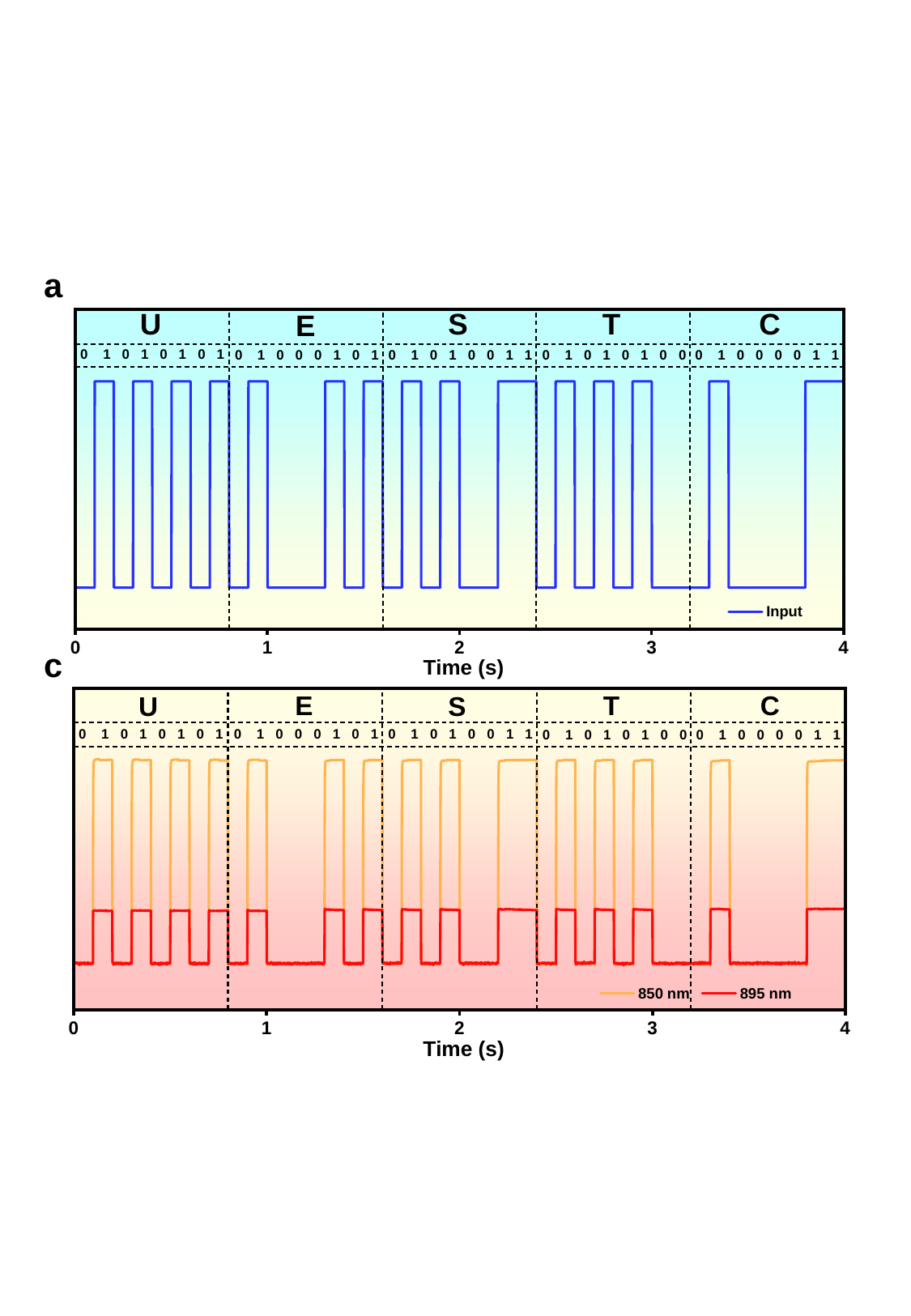

a
c

## Slide 15
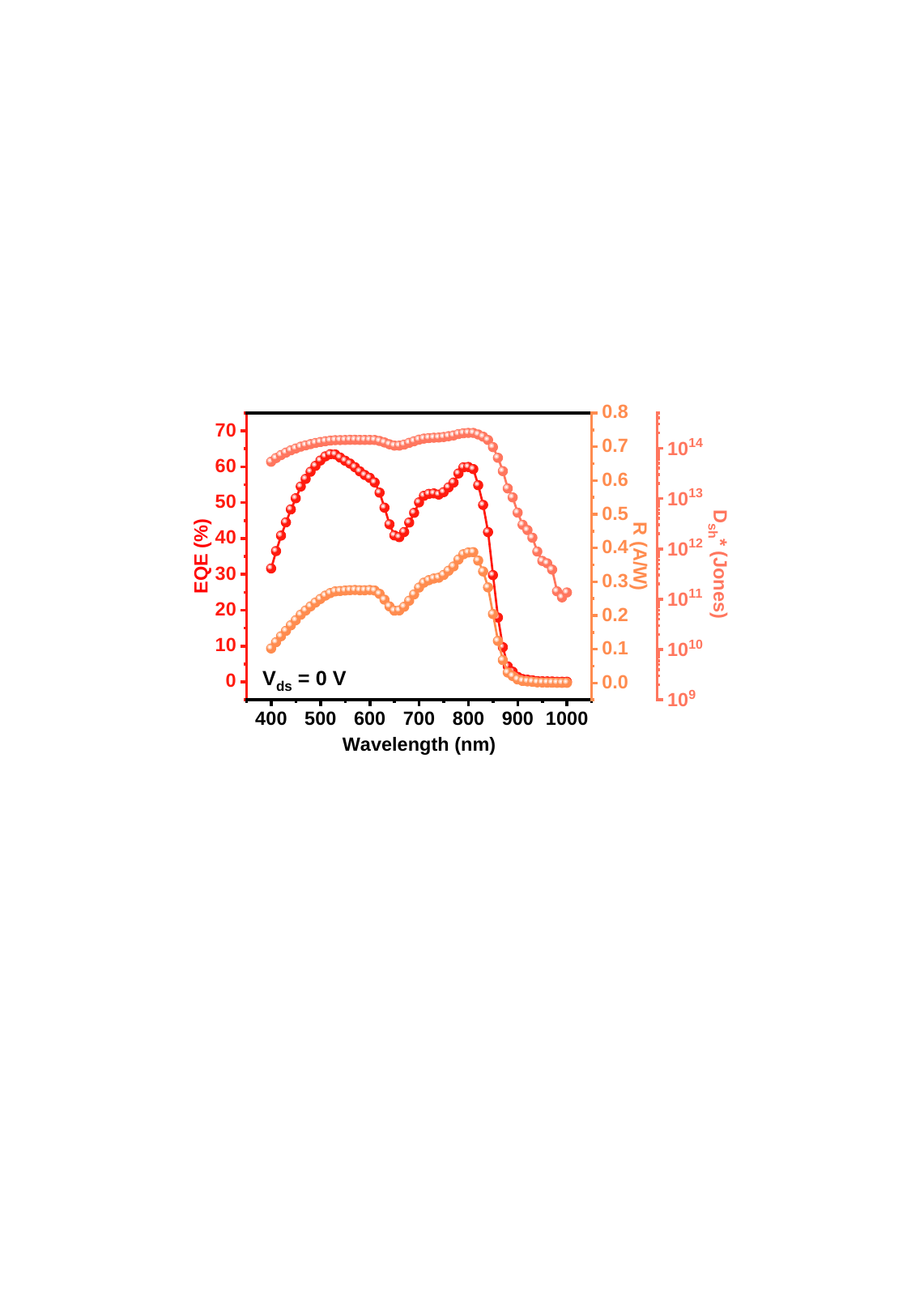

## Slide 16
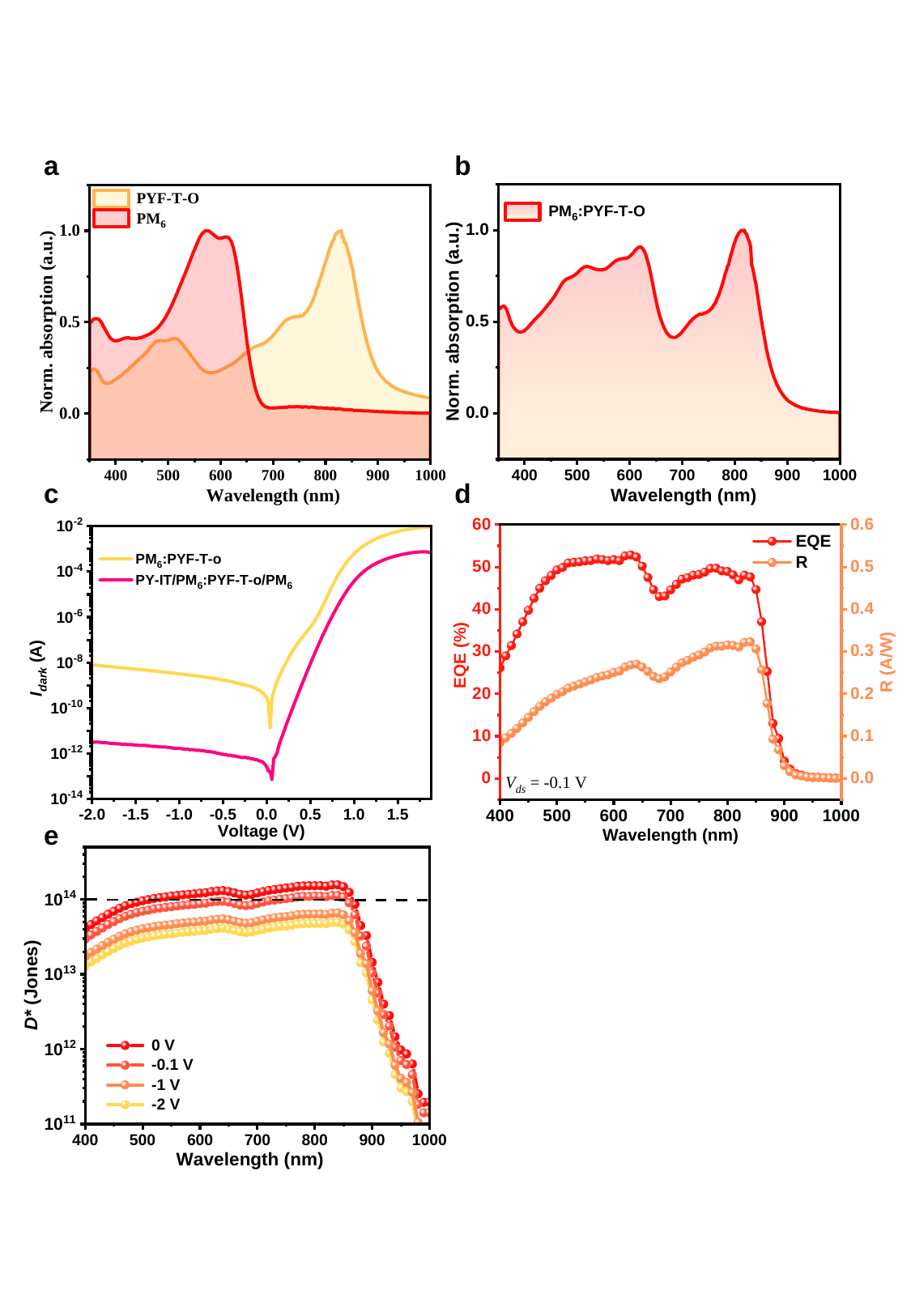

a
b
c
d
e

## Slide 17
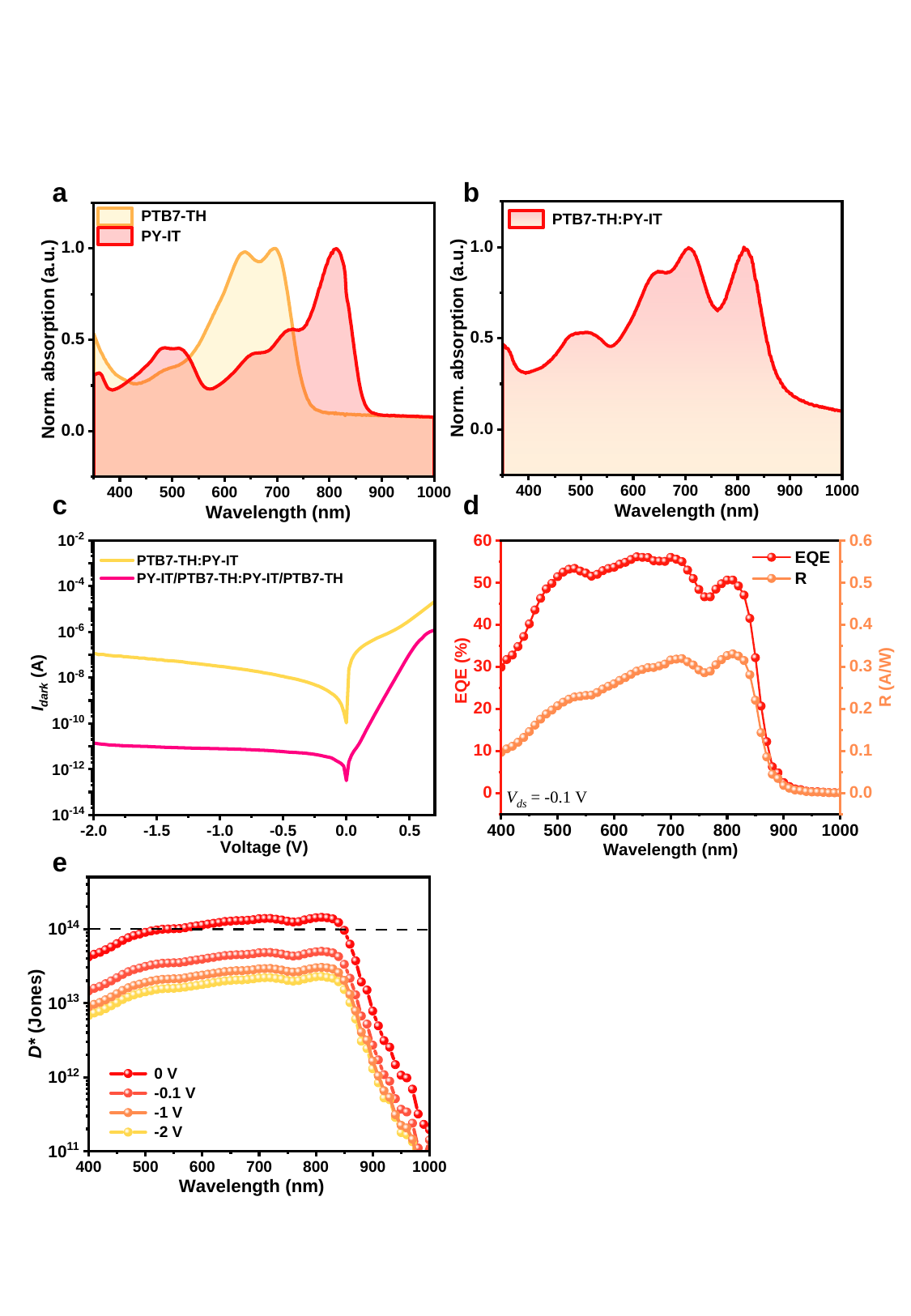

a
b
c
d
e
