# Supplementary material for: Ultra-Sensitive All-Polymer Near-Infrared Photodetectors via Van der Waals Layered Triple Heterojunction
Source: Research (Wash D C). 2025 Oct 3;8:0939. doi: 10.34133/research.0939 (PMC12491782; doi:10.34133/research.0939)
Supplement: Supplementary 1 — Texts S1 to S11 Tables S1 to S5 Figs. S1 to S42 References [56–105] [file research.0939.f1.zip › Supporting Information-revised.docx]

**Supporting Information**

**Ultra-Sensitive All-Polymer NIR Photodetectors via Van der Waals Layered Triple Heterojunction**

Lei Guo1, Meiyu He1, Jiayue Han1,2, Xingwei Han1, Chao Han1, Lixin Liu1, Xiutao Yang1, He Yu1,2, Jun Gou1,2, and Jun Wang1,2*

1School of Optoelectronic Science and Engineering, University of Electronic Science and Technology of China, Chengdu 610054, China

2State Key Laboratory of Electronic Thin Films and Integrated Devices, University of Electronic Science and Technology of China, Chengdu 610054, China

*Corresponding author. E-mail: wjun@uestc.edu.cn

**This file includes:**

Texts 1-11

Tables S1-S5

Figures S1-S41

# Supplementary Text

# Text 1. Principle and actual processing of the water transfer printing method.

Water transfer printing (WTP) utilizes Marangoni flow, which arises from surface-tension gradients between solvent and water surfaces. When a polymer solution is deposited onto a water surface, differences in surface tension induce spontaneous spreading of the polymer solution towards regions of higher surface tension, a phenomenon known as the Marangoni effect. The spreading rate primarily depends on the diffusion coefficients of the polymer solution and the water substrate, as well as the volatility of the solvent. By adjusting the solution concentration and volume, films of controllable thickness can be obtained.

**Figure 1C** illustrates the procedure of transferring BHJ or PM6 layers using the WTP method. Initially, a solution is quantitatively dispensed onto the water surface and allowed to spread uniformly. After drying completely, the substrate is carefully placed on the polymer film floating on water. Excess film is trimmed away, and subsequently, the substrate with the transferred BHJ or PM6 layer is gently lifted. Additionally, this approach utilizes water as temporary support, facilitating the fabrication of large-area films. It is also compatible with flexible substrates, demonstrated in this work by successfully fabricating LTHJ OPDs on a 0.5 mm-thick polyethylene terephthalate (PET) substrate.

# Text 2. Calculation principle and process for surface energy measurement.

Initially, the polar liquid (H2O) and non-polar liquid diiodomethane (CH2I2) were selected to measure contact angles separately on the film surfaces.

The Owens-Wendt equation is known to be:

Where is the contact angle of the liquid on the solid surface, is the total surface energy of the liquid,  and are the dispersion and polarity components of the liquid, and and  are the dispersion and polarity components of the surface of the film to be measured.

Equations for water (H2O) and diiodomethane (CH2I2), respectively:

Solve for nd  by the above two equations.

The surface energy of the film surface is equal to the sum of the and the .

# Text 3. Mott-Schottky Analysis.

Electrochemical impedance spectroscopy (EIS) measurements were conducted using an electrochemical workstation to acquire impedance data. The imaginary part impedance obtained from the EIS is used to calculate the capacitance with the following equation

where frepresents the test frequency.

The trap density (*N*A) is defined as:

where *ε*r is the relative dielectric constant of the film (assumed to be 3), ε0 is the vacuum permittivity, *q* is the electronic charge, and *A* represents the active device area.

By performing linear fitting in the linear region of the Mott-Schottky plot, the device characteristics, including trap density and built-in potential, were extracted.

In addition, the depletion width (*W*depletion), also termed the space charge width, is given by:

where *V* is the applied bias voltage.

The wider *W*depletion indicates that the LTHJ OPD exhibits a strong blocking ability in the reverse injected charge.

# Text 4. Optimization analysis of energy band structure

In BHJ OPDs (**Figs. S10a and S11a**), direct contact between the donor/acceptor phases and electrodes enables electron and hole injection into the acceptor's LUMO and donor's HOMO, respectively, resulting in high dark current. In PHJ OPDs (**Figs. S10b and S11b**), the donor on the anode and acceptor on the cathode suppress charge injection, reducing dark current. Introducing a PM6 donor layer on the anode side (**Figs. S10c and S11c**) blocks electron injection, while adding a PY-IT acceptor layer on the cathode side (**Figs. S10d and S11d**) similarly suppresses hole injection. In the LTHJ structure (**Figs. 2G, S10e, and S11e**), additional donor and acceptor layers are incorporated at the anode and cathode interfaces, respectively, forming dual charge-blocking barriers that effectively prevent unwanted carrier injection. This design significantly reduces dark current and enhances sensitivity.

# Text 5. Diode equivalent circuit model.

According to the diode equivalent circuit model, the *I*-*V* characteristics of the device can be described by the following equation: .Where *k*B is the Boltzmann constant, *I*0 is the saturation current under reverse bias, *e* is the elementary charge, *T* is the temperature, *R*s and *R*sh represent the series and shunt resistances, respectively, and *n* is the ideality factor. Based on this model, **Fig. S14b** can be divided into three regions (I, II, and III), corresponding to different dominant components in the equivalent circuit governing the device’s *I*-*V* characteristics. The low-voltage region (Region I) is primarily influenced by trap-assisted recombination and *R*sh, where the suppressed current in LTHJ suggests a higher shunt resistance, effectively reducing leakage current. The intermediate voltage region (Region II) is governed by the saturation current and ideality factor, with LTHJ exhibiting the highest slope, indicating the lowest ideality factor. A lower ideality factor implies reduced recombination energy loss or decreased electronic disorder within the device. The high-voltage region (Region III) is mainly dominated by *R*s, where LTHJ demonstrates the smallest series resistance, contributing to an improved rectification ratio.

# Text 6. Corrected current-voltage analysis (*I*corr-*V*rev).

The *I*corr-*V*rev analysis is a crucial approach for investigating the dark current suppression mechanisms and influencing factors in OPDs. By analyzing the *I*corr curve, we can more accurately understand key physical processes such as charge transport, carrier injection, and trap state distribution in the device.

Specifically, the *I*corr-*V*rev analysis serves the following purposes:

(1) Eliminating the influence of series and shunt resistances, ensuring a more intrinsic evaluation of dark current behavior.

(2) Differentiating various dark current components, thereby clarifying their respective contributions to the total dark current.

The *I*corr is calculated using the following equation:

where *I* represents the apparent dark current, and *V*rev denotes the absolute value of the applied reverse bias voltage. *R*s and *R*sh correspond to the area-normalized series and shunt resistances, respectively. These resistance values are extracted from the differential resistance obtained from dark current-voltage (*I*-*V*) curves (**Figs. S12** and **S13**).

**Text 7. Comparison of LTHJ OPD with Commercial Si and InGaAs PDs.**

Silicon (Si) and InGaAs detectors are among the most commonly used commercial near-infrared photodetectors. The operating wavelength range of Si detectors is approximately 400 nm to 1100 nm, while InGaAs detectors operate in the range of 800 nm to 1700 nm. The LTHJ OPD proposed in this work has an operating wavelength range of approximately 400 nm to 1000 nm, but it offers advantages in terms of dark current and sensitivity. At a bias of -0.1 V, the dark current of the Si detector is approximately 3.02×10-9 A/cm2, and the dark current of the InGaAs detector is approximately 6.00×10-8 A/cm2, both of which are higher than the 1.74×10-11 A/cm2 dark current of the LTHJ OPD. The Si detector, InGaAs detector, and LTHJ OPD all exhibit an EQE of around 60% in their respective operating wavelength ranges, demonstrating comparable light response. At a bias of -0.1 V, the maximum detectivity of the Si detector is 1.58×1013 Jones, while the InGaAs detector has a maximum detectivity of 5.50×1012 Jones, both of which are lower than the LTHJ OPD’s 1.64×1014 Jones. For more data, please refer to **Figure S25**.

# Text 8. Principle and actual processing of the PDMS transfer printing method.

PDMS (polydimethylsiloxane) transfer printing leverages the adhesion difference between polymer films and PDMS substrates. Initially, the polymer solution is spin-coated onto a glass substrate and allowed to dry. A PDMS film is then placed onto the polymer-coated glass substrate and immersed in water for 2 minutes. Upon removal, the polymer film remains adhered to the PDMS film. Subsequently, the target substrate is placed in contact with the PDMS film, heated at 120 °C for 3 minutes, and gently separated, leaving the polymer film on the target substrate.

Compared to the water transfer process, PDMS transfer printing typically results in greater surface roughness, negatively affecting device performance, as illustrated in **Figure S28**. Achieving comparable dark current characteristics to those obtained by WTP necessitates thicker polymer films (~120 nm BHJ and ~60 nm PM6) when using the PDMS method (**Fig. 5D**). However, as shown in **Fig. 5E**, these thicker films also lead to inferior rectification ratios.

# Text 9. Comparison between PDMS and WTP processes.

**(1) Surface morphology:** The surface morphology of films prepared using WTP and PDMS was analyzed by AFM. The root-mean-square roughness (*R*q) of PM6 and BHJ films fabricated via WTP are 1.37 nm and 1.48 nm, respectively, while those prepared via PDMS exhibit higher *R*q values of 1.80 nm and 1.97 nm, indicating that WTP yields smoother films. Notably, PDMS-printed films frequently show defect sites with height variations of up to ~20 nm, which are absent in WTP films. This morphological difference may significantly affect the optoelectronic properties of the films, making it a critical factor in device fabrication.

**(2) Optoelectronic properties:** Under dark conditions, the device fabricated via WTP exhibits a current of 3.33×10-5 A at 1 V, while the device prepared using PDMS shows a lower current of 1.26×10-5 A. At -1 V, the WTP device has a current of 1.09×10-12 A, compared to 1.99×10-12 A for the PDMS device. Consequently, the rectification ratio of the WTP device reaches 3.05×107, significantly higher than that of the PDMS device (6.35×106). In terms of photocurrent response, no significant difference is observed between the devices prepared by the two transfer methods; both exhibit a responsivity of approximately 0.4 A/W around 800 nm.

**(3) Production time:** Production time is a critical parameter reflecting both fabrication efficiency and scalability. In this regard, the WTP process offers significant advantages over the PDMS-based method. Unlike PDMS transfer printing, which typically involves multiple steps such as mold fabrication, curing, and demolding, the WTP technique enables direct film transfer using water as the medium, thereby simplifying the overall process. This not only reduces the total processing time but also eliminates the cost and time associated with mold preparation. In our experiments, a complete WTP cycle can be completed within 100 seconds, making it a more efficient and scalable approach.

# Text 10. Implementation of O-ISAC using LTHJ OPDs.

Integrated Sensing and Communication (ISAC) technology integrates sensing and communication capabilities into a single platform, effectively utilizing shared spectrum and hardware resources to mitigate spectrum scarcity caused by the rapid growth of wireless devices [56]. ISAC is recognized as a key technology for the next generation of wireless communications [57, 58]. However, traditional radio frequency (RF)-based ISAC faces several inherent limitations:

(1) Scarce spectrum resources, with RF frequencies mainly limited in the 450-6000 MHz (low frequency) and 24250-52600 MHz (high frequency) bands.

(2) Limited data rates, typically limited to about 1 Gbps.

(3) High power consumption, especially at higher operating frequencies.

(4) Limited detection accuracy and environmental awareness.

Optical ISAC, which uses visible and near-infrared (NIR) light, addresses these limitations by providing [59-62].

(1) Significantly greater bandwidth due to the high-frequency nature of optical signals, supporting data rates in excess of 100 Gbps.

(2) Lower power consumption.

(3) Enabling precise physiological monitoring and biomedical diagnosis.

In this work, the proposed LTHJ OPD demonstrates superior sensitivity compared to conventional Si PD, enabling PPG detection at long-distance misalignment and reliable optical wireless communication through obstacles. The O-ISAC technology is expected to have a significant impact on various fields, including healthcare, industry, intelligent transportation, and defense.

# Text 11. Extended experimental section

**(1) Preparation of different active layer structures:**

PM6:PY-IT (BHJ1): A 100 nm thick PM6:PY-IT (1:1) film was prepared by spin-coating from a 14 mg mL-1 chloroform solution onto the PEIE layer for 45 s and annealed at 100 °C for 5 min.

PM6:PY-IT (BHJ2): Similar procedure as BHJ1, but prepared with a higher thickness.

PY-IT/PM6 (PHJ): PY-IT solution (10 mg mL-1 in chloroform) was spin-coated onto the PEIE layer for 45 s and annealed at 100 °C for 5 min. A PM6 layer was subsequently transferred onto the PY-IT layer via water transfer printing. After transfer, the device was evacuated for 2 min and annealed at 100 °C for 5 min.

PM6:PY-IT/PM6 (BHJ/PM6): PM6:PY-IT (1:1) was spin-coated from a chloroform solution (14 mg mL-1) onto PEIE for 45 s. Then, a PM6 layer was transferred onto the BHJ via water transfer printing, followed by vacuum treatment for 2 min and thermal annealing at 100 °C for 5 min.

PY-IT/PM6:PY-IT (PY-IT/BHJ): PY-IT was spin-coated from chloroform (10 mg mL-1) onto PEIE for 45 s and annealed at 100 °C for 5 min. Subsequently, PM6: PY-IT was transferred onto this layer using water transfer printing, then vacuum-treated for 2 min and annealed at 100 ℃ for 5 min.

PM6:Y6: A solution of PM6:Y6 (1:1.2, 16 mg mL-1) in chloroform was spin-coated onto PEIE at 2000 rpm for 45 s, achieving a film thickness of approximately 100 nm.

**(2) PPG Measurement:**

PPG signals were acquired by directly connecting photodetectors to a PDA analyzer without amplification or filtering, in a dark environment. An 850 nm LED powered by a DC source served as the illumination. For comparison, a commercial silicon detector (HAMAMATSU S1337) was employed, and its active detection region was reduced from 1 cm2 to 0.02 cm2 using a customized circular mask to match the active area of the organic device.

**(3) Optical wireless communication:**

A grayscale "smile" image was converted into a 40×40 matrix, then flattened into a one-dimensional sequence and normalized within the 0 to 5 range. This sequence was applied as voltage pulses to the laser. The resulting optical signal was detected by a photodetector, generating a current-time sequence. Finally, the measured currents were reshaped into a two-dimensional matrix and reconstructed into an image using the "imshow" function.

# Table S1. Introduction to photodetector-related terminology.

| Metric | Unit | Definition/Principle |
| --- | --- | --- |
| Photoinduced Current (*I*light) | A | Current flowing through the device under illumination. |
| Photocurrent (*I*ph) | A | Defined as *I*ph= *I*light-*I*dark |
| Current density (*J*) | A cm-2 | Current per unit area flowing through the device. |
| Short-circuit current density (*J*sc) | A cm-2 | Current density generated by the device at zero external bias voltage. |
| Photo-switching ratio (*Ilight*/*I*dark) | / | Ratio of the photocurrent under illumination to the dark current. |
| Responsivity (*R*) | A W-1 | Photocurrent generated per unit incident optical power. |
| Device area (*A*) | cm2 | Effective active area of the photodetector. |
| Noise spectrum (*i*n) | A2 Hz-1 | Spectral density of current fluctuations (noise). |
| Specific detectivity(*D**) | cm Hz1/2 W-1 | NEP normalized by device area and electrical bandwidth of the noise measurement. |
| Dark-current-limited specific detectivity (*Dsh**) | cm Hz1/2 W-1 | Specific detectivity calculated based on dark current. |
| 3dB bandwidth (BW) | Hz | Frequency at which the responsivity drops to of the steady-state value. |
| Surface energy (𝛾s) | mN·m-1 | Total surface energy of the material. |
| Dispersion component (𝛾sd) | mN·m-1 | Dispersive component of surface energy. |
| Polar component (𝛾sp) | mN·m-1 | Polar component of surface energy. |
| Shunt resistance (*Rsh*) | Ω | Shunt resistance of the device. |
| Series resistance (*Rs*) | Ω | Series resistance of the device. |
| Depletion width (*W*depletion) | nm | Charge carrier depletion region |
| Reverse voltage (*V*rev) | V | Magnitude of the externally applied reverse bias voltage. |
| Corrected current (*I*corr) | A | Current corrected for parasitic resistances. |
| Effective voltage (*V*eff) | V | Difference between built-in potential *V*0 (the voltage at which the photocurrent is zero, *I*ph=0) and applied voltage (*V*app). |
| External quantum efficiency (EQE) | / | Ratio of photogenerated electrons to incident photons. |
| Linear dynamic range (LDR) | dB | Range of incident optical power for linear photodetector response. |
| Response times (τr, τf) | μs | Rise time (τr): Time for photocurrent to rise from 10% to 90%. Fall time (τf): Time to fall from 90% to 10%. |
| Rectification ratios (η) | / | The ratio of forward to reverse current of a device under a specified voltage. |

# Table S2. High-Performance Organic Photodetectors Based on Vertical Phase Separation Strategy

| **Process Method** | **Photosensitive material** | **Detection wavelength**  **(nm)** | ***J*dark**  **(nA cm-2)** | ***R* (A W-1)** | ***D** (Jones)** | **LDR (dB)** | **Year** | **Ref.** |
| --- | --- | --- | --- | --- | --- | --- | --- | --- |
| **Annealing Process Optimization** | PBDB-T/ITIC | 300-800 | [1.33@0V](mailto:1.33@0V);  117@-0.5V | [0.387@688](mailto:0.387@688)nm | 2.17×[10](mailto:1012@-0.5V,)[12](mailto:1012@-0.5V,)[@-0.5V,](mailto:1012@-0.5V,) 688nm | / | 2020 | 34 |
| **Sequentially Solution-Processed** | P3HT/PCBM | 300-700 | 126@-1V | 0.256@620nm | ~8×[10](mailto:1012@-0.5V,)[11](mailto:1012@-0.5V,)[@-1V,](mailto:1012@-0.5V,) 620nm | / | 2015 | 63 |
|  | P3HT/PC71BM | 300-700 | 248@-0.5V | 0.302@550nm, -0.5V | 1.23×1012@550nm, -0.5V | / | 2018 | 64 |
|  | PTzBI-Ph/N2200 | 300-900 | 3.77@-0.1V | 0.252@-0.1V | 9.07×1012@-0.1V | / | 2019 | 65 |
|  | BTP-4F/D18-Cl | 300-1000 | 3.94@-0.2V | 0.47@850nm, -0.2V | 1.11×1013@850nm, -0.2V | 159.4@0 V | 2023 | 66 |
|  | P3HT/PC71BM:Y6 | 300-1000 | 15.1@-0.5V | [0.44@-0.5V,](mailto:0.44@-0.5V,) 790nm | 6.31×1012@790nm, -0.5V | / | 2024 | 67 |
|  | D18-Cl:PY-IT/L8-BO | 300-1000 | 0.138@-0.2V | [0.489@-0.2V,](mailto:0.44@-0.5V,) 805nm | [7.35×10](mailto:0.44@-0.5V,)[13](mailto:0.44@-0.5V,)[@-0.2V,](mailto:0.44@-0.5V,) 805nm | / | 2024 | 68 |
| **Protective Layer Strategy** | D18/Y6 | 350-1000 | 0.021@0V | [0.499@0V,](mailto:0.44@-0.5V,) 805nm | [1.97×10](mailto:0.44@-0.5V,)[14](mailto:0.44@-0.5V,)[@0V,](mailto:0.44@-0.5V,) 805nm | 83@0V | 2021 | 33 |
| **Material Improvement** | PBT(EDOT)/PC71BM | 300-750 | 0.16@-0.2V | ~0.27@-0.2V, 610nm | 3.5×[10](mailto:1013@-0.2V,)[13](mailto:1013@-0.2V,)[@-0.2V,](mailto:1013@-0.2V,) 610nm | / | 2015 | 69 |
|  | PM6-Br50/Y6 | 400-1000 | 0.403@-0.5V | 0.486@-0.5V | 4.27×[10](mailto:1013@-0.2V,)[13](mailto:1013@-0.2V,)[@-0.5V](mailto:1013@-0.2V,) | 73.7 | 2024 | 35 |
|  | P3HT/IDIC-DEG | 300-800 | 88.8@-10V | 4.81@-10V | 2.53×1013@-10V | / | 2024 | 36 |
| **Water Transfer Printing - Double Heterojunction** | PM6/Y6 | 400-1000 | 3.22@-0.5V | 0.46@780nm | 1.07×1013@780nm, | / | 2022 | 70 |
| **This work** | PM6/PY-IT | 300-1000 | 0.01068@-0V  0.01899@-0.1V  0.06213@-1V  0.09089@-2V | 0.405@810nm, -0.1V | 2.00×1014@810nm, -0V  1.64×1014@810 nm, -0.1V  1.04×1014@810 nm, -1V  0.89×1014@810 nm, -2V | 192.87@850nm, -0.1V | This work | |

# Table S3. Contact angles with water and CH2I2, total surface energy (γs), dispersion component (γsd), and polar component (γsp) of different layers used in LTHJ devices.

| **Layer** | **Contact angle (°)** | | **Surface energy (mN m-1)** | | |
| --- | --- | --- | --- | --- | --- |
|  | H2O | CH2I2 | *γsd* | *γsp* | *γs* |
| **PEIE** | 44.092 | 36.87 | 29.497 | 27.113 | 56.609 |
| **PY-IT** | 80.259 | 38.362 | 36.894 | 3.953 | 40.847 |
| **PM6:PY-IT** | 77.349 | 41.449 | 34.37 | 5.666 | 40.036 |
| **PM6** | 77.908 | 45.235 | 32.286 | 6.002 | 38.288 |
| **MoO3** | 63.431 | 49.978 | 26.157 | 16.265 | 42.422 |

# Table S4. Comparison of shunt resistance and series resistance of different devices

| **Active layer** | **Shunt Resistance (GΩ)** | **Series Resistance (Ω)** |
| --- | --- | --- |
| **PY-IT/PM6:PY-IT/PM6** | 1316 | 1202.29 |
| **PY-IT/PM6** | 187.021 | 2459.66 |
| **PM6:PY-IT/PM6** | 167.112 | 2047.92 |
| **PY-IT/PM6:PY-IT** | 44.683 | 1904.22 |
| **PM6:PY-IT** | 2.937 | 2166.85 |

# Table S5 High-Performance All-Polymer Organic Photodetectors

| **Photosensitive material** | **Type** | **Detection wavelength**  **(nm)** | ***J*dark**  **(nA/cm2)** | ***R* (A/W)** | ***D*sh* (Jones)** | **LDR (dB)** | **Year** | **Ref.** |
| --- | --- | --- | --- | --- | --- | --- | --- | --- |
| P3HT:P1 | PN | 300-900 | / | 0.097@570nm, -5V | 1.3×1011@610nm, -0.1V | / | 2015 | 71 |
| P1:PNDI  P2:PNDI  P3:PNDI | PN | 300-1100 | 9.2@-0.1V  24@-0.1V  1.5@-0.1V | 0.034@850nm, -2V  0.0468@850nm, -2V  0.1017@850nm, -2V | 4.4×1011@850nm, -0.1V  4.2×1011@850nm, -0.1V  3.4×1012@850nm, -0.1V | / | 2016 | 72 |
| PTB7-Th:PIIG-NDI (OD)  PTB7-Th:PIIG-PDI (EH)  PTB7-Th:PIIG-PDI (OD) | PN | 350-900 | [280@-0.2V,](mailto:75@-0.2V;)  1.55×103@-2V  [75@-0.2V,](mailto:75@-0.2V;)  677@-2V  [120@-0.2V,](mailto:75@-0.2V;)  545@-2V | 0.12@710nm, -1V  0.12@710nm, -1V  0.085@710nm, -1V | ~1.5×1012@710nm, -0.2V  6×1011@710nm, -0.2V  ~2.5×1011@710nm, -0.2V | / | 2016 | 73 |
| PolyD:PolyA  PolyD:PolyAA′25  PolyD:PolyAA′50  PolyD:PolyAA′75  PolyD:PolyA′  PolyD:PolyA/A′25  PolyD:PolyA/A′50  PolyD:PolyA/A′75 | PN | 300-1000 | [8.5@-0.1V](mailto:1.2@-0.1V)  [1.7@-0.1V](mailto:1.2@-0.1V)  [1.2@-0.1V](mailto:1.2@-0.1V)  [7.3@-0.1V](mailto:1.2@-0.1V)  [14@-0.1V](mailto:1.2@-0.1V)  [11@-0.1V](mailto:1.2@-0.1V)  14@-0.1V  44@-0.1V | 0.0533@900nm, -0.1V  0.0935@900nm, -0.1V  0.0895@900nm, -0.1V  0.0781@900nm, -0.1V  0.0554@900nm, -0.1V  0.0460@900nm, -0.1V  0.0529@900nm, -0.1V  0.0516@900nm, -0.1V | 1×1012@900nm, -0.1V  4.7×1012@900nm, -0.1V  4.7×1012@900nm, -0.1V  1.6×1012@900nm, -0.1V  8.2×1011@900nm, -0.1V  7.7×1011@900nm, -0.1V  8.0×1011@900nm, -0.1V  4.4×1012@900nm, -0.1V | / | 2017 | 74 |
| PDTP-DPP:PNDI  PDTP-DPP:PNDI-DPP10  PDTP-DPP:PNDI-DPP30  PDTP-DPP:PNDI-DPP50 | PN | 300-1000 | 7.6@-0.1 V  1.3@-0.1 V  5.4@-0.1 V  5.5@-0.1 V | 0.0360@900nm, -0.1V  0.0491@900nm, -0.1V  0.299@900nm, -0.1V  0.149@900nm, -0.1V | 7.3×1011@900nm, -0.1V  2.4×1012@900nm, -0.1V  7.2×1011@900nm, -0.1V  3.5×1010@900nm, -0.1V | / | 2017 | 75 |
| PBDB-T:PNDI-FT10 | PN | 350-850 | 11@-3V | 0.34@-3V, 630nm | 5.8×1012@-3 V, 650 nm | 105@-3V | 2018 | 76 |
| PTB7-TH:PNDI-5DD  PTB7-TH:PNDI-2OD  PTB7-TH:PNDI-POD | PN | 300-900 | 0.12@-0.1  8.6@-0.1  7.1@-0.1 | 0.173@700nm, -0.1 V  0.213@700nm, -0.1 V  0.127@700nm, -0.1 V | [3.0×10](mailto:1.96×1013@960nm,-0.1V)[13](mailto:1.96×1013@960nm,-0.1V)[@700nm](mailto:1.96×1013@960nm,-0.1V), -0.1V  [4.0×10](mailto:1.96×1013@960nm,-0.1V)[12](mailto:1.96×1013@960nm,-0.1V)[@700nm](mailto:1.96×1013@960nm,-0.1V), -0.1V  [2.7×10](mailto:2.7×1012@700nm,-0.1)[12](mailto:2.7×1012@700nm,-0.1)[@700nm](mailto:2.7×1012@700nm,-0.1), -0.1V | / | 2018 | 77 |
| P3HT:PIDT-2TPD | PN | 300-700 | 2.85@-5V | 0.16@610nm, -5V | 1.1×1012@-5V, 610nm | / | 2018 | 78 |
| P3HT:PZ1 | PM | 300-800 | 1×103@-5V | / | 6.1×1012@-20V, 615nm | / | 2019 | 79 |
| NT40:N2200 | PN | 300-900 | 4.85@0V | [0.33@](mailto:0.33@-0.1V,)720nm, -0.1V | 2.61×1013@720nm, -0.1V | 96.6@720nm, -0.1V | 2019 | 80 |
| PBDB-T:PZ1 | PM | 300-850 | 5.5×103@-10V | ~8@670nm,-20V | 1×1012@675nm, -10V | / | 2019 | 81 |
| PTzBl-Ph/N2200 | LBL | 300-850 | ~200@-2V | 0.25@600nm, -0.1V | 5.68×1012@-0.1V, 600nm | / | 2019 | 58 |
| TQ1:PNDI-T10  TQ1/PNDI-T10  PTB7-Th:PNDI-T10  PTB7-Th/PNDI-T10 | PN/LBL | 300-900 | 0.582@0V  0.123@0V  1.48@0V  1.03@0V | ~0.065@630nm  ~0.021@630nm  ~0.175@700nm  ~0.02@700nm | ~2×1011@630 nm  ~6.5× 1010@630 nm  ~3.5×1011@700 nm  ~3.5×1010@700 nm | 75@635nm  65@635nm  75@635nm  65@635nm | 2020 | 82 |
| PSBOTZ:PNDBO  PSBOTz:NDI | PN | 400-600 | 0.21@-2V  53@-2V | 0.086@530nm, -2V  0.059@530nm, -2V | 1.1×1013@530nm, -2V  4.6×1011@530nm, -2V | / | 2020 | 83 |
| PMBBDT:N2200 | PM | 300-1000 | ~8×103@-2V | ~10@810nm,-2V | ~8× 1011@810nm, -2V | 140@650nm, -2V | 2021 | 84 |
| PBDB-T:PNDI-DTBT  PBDB-T:PNDI-DTBT  PBDB-T:PNDI-DTBT | PN | 300-900 | 13.2@ -0.1V  17.2@ -0.1V  116@-0.1V | 0.31@660nm, -0.1V  0.20@660nm, -0.1V  0.20@660nm, -0.1V | 4.77×1012@660nm, -0.1V  2.70×1012@660nm, -0.1V  1.04×1012@660nm, -0.1V | / | 2021 | 24 |
| PM6:PY-IT | PN | 400-1000 | 151@-1V | 0.47@-1V, 530nm | 2.15×1012@-1V, 530nm | 80.5@-1V, 530nm | 2022 | 85 |
| PBDB-T:PYF-T-o | PM | 310-900 | ~2.5×103@-4V | ~61.7@850nm, -4V | 4.2×1012@850nm, -4V | / | 2022 | 86 |
| PSBOTz:PNE1  PSBOTz:PNE2  PSBOTz:PNE3  PSBOTz:PPE1 | PN | 350-750 | 4.10@-2V  13.7@-2V  1.23×103@-2V  5.10@-2V | 0.065@532nm, -2V  0.0183@532nm, -2V  0.0372@532nm, -2V  0.0432@532nm, -2V | 1.81×1012@532nm, -2V  2.76× 1011@532nm, -2V  5.93×1010@532nm, -2V  1.07× 1012@532nm, -2V | 121@532nm, -2V  98@532nm, -2V  64@532nm, -2V  113@532nm, -2V | 2022 | 87 |
| J70:P1  J70:P2  J70:P3  J70:P4 | PN | 300-900 | [5.6@-0.1V](mailto:0.19@-0.1V)  [2.9@-0.1V](mailto:0.19@-0.1V)  0.85@-0.1V  0.19@-0.1V | 0.1551@580nm, -0.1V  0.1295@580nm, -0.1V  0.1039@580nm, -0.1V  0.1364@580nm, -0.1V | 3.6×1012@580nm, -0.1V  4.2×1012@580nm, -0.1V  6.3×1012@580nm, -0.1V  1.8×1013@580nm, -0.1V | [71.5@-0.1V](mailto:109.2@-0.1V)  [72.0@-0.1V](mailto:109.2@-0.1V)  [72.5@-0.1V](mailto:109.2@-0.1V)  109.2@-0.1V | 2022 | 88 |
| PBDB-T:PY3Se-1V | PM | 920-1000 | ~3×103@-20V | 28@960nm, -50V | / | 110@960nm, -20V | 2022 | 89 |
| TQ1:N2200 | PN | 400-800 | ~8×103@-2V | 0.034@610nm, 0V | 1.9×1011@600nm, 0V | 83.27@600nm | 2022 | 90 |
| PBDTT-FTTE:PNNTH | PN | 400-800 | / | ~0.18@710nm, -1V | ~3×1011@710nm, -1V | / | 2022 | 91 |
| PBDB-T:BTR:BTPV-4F | PM | 300-750 | 2×103@-10V | 102@660nm, -20V | 4.67×1010@660nm, -10V | / | 2022 | 92 |
| PBDB-T:N2200 | PN | 300-900 | 1.4@-2V | 0.42@620nm, -2V | 6.23×1012@620nm, -2V | / | 2022 | 50 |
| PPCPD:N2200 | PN | 300-1300 | 3.51@-0.1V | 0.077@1150nm, -0.1V | 3.08×1012@1150nm, -0.1V | / | 2023 | 93 |
| PM6:PY-IT | PN | 400-1000 | 0.592@-1V | 0.46@820nm, -3V | 2.98×1013@530 nm, -1V | 138@530 nm, -1V | 2023 | 94 |
| PBDB-T:N2200:PS | PM | 400-800 | 546@-12V | 10@400-700nm, -12V | 4.0×1013@-12 V, 450nm | 113.62@-12 V, 405nm | 2023 | 95 |
| P3HT/PY-IT | LBL/PM | 300-1000 | ~2×103@-10V | ~28.7@360nm, -12V | 4.4×1013@630nm, -12V | 103@-10V | 2023 | 96 |
| PTzBI-EHp-BTBHTx:N2200-BTBHTx | PN | 300-900 | / | / | 5.35×1013@600nm, -0.1V | / | 2023 | 97 |
| P3HT:PY-IT | PM | 300-1000 | 1.8@-10V | 8.4@630nm, -10V | 9×1013@630nm, -0.1V | 122@-10V | 2023 | 98 |
| J71:P（NDI-T-TPD）  PM6:P（NDI-T-TPD）  PTB7-TH:P（NDI-T-TPD）  PTB7-TH:P（NDI-T-DPP） | PN | 300-1000 | [2.2@-0.1V](mailto:2.2@-0.1V)  [0.61@-0.1V](mailto:0.61@-0.1V)  [0.23@-0.1V](mailto:0.61@-0.1V)  0.22@-0.1V | 0.2341@580nm, -0.1V  0.2629@620nm, -0.1V  0.2227@700nm, -0.1V  0.0347@720nm, -0.1V | 8.8×1012@580nm, -0.1V  1.9×1013@62 0nm, -0.1V  2.6×1013@720nm, -0.1V  4.2×1012@720nm, -0.1V | 74.4@-0.1V  [90.1@-0.1V](mailto:50@-0.1V)  [110.1@-0.1V](mailto:50@-0.1V)  [111.8@-0.1V](mailto:50@-0.1V) | 2023 | 99 |
| P3HT:PMBBDT:PTz-PT | PM | 300-1100 | ~2×103@-12V | ~10.89@900nm, -12V | 1.9×1013@850nm, -10V; | / | 2024 | 100 |
| PM6:PY-DTT  PM6:PY-DTP-C  PM6:PY-DTP-Ph | PN | 300-1000 | 0.91@-0.1V  0.069@-0.1V  0.092@-0.1V | 0.4073@800 nm, -0.1V  0.055@800 nm, -0.1V  0.2921@800 nm, -0.1V | 2.4×1013@800 nm, -0.1V  1.2× 1013@800 nm, -0.1V  5.4×1013@800 nm, -0.1V | 105@800 nm, -0.1V | 2024 | 101 |
| J71:P1  J71:P2  J71:P3  J71:P4  PM6:P1  PM6:P2  PM6:P3  PM6:P4  PTB7-TH:P1  PTB7-TH:P2  PTB7-TH:P3  PTB7-TH:P4 | PN | 300-900 | [0.036@-0.1V](mailto:0.033@-0.1V)  [0.23@-0.1V](mailto:0.033@-0.1V)  [0.11@-0.1V](mailto:0.033@-0.1V)  0.033@-0.1V  [0.049@-0.1V](mailto:0.033@-0.1V)  [0.046@-0.1V](mailto:0.033@-0.1V)  [0.049@-0.1V](mailto:0.033@-0.1V)  0.033@-0.1V  [210@-0.1V](mailto:0.033@-0.1V)  [0.32@-0.1V](mailto:0.033@-0.1V)  [17@-0.1V](mailto:0.033@-0.1V)  0.044@-0.1V | 0.212@800nm, -0.1V  0.364@800nm, -0.1V  0.322@800nm, -0.1V  0.438@800nm, -0.1V  0.379@800nm, -0.1V  0.344@800nm, -0.1V  0.339@800nm, -0.1V  0.432@800nm, -0.1V  0.154@800nm, -0.1V  0.195@800nm, -0.1V  0.212@800nm, -0.1V  0.300@800nm, -0.1V | 6.3×1013@800nm, -0.1V  4.3×1013@800nm, -0.1V  5.5×1013@800nm, -0.1V  1.4×1014@800nm, -0.1V  9.5×1013@800nm, -0.1V  8.9×1013@800nm, -0.1V  8.5×1013@800nm, -0.1V  1.3×1014@800nm, -0.1V  6.0×1011@800nm, -0.1V  1.9×1013@800nm, -0.1V  2.8× 1012@800nm, -0.1V  8.0×1013@800nm, -0.1V | 41@-0.1V  [50@-0.1V](mailto:50@-0.1V)  [70@-0.1V](mailto:50@-0.1V)  [89@-0.1V](mailto:50@-0.1V)  89@-0.1V  [87@-0.1V](mailto:50@-0.1V)  [88@-0.1V](mailto:50@-0.1V)  [88@-0.1V](mailto:50@-0.1V)  72@-0.1V  [70@-0.1V](mailto:50@-0.1V)  [49@-0.1V](mailto:50@-0.1V)  [83@-0.1V](mailto:50@-0.1V) | 2024 | 102 |
| PBDB-T:PY-IT:PZF-V | PN | 300-1000 | 0.5@0V  7@-0.1V  50@-1V | 0.44@860nm, -0.1V | 8×1012@860nm, -0.1V | 145@830 nm, -0.1V | 2024 | 103 |
| P4TOC-BT:P4TOC-DCBT  P4TOC-BT:P4TOC-DCBSe | PN | 400-1300 | 0.017@0V  0.025@0V | [0.11@960nm,0V](mailto:0.11@960nm,-0.1V)  0.06@880nm,0V | [4.73×10](mailto:4.73×1013@960nm,-0.1V)[13](mailto:4.73×1013@960nm,-0.1V)[@960nm,0V](mailto:4.73×1013@960nm,-0.1V)  1.96×1013@880nm,0V | 125@1050nm  132@1050nm | 2024 | 104 |
| J71:N2200  J71:N2200:ITIC  J71:NTI | PN | 300-850 | 0.081@-0.1 V  0.035@-0.1 V  0.016@-0.1 V | 0.146@575nm, -0.1V  0.166@575nm, -0.1V  0.266@575nm, -0.1V | 2.9×1013@575nm, -0.1V  4.9×1013@575nm, -0.1V  1.2×1014@575nm, -0.1V | 87@-0.1V  98@-0.1V  105@-0.1V | 2024 | 105 |
| PM6:PY-IT | PN | 300-1000 | [0.01068@-0V](mailto:0.01068@-0.1V)  [0.01899@-0.1V](mailto:0.01899@-0.1V)  0.06213@-1V  0.09089@-2V | 0.405@810nm, -0.1V | 2×1014@810nm, -0V  1.64×1014@810nm, -0.1V  1.04×1014@810nm, -1V  0.89×1014@810nm, -2V | 192.87@850nm, -0.1V | This work | |

Figure S1 Molecular chemical structures of PM6, PY-IT, PTB7-TH and PYF-T-o. (a) 3D structure of PM6. (b) 2D structure of PM6. (c) 3D structure of PY-IT. (d) 2D structure of PY-IT. (e) 3D structure of PTB7-TH. (f) 2D structure of PTB7-TH. (g) 3D structure of PYF-T-o. (h) 2D structure of PYF-T-o.

Figure S2 Optical absorption characteristics of materials. (a) Normalized absorption spectrum of PM6 and PY-IT. (b) Normalized absorption spectrum of the PM6:PY-IT BHJ.

Figure S3 X-ray photoelectron spectroscopy (XPS) of LTHJ film. (a) XPS spectra of N1s at different etching times (0 s, 450 s, and 900 s). (b) XPS survey spectrum after 450 sec of etching. (c, d) Normalized atomic concentration of N1s and F1s as a function of etching time

Figure S4 The actual processing of the water transfer printing method.

Figure S5 (a) PHJ film and (b) BHJ film.

Figure S6 Schematic illustration of different device structures. (a) PY-IT/PM6:PY-IT/PM6 (LTHJ) OPD. (b) PY-IT/PM6 (PHJ) OPD. (c) PM6:PY-IT/PM6 (BHJ/PM6) OPD. (d) PM6:PY-IT (BHJ) OPD. (e) PY-IT/PM6:PY-IT (PY-IT/BHJ) OPD.

Figure S7 Contact Angle Measurements of Various Materials. (a-e) Water contact angle measurements for PEIE, PY-IT, PM6:PY-IY BHJ, PM6 and MoO3. (f-j) CH2I2 contact angle measurements for PEIE, PY-IT, PM6:PY-IY BHJ, PM6 and MoO3.

Figure S8 Schematic illustration of the device structures for (a) hole-only and (b) electron-only devices used in this study.

Figure S9 Current-voltage characteristics of electron-only devices with BHJ and LTHJ active layers.

Figure S10 Operating mechanisms of different devices under reverse bias in the dark. (a) PY-IT:PM6 (BHJ). (b) PY-IT/PM6 (PHJ) OPD. (c) PM6:PY-IT/PM6 (BHJ/PM6) OPD. (d) PY-IT/PM6:PY-IT (PY-IT/BHJ) OPD. (e) PY-IT/PM6:PY-IT/ PM6 (LTHJ) OPD.

Figure S11 Operating mechanisms of different devices under forward bias in the dark. (a) PY-IT:PM6 (BHJ). (b) PY-IT/PM6 (PHJ) OPD. (c) PM6:PY-IT/PM6 (BHJ/PM6) OPD. (d) PY-IT/PM6:PY-IT (PY-IT/BHJ) OPD. (e) PY-IT/PM6:PY-IT/ PM6 (LTHJ) OPD.

Figure S12 Shunt resistances of different devices derived from the dark *I*-*V* curves. (a) LTHJ OPD. (b) PHJ OPD. (c) BHJ/PM6 OPD. (d) PY-IT/BHJ OPD. (e) BHJ OPD. *R*sh is determined as the inverse slope around *I* (0 mW, 0 V).

Figure S13 Series resistances of different devices derived from the dark *I*-*V* curves. (a) LTHJ OPD. (b) PHJ OPD. (c) BHJ/PM6 OPD. (d) PY-IT/BHJ OPD. (e) BHJ OPD. *R*s is determined as the inverse slope around *I* (0 mW, 1.5 V).

Figure S14 (a) Dark current characteristics of different devices under forward bias. (b) Dark current characteristics of different devices under reverse bias.

Figure S15 Operating mechanisms of different devices under reverse bias in the presence of light. (a) PY-IT:PM6 (BHJ). (b) PY-IT/PM6 (PHJ) OPD. (c) PM6:PY-IT/PM6 (BHJ/PM6) OPD. (d) PY-IT/PM6:PY-IT (PY-IT/BHJ) OPD. (e) PY-IT/PM6:PY-IT/ PM6 (LTHJ) OPD.

Figure S16 (a-e) Specific detectivity (*D**) as a function of frequency and wavelength for different devices at -0.1 V.

Figure S17 *I*-*T* curves of the LTHJ OPD measured under dark conditions at *V*ds = 0 V.

Figure S18 *D*sh* of the LTHJ OPD at various voltages.

Figure S19 Bias-dependent noise spectrum of the LTHJ OPD.

Figure S20 *D** as a function of frequency and wavelength of the LTHJ OPD at -1V.

Figure S21 Weak light detection capability of LTHJ OPDs.

Figure S22 Frequency response analysis of LTHJ OPD. (a) 3dB bandwidth (BW) of the LTHJ OPD. (b) Phototransient response of the device under modulated light signals at 640 Hz (top) and 160 kHz (bottom).

Figure S23 Performance comparison of LTHJ OPD with reported all-polymer OPDs. (a-c) Comparison of (a) dark current, (b) specific detectivity, and (c) responsivity as a function of wavelength between the LTHJ OPD and previously reported all-polymer OPDs (Table S5).

Figure S24 Performance comparison of the LTHJ OPD with reported all-polymer OPDs (**with reference labels**, **Table S5**).

Figure S25 Performance metrics of Si and InGaAs photodetectors. (a, c) Dark *I*-*V* characteristics of (a)Si and (c)InGaAs PDs. (b, d) EQE, responsivity, and *D*sh* spectra of (b)Si and (d)InGaAs PDs.

Figure S26 Stability analysis of the LTHJ OPD: Variance and maximum offset of *I*light and *I*dark in LTHJ OPD under continuous cyclic irradiation of 850 nm light over 1000 s at *V*ds = -10V. (a) Variance of *I*light. (b) Variance of *I*dark. (c) Maximum offset of *I*light. (d) Maximum offset of *I*dark.

Figure S27 Dark current characteristics of (a) LTHJ OPD and (b) BHJ OPD devices, comparing their initial performance (pristine) and after 20 days of storage under N2 ambient conditions.

Figure S28 AFM analysis of film morphology for PDMS and water transfer printing. (a, b) 3D and 2D AFM images of the PM6:PY-IT BHJ film prepared via water transfer printing. (c) Height profile along the white dashed line in (b). (d, e) 3D and 2D AFM images of the PM6 film prepared via water transfer printing. (f) Height profile along the white dashed line in (e). (g, h) 3D and 2D AFM images of the PM6:PY-IT BHJ film prepared via PDMS transfer printing. (i) Height profile along the white dashed line in (h). (j, k) 3D and 2D AFM images of the PM6 film prepared via PDMS transfer printing. (l) Height profile along the white dashed line in (k).


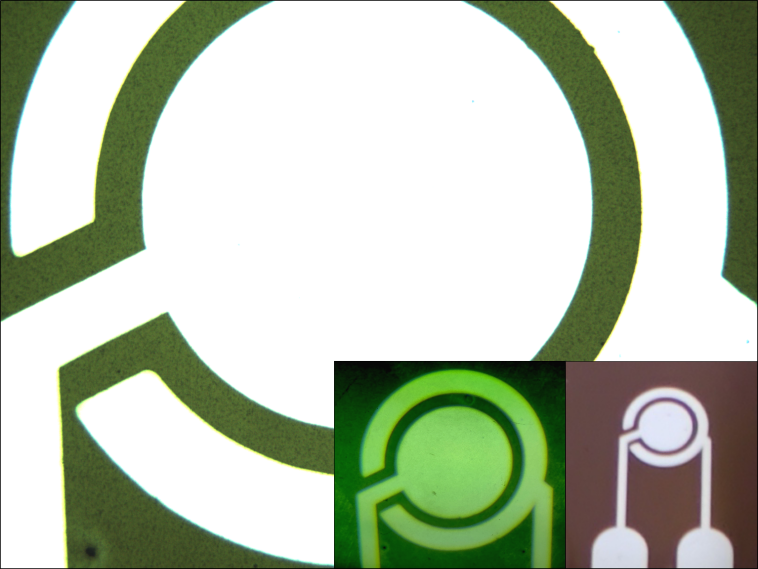


Figure S29 Optical image of the optical ISAC system based on LTHJ OPDs.

Figure S30 *I*-*V* characteristics of the optical ISAC system using LTHJ OPDs.

Figure S31 (a) Schematic of optical wireless communication without an obstacle. (b) Schematic of PPG signal acquisition under close-range alignment.

Figure S32: (a, b) PPG signals collected using (a) LTHJ OPD and (b) Si PD under close-range alignment and long-distance misalignment with an 850 nm NIR LED as the light source.

Figure S33 Heart rate calculation from FFT analysis of PPG signals using LTHJ OPD.

Figure S34 Imaging results of the OWC system with different obstacle movement scenarios. (a) The obstacle passes over the LTHJ OPD once. The left half shows the result without the obstacle (w/o obstacle), and the right half shows the result with the obstacle (w/ obstacle). (b) The obstacle passes over the LTHJ OPD twice, with the image alternating between the w/o and w/ obstacle conditions from left to right. (c) The obstacle passes four times, increasing the level of interference in the imaging. (d) The obstacle moves continuously and rapidly above the device in random directions (up/down/left/right), resulting in a high level of interference.

Figure S35 Heartbeat monitoring using the LTHJ OPD under interfering light conditions. (a) Schematic diagram of the measurement setup for heartbeat signal detection under interfering light. (b) Heartbeat signals recorded in darkness and under various interfering light sources.

Figure S36 Current–voltage characteristics of the flexible LTHJ OPD on a PET substrate measured under (a) the normal state and (b) the bent state (substrate area = 15 × 15 mm2; bending radius = 15 mm).

Figure S37 Flexible LTHJ OPD on PET substrate fabricated via water transfer printing. (a) Optical image of the flexible LTHJ OPD. (b) Current-voltage characteristics of the flexible LTHJ OPD under dark and 850nm illuminated conditions. (c) Schematic diagram of the image scanning system. (d) Current mapping of the scanned image displaying "123" under 850 nm illumination.

Figure S38 PPG signals collected using the flexible LTHJ OPD on a PET substrate under illumination at different wavelengths (450 nm, 520 nm, 650 nm, 780 nm, 808 nm, and 850 nm).

Figure S39 Optical wireless communication using the flexible LTHJ OPD abricated on a PET substrate. (a) Input signal encoding the message "UESTC" in a binary format for optical wireless communication. (b) Received optical signal detected by the flexible LTHJ OPD under 850 nm and 895 nm illumination.

Figure S40 Performance of the LTHJ OPD at 0 V, operating in self-powered mode, highlighting its potential for application in portable and wearable systems.

Figure S41 Performance characterization of LTHJ OPD based on PM6 and PYF-T-o. (a) Absorption spectrum of PM6 and PYF-T-o. (b) Absorption spectrum of PM6:PYF-T-o BHJ. (c) Dark current characteristics of the LTHJ OPD and BHJ OPD. (d) EQE and responsivity of the LTHJ device at *V*ds = -0.1 V. (e) Specific detectivity (*D*sh*) of the LTHJ OPD at different voltages.

Figure S42 Performance characterization of LTHJ OPD based on PTB7-TH and PY-IT. (a) Absorption spectrum of PTB7-TH and PY-IT. (b) Absorption spectrum of PTB7-TH:PY-IT BHJ. (c) Dark current characteristics of the LTHJ OPD and BHJ OPD. (d) EQE and responsivity of the LTHJ device at *V*ds = -0.1 V. (e) Specific detectivity (*D*sh*) of the LTHJ OPD at different voltages.
