# Supplementary figures and images for: Ultra-Sensitive All-Polymer Near-Infrared Photodetectors via Van der Waals Layered Triple Heterojunction
Source: Research (Wash D C). 2025 Oct 3;8:0939. doi: 10.34133/research.0939 (PMC12491782; doi:10.34133/research.0939)

**a**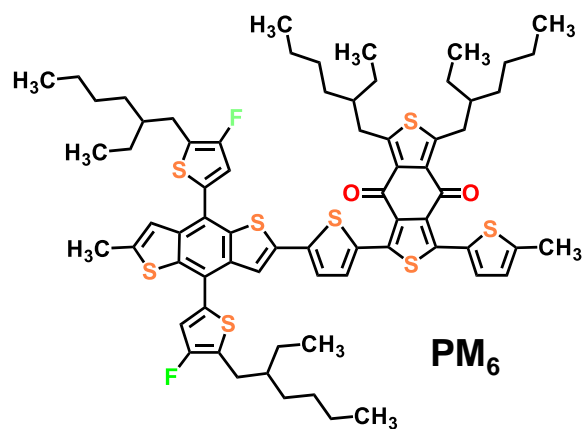**b**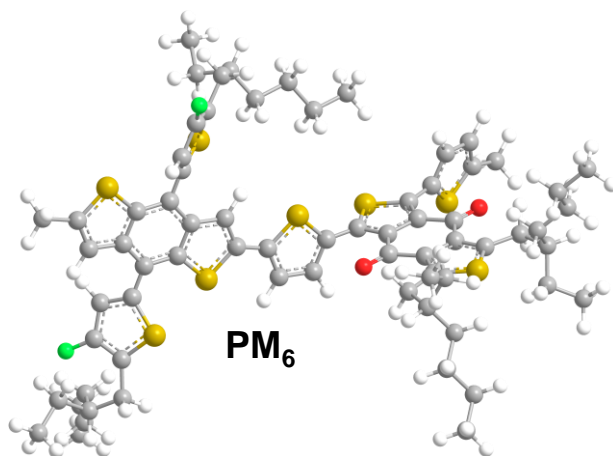**c**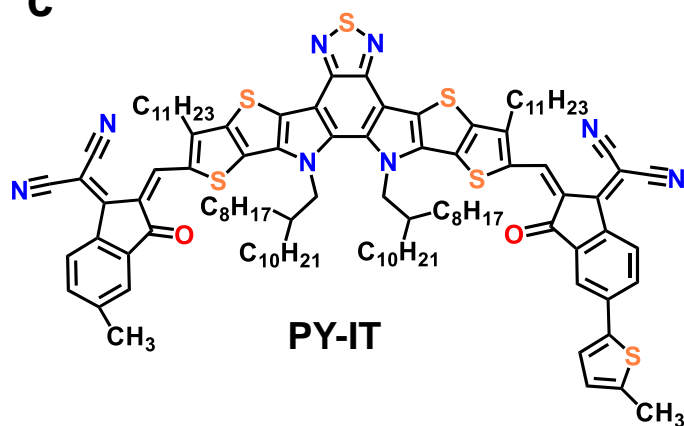**d**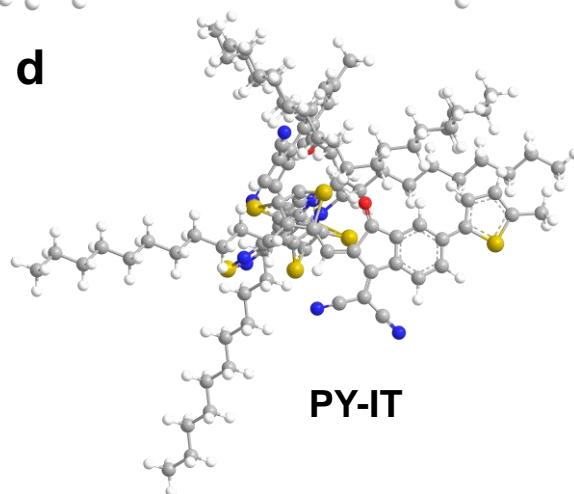**e**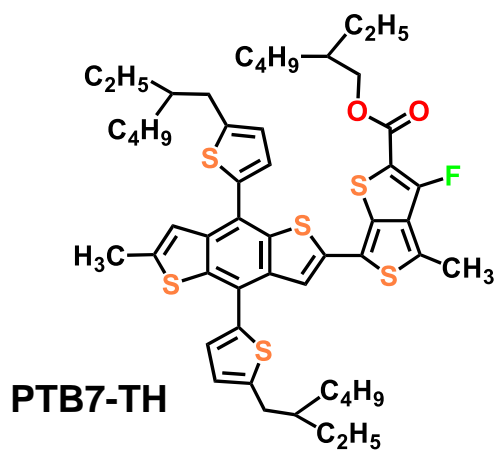**f**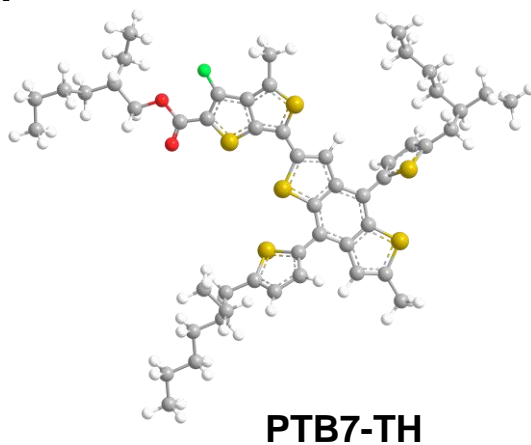**g**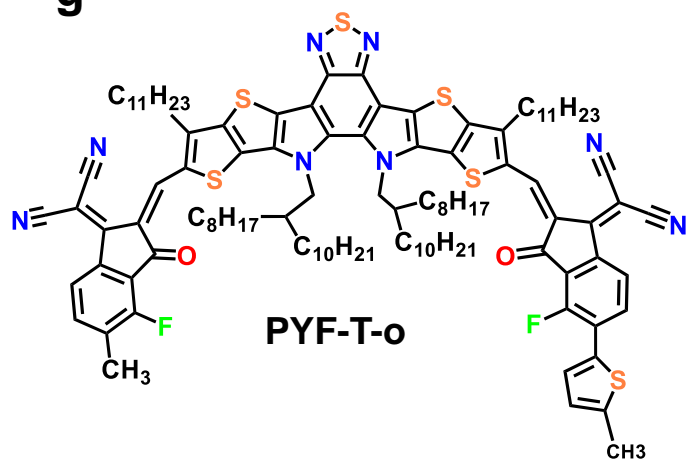**h**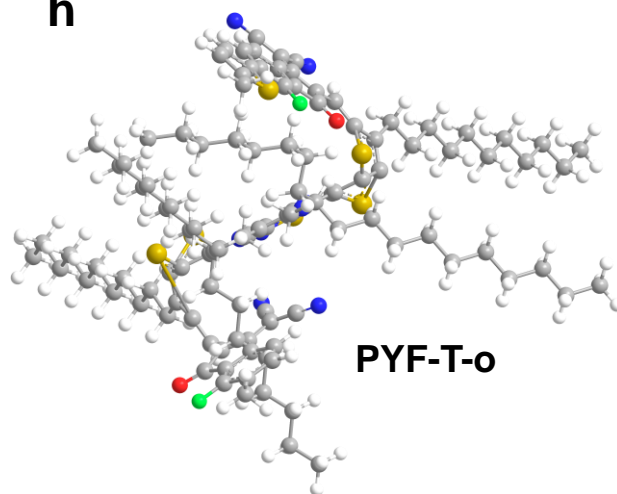

Supplement: Supplementary 1 — Texts S1 to S11 Tables S1 to S5 Figs. S1 to S42 References [56–105] [file research.0939.f1.zip › Figure S1.pdf]

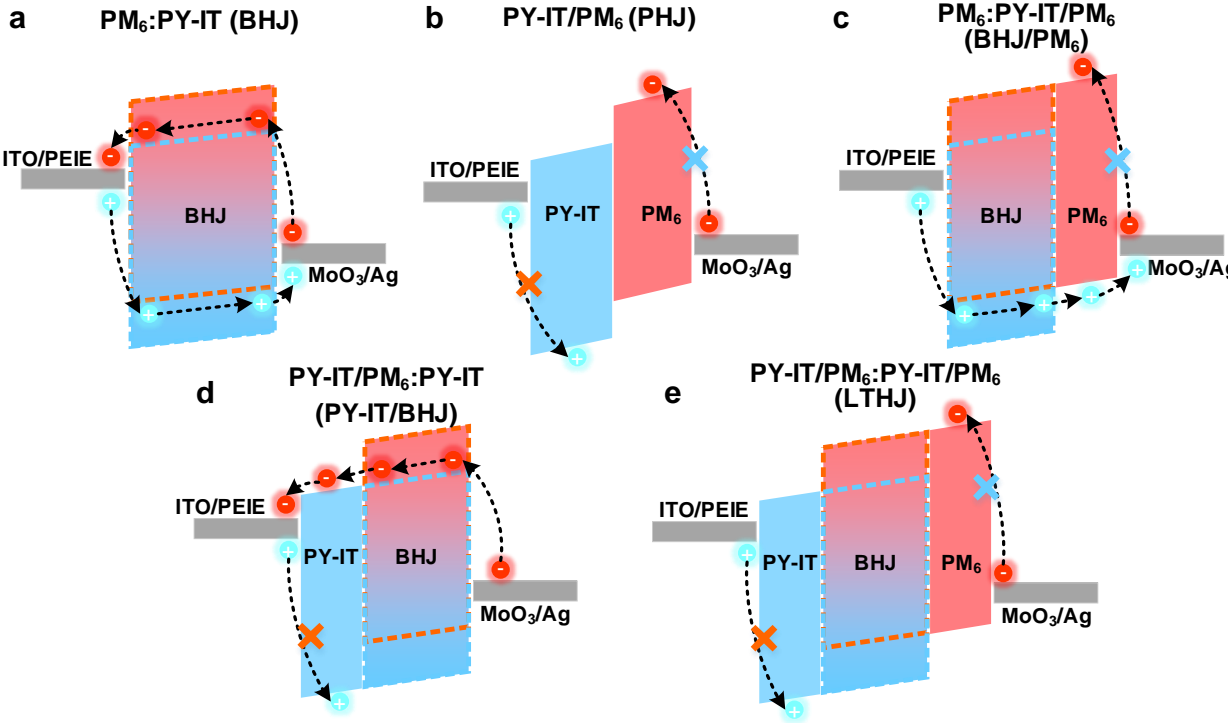

Supplement: Supplementary 1 — Texts S1 to S11 Tables S1 to S5 Figs. S1 to S42 References [56–105] [file research.0939.f1.zip › Figure S10.pdf]

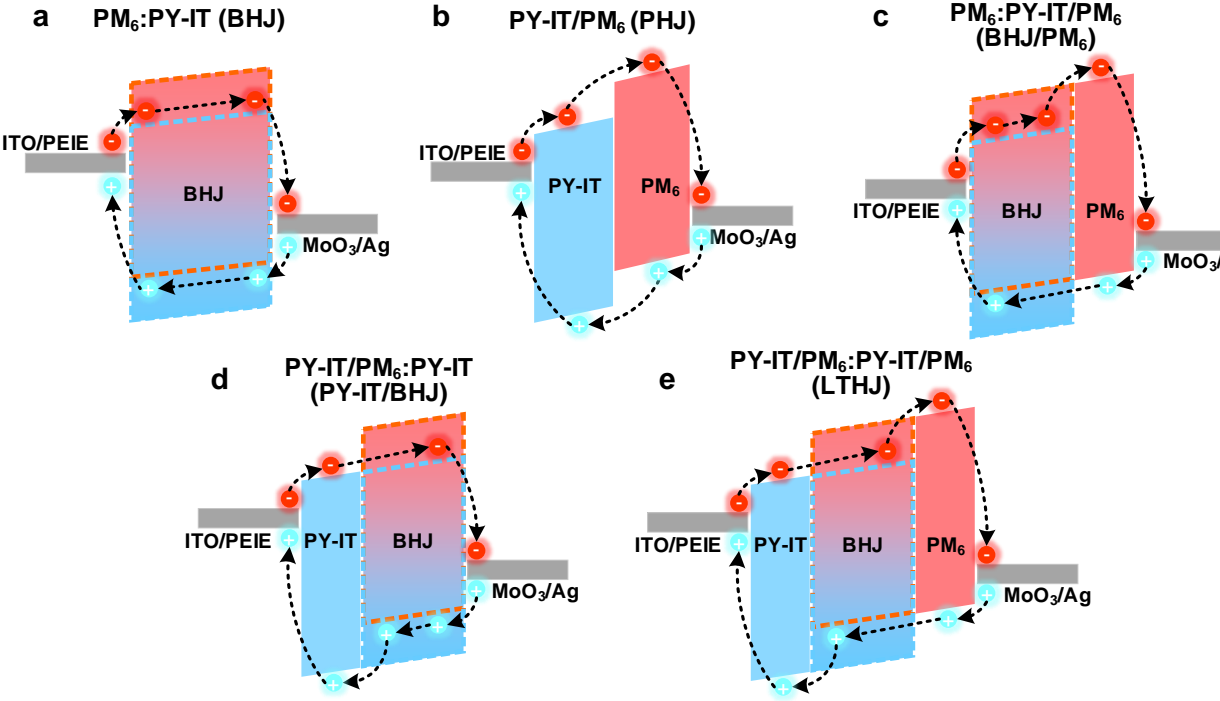

Supplement: Supplementary 1 — Texts S1 to S11 Tables S1 to S5 Figs. S1 to S42 References [56–105] [file research.0939.f1.zip › Figure S11.pdf]

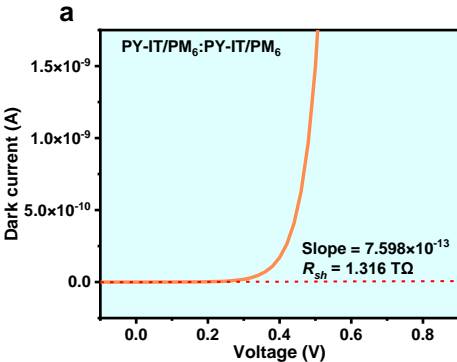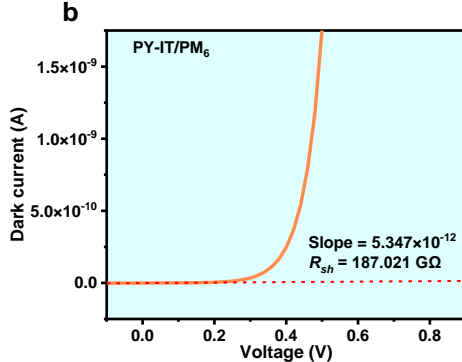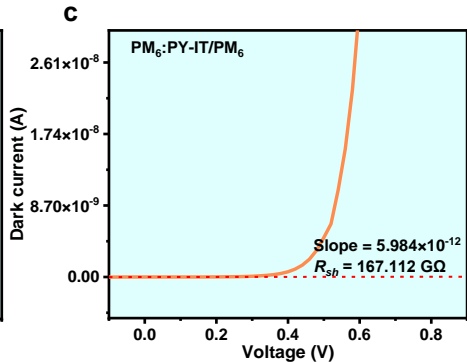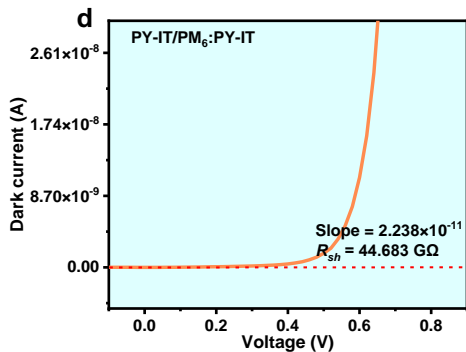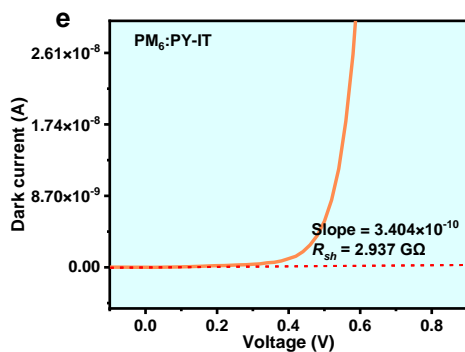

Supplement: Supplementary 1 — Texts S1 to S11 Tables S1 to S5 Figs. S1 to S42 References [56–105] [file research.0939.f1.zip › Figure S12.pdf]

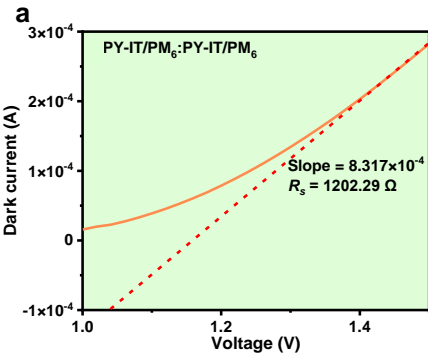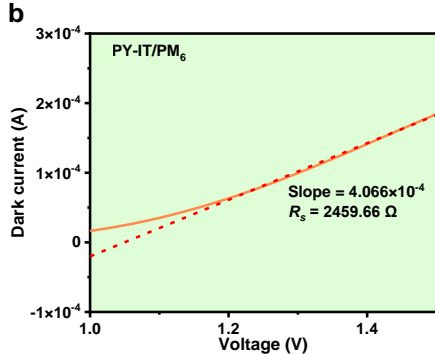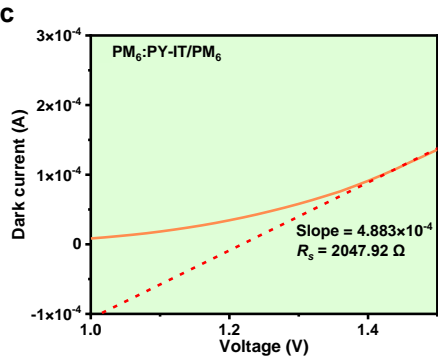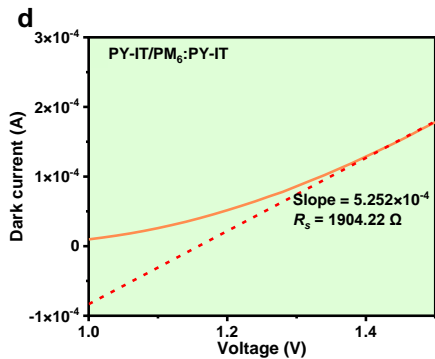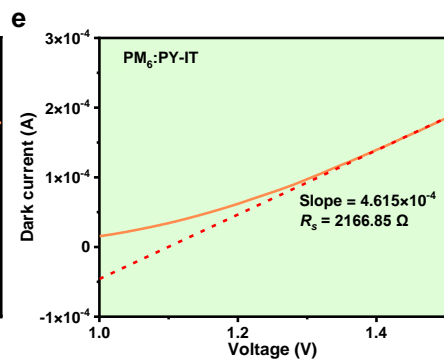

Supplement: Supplementary 1 — Texts S1 to S11 Tables S1 to S5 Figs. S1 to S42 References [56–105] [file research.0939.f1.zip › Figure S13.pdf]

**a**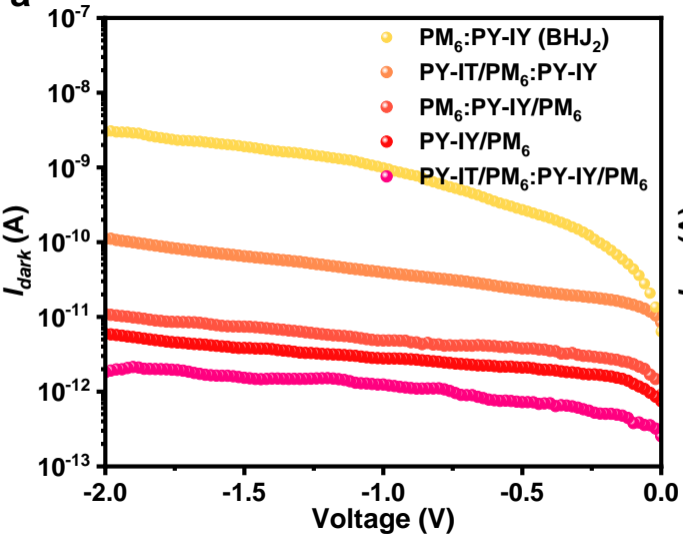**b**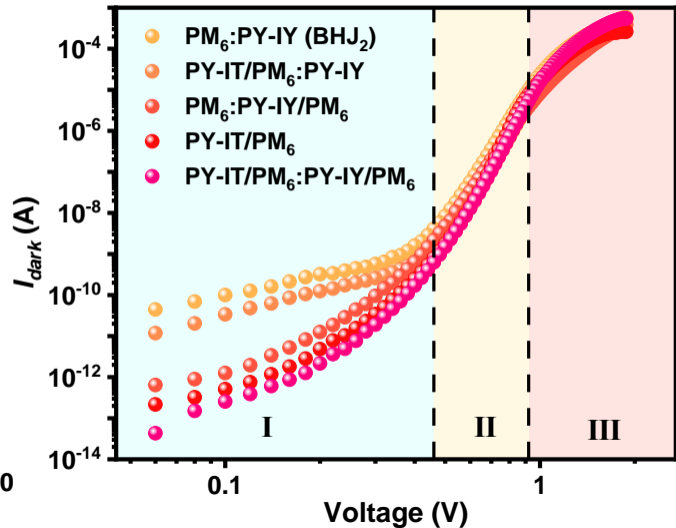

Supplement: Supplementary 1 — Texts S1 to S11 Tables S1 to S5 Figs. S1 to S42 References [56–105] [file research.0939.f1.zip › Figure S14.pdf]

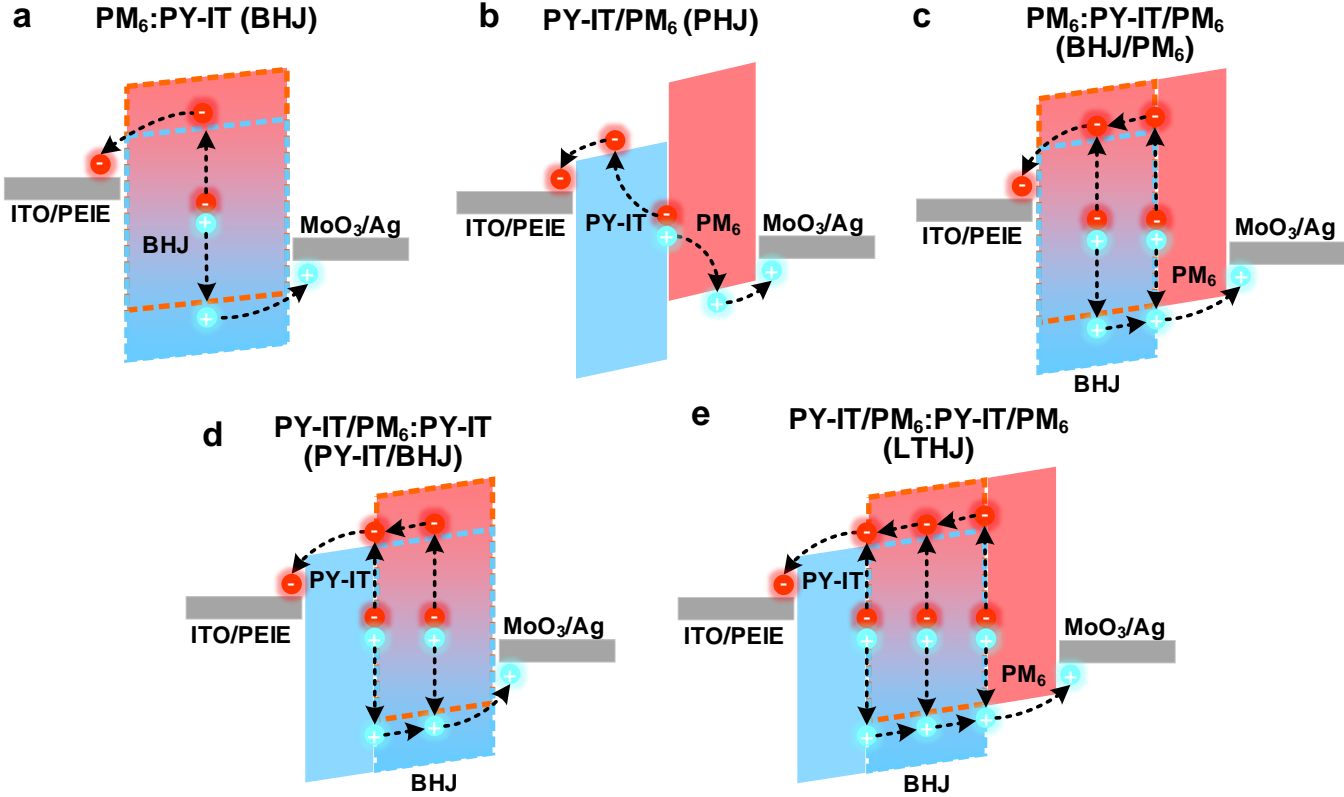

Supplement: Supplementary 1 — Texts S1 to S11 Tables S1 to S5 Figs. S1 to S42 References [56–105] [file research.0939.f1.zip › Figure S15.pdf]

**a**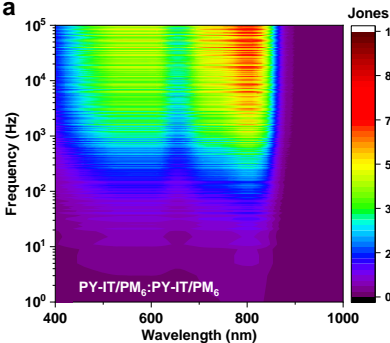**b**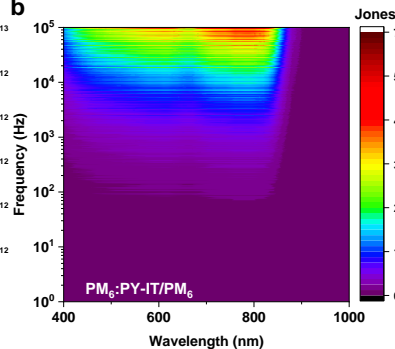**c**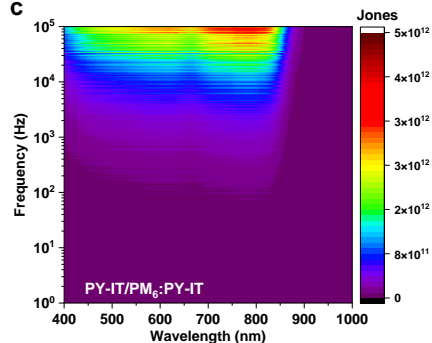**d**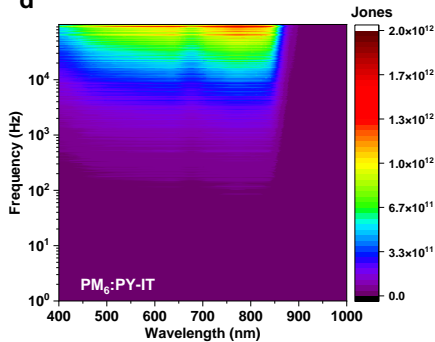**e**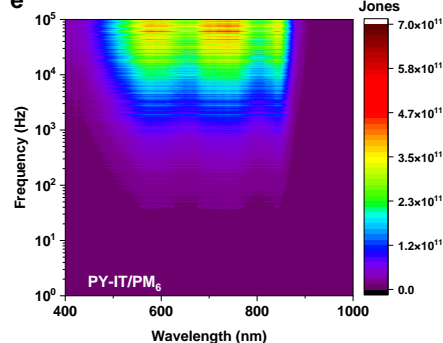

Supplement: Supplementary 1 — Texts S1 to S11 Tables S1 to S5 Figs. S1 to S42 References [56–105] [file research.0939.f1.zip › Figure S16.pdf]

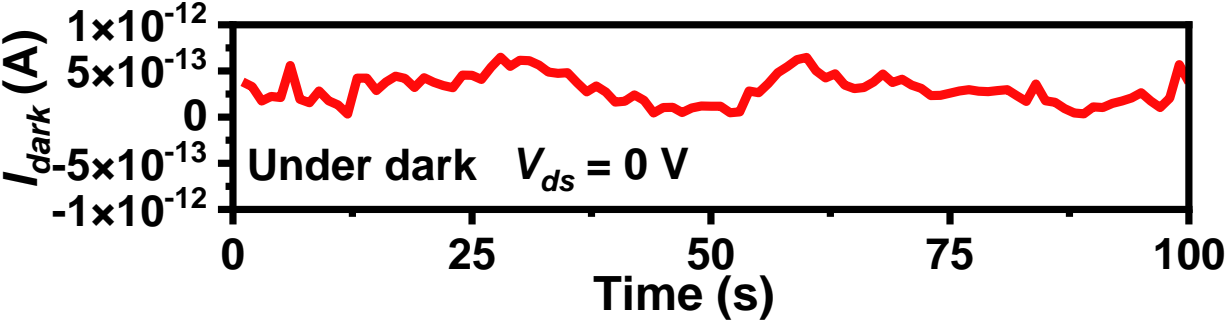

Supplement: Supplementary 1 — Texts S1 to S11 Tables S1 to S5 Figs. S1 to S42 References [56–105] [file research.0939.f1.zip › Figure S17.pdf]

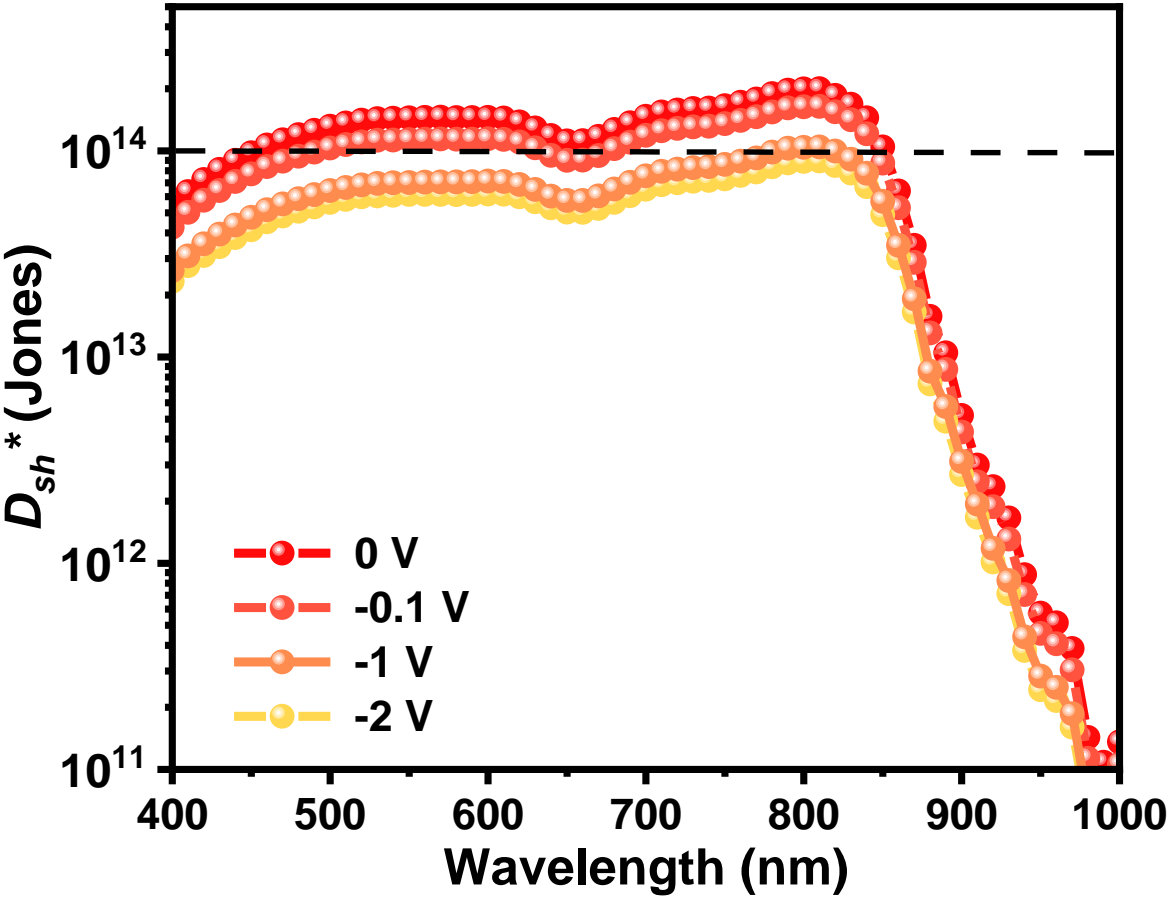

Supplement: Supplementary 1 — Texts S1 to S11 Tables S1 to S5 Figs. S1 to S42 References [56–105] [file research.0939.f1.zip › Figure S18.pdf]

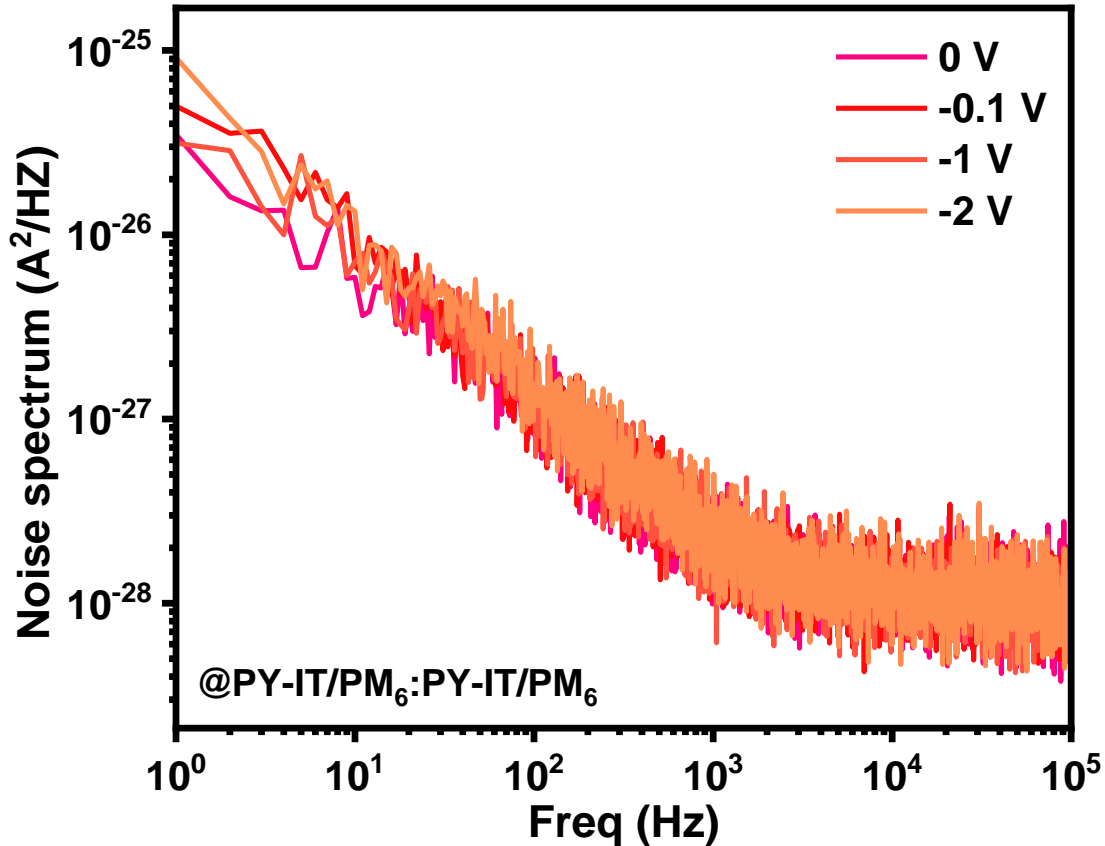

Supplement: Supplementary 1 — Texts S1 to S11 Tables S1 to S5 Figs. S1 to S42 References [56–105] [file research.0939.f1.zip › Figure S19.pdf]

**a**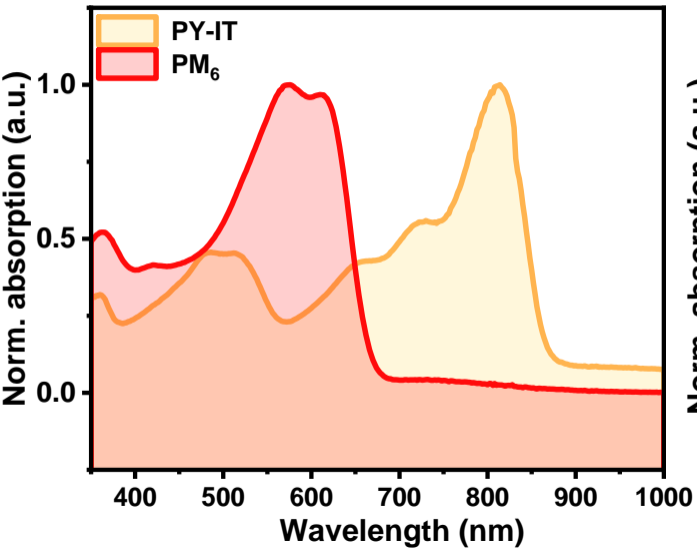**b**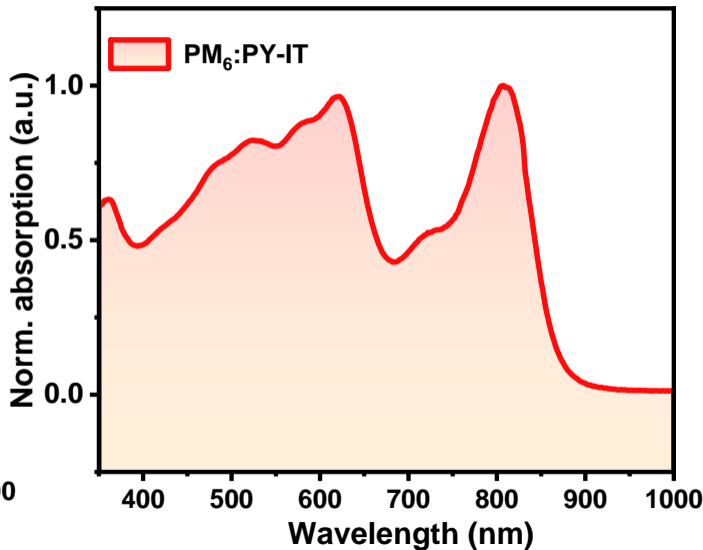

Supplement: Supplementary 1 — Texts S1 to S11 Tables S1 to S5 Figs. S1 to S42 References [56–105] [file research.0939.f1.zip › Figure S2.pdf]

Jones

$1 \times 10^{13}$

$9 \times 10^{12}$

$7 \times 10^{12}$

$5 \times 10^{12}$

$3 \times 10^{12}$

$2 \times 10^{12}$

0

$10^5$

$10^4$

$10^3$

$10^2$

$10^1$

$10^0$

Frequency (Hz)

$V_{ds} = -1 \text{ V}$

400

600

800

1000

Wavelength (nm)

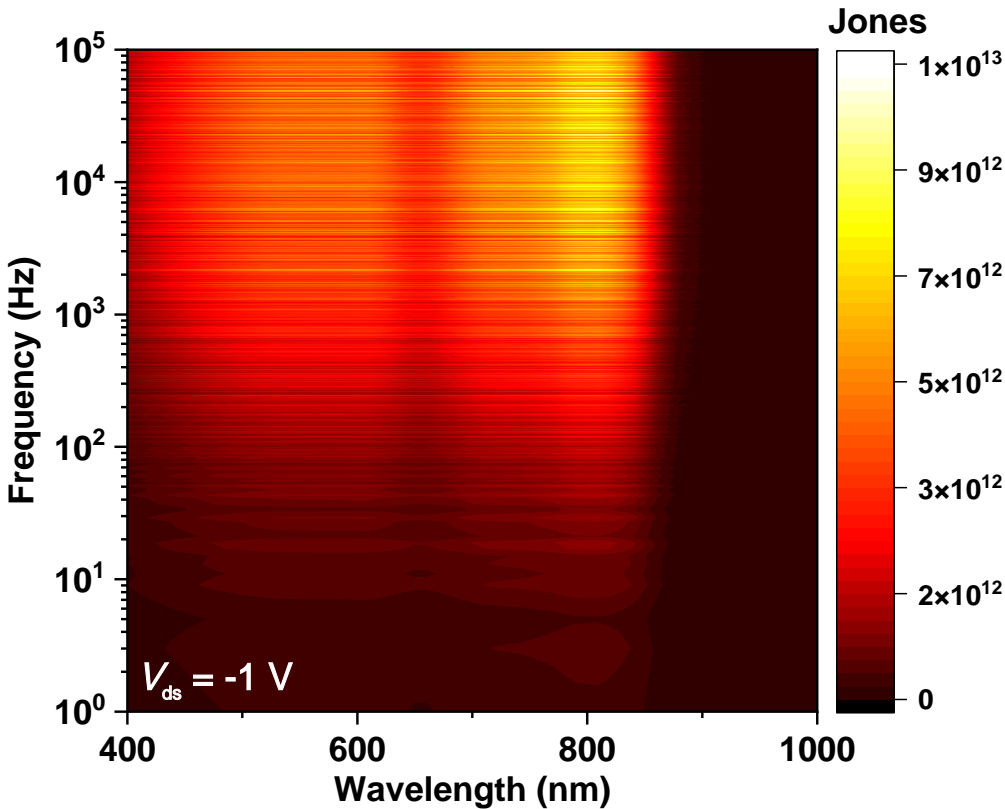

Supplement: Supplementary 1 — Texts S1 to S11 Tables S1 to S5 Figs. S1 to S42 References [56–105] [file research.0939.f1.zip › Figure S20.pdf]

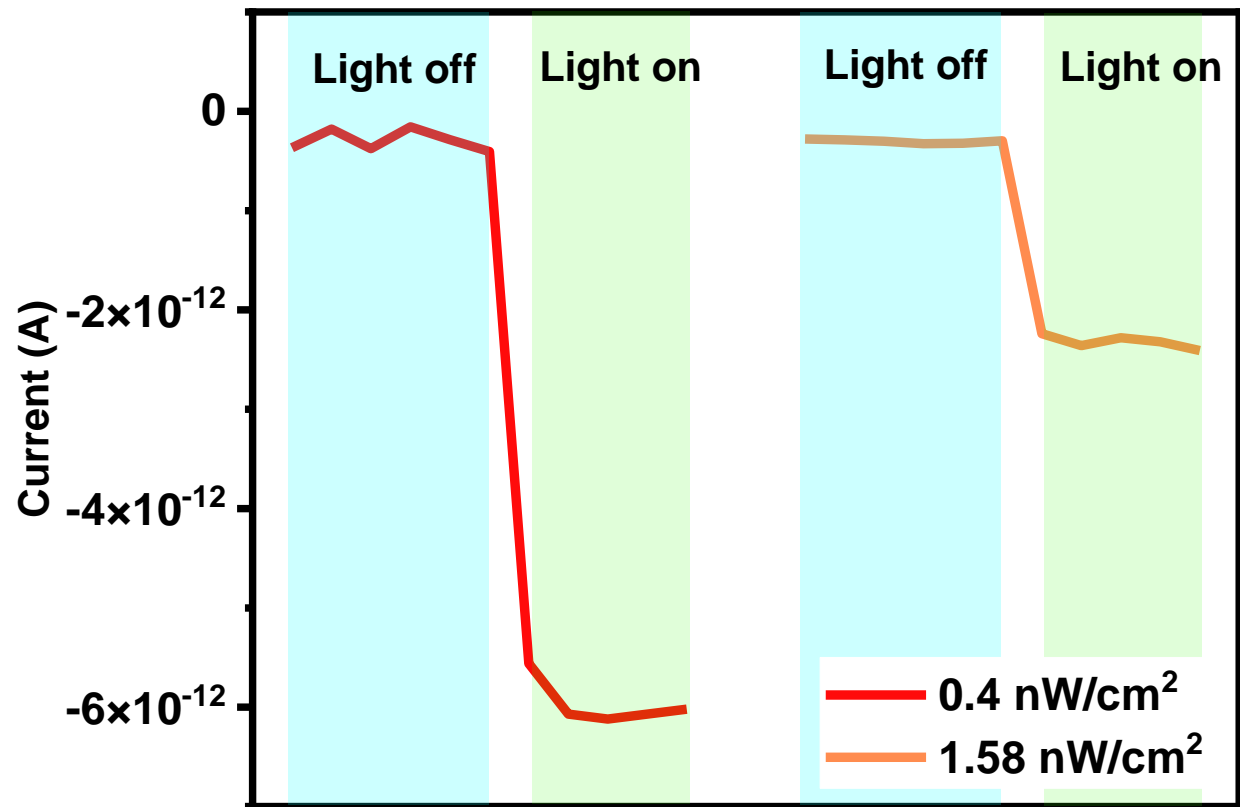

Supplement: Supplementary 1 — Texts S1 to S11 Tables S1 to S5 Figs. S1 to S42 References [56–105] [file research.0939.f1.zip › Figure S21.pdf]

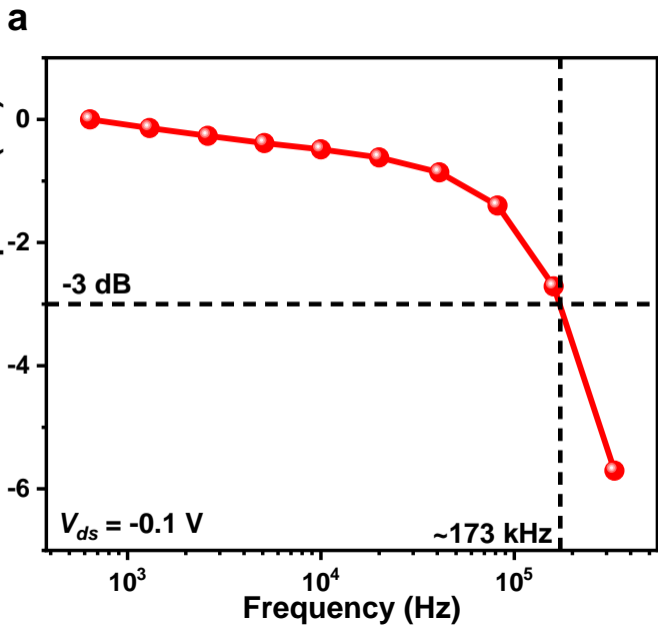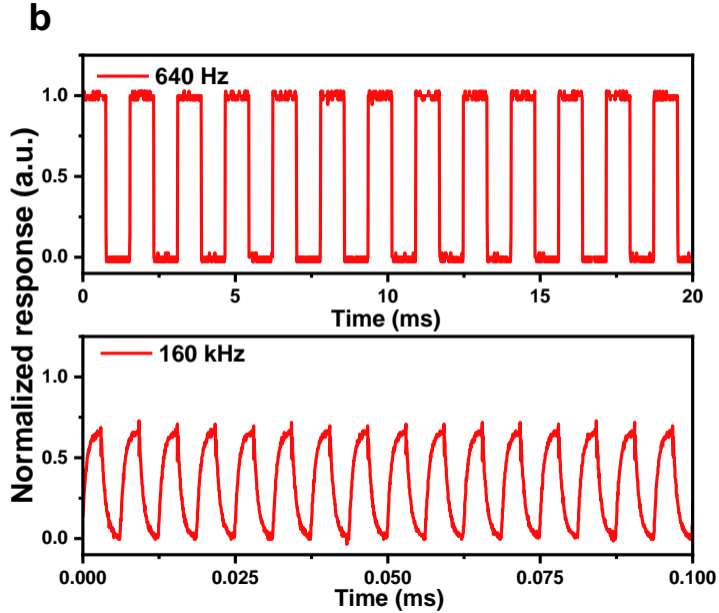

Supplement: Supplementary 1 — Texts S1 to S11 Tables S1 to S5 Figs. S1 to S42 References [56–105] [file research.0939.f1.zip › Figure S22.pdf]

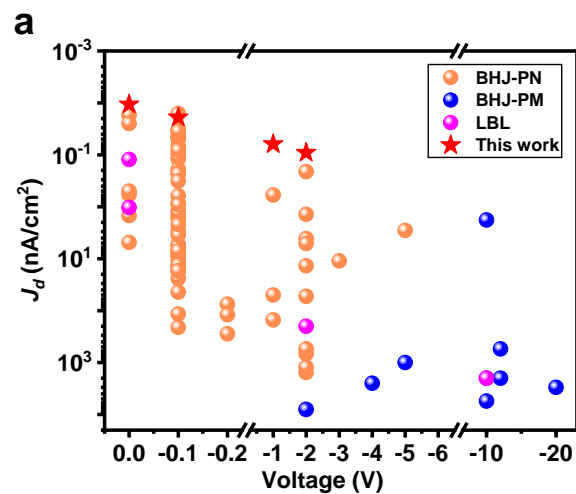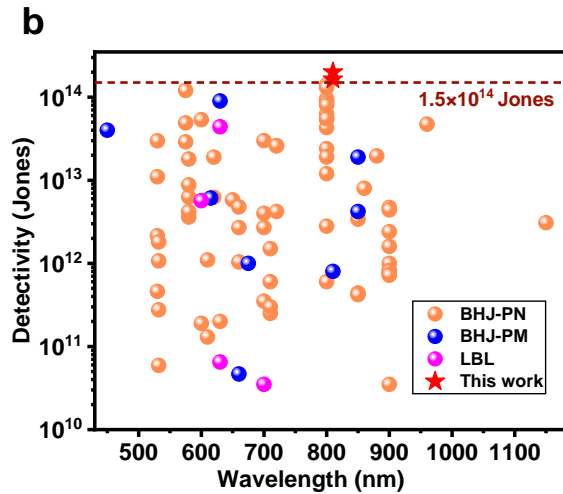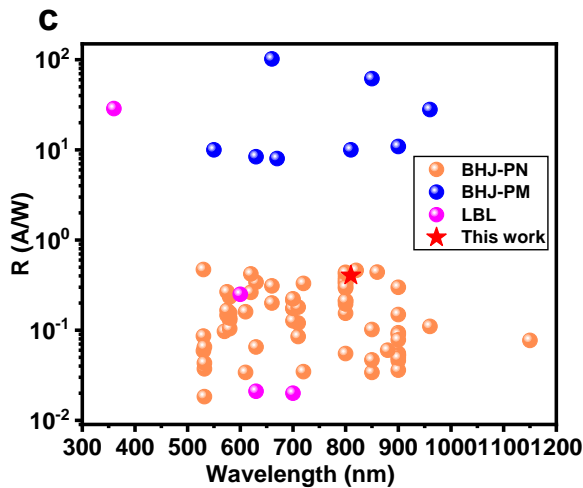

Supplement: Supplementary 1 — Texts S1 to S11 Tables S1 to S5 Figs. S1 to S42 References [56–105] [file research.0939.f1.zip › Figure S23.pdf]

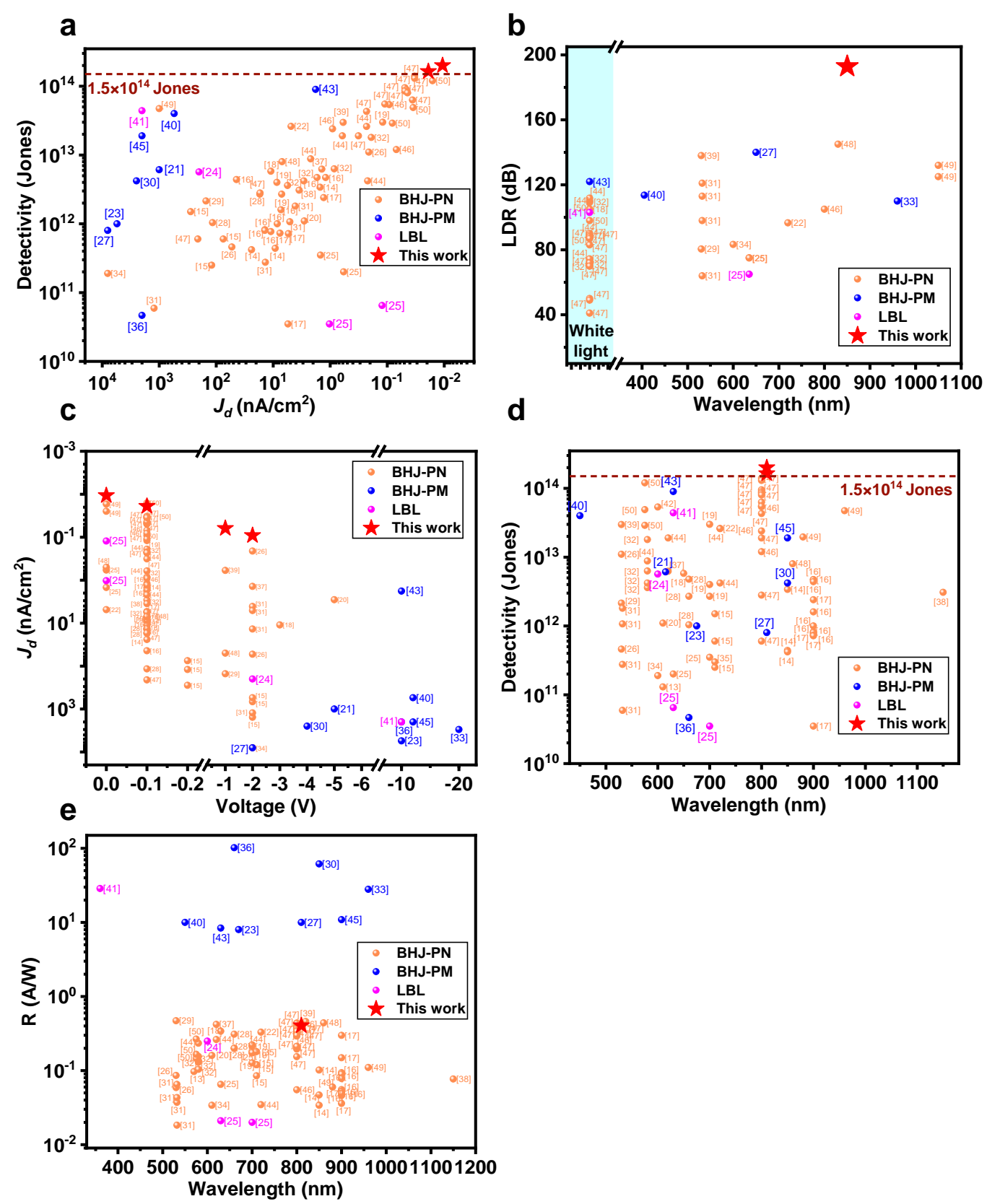

Supplement: Supplementary 1 — Texts S1 to S11 Tables S1 to S5 Figs. S1 to S42 References [56–105] [file research.0939.f1.zip › Figure S24.pdf]

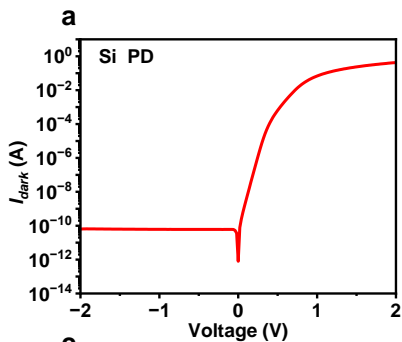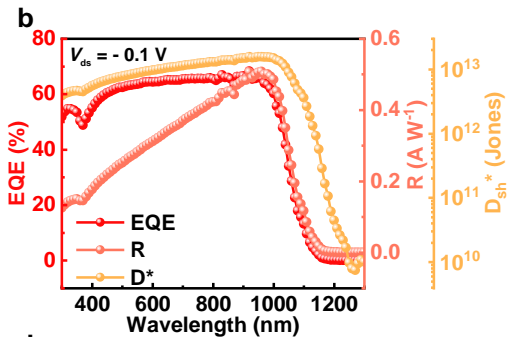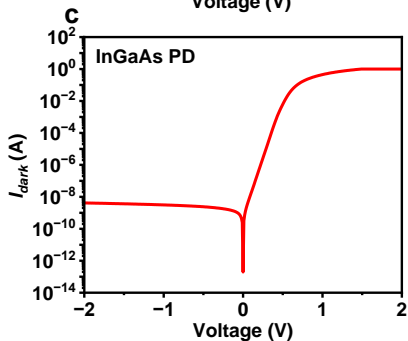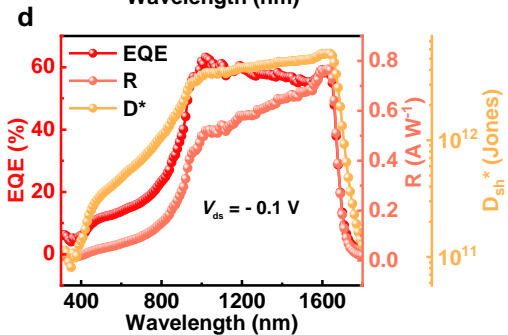

Supplement: Supplementary 1 — Texts S1 to S11 Tables S1 to S5 Figs. S1 to S42 References [56–105] [file research.0939.f1.zip › Figure S25.pdf]

**a**Variance of  $I_{light}$ 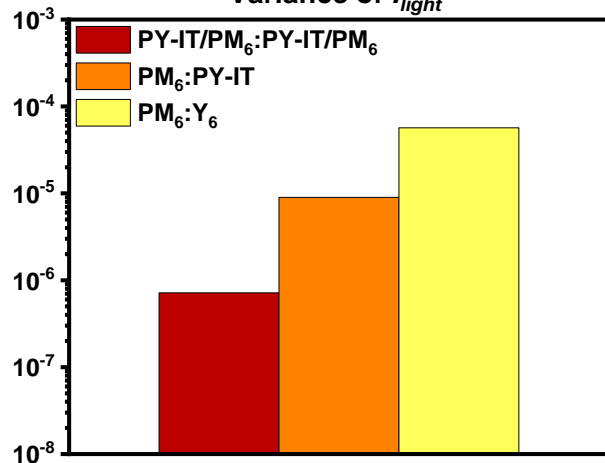**b**Variance of  $I_{dark}$ 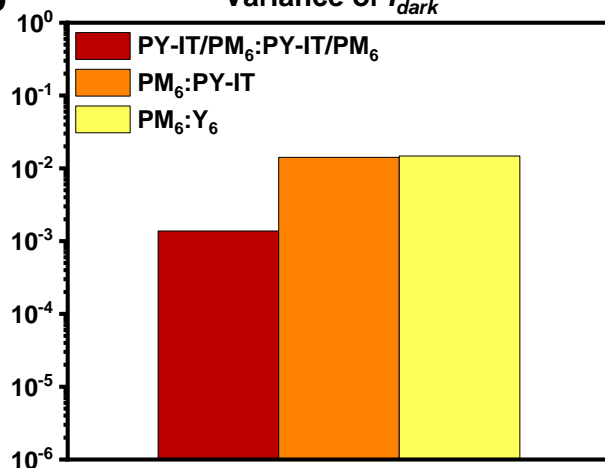**c**Maximum offset of  $I_{light}$ 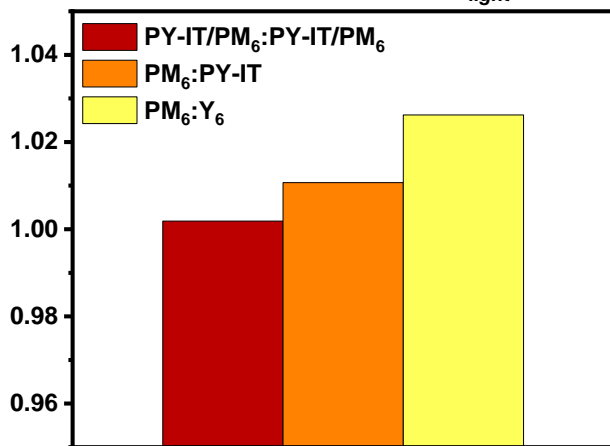**d**Maximum offset of  $I_{dark}$ 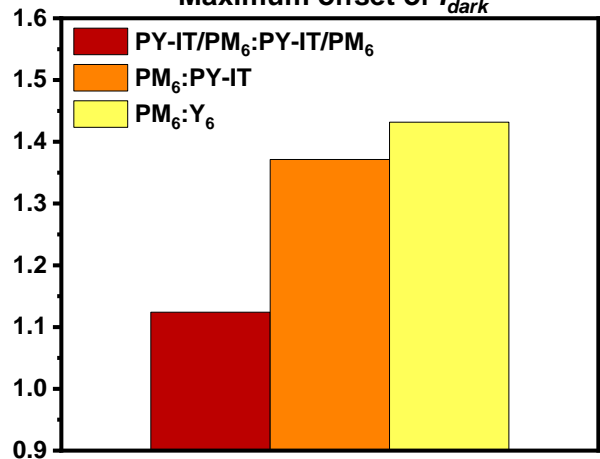

Supplement: Supplementary 1 — Texts S1 to S11 Tables S1 to S5 Figs. S1 to S42 References [56–105] [file research.0939.f1.zip › Figure S26.pdf]

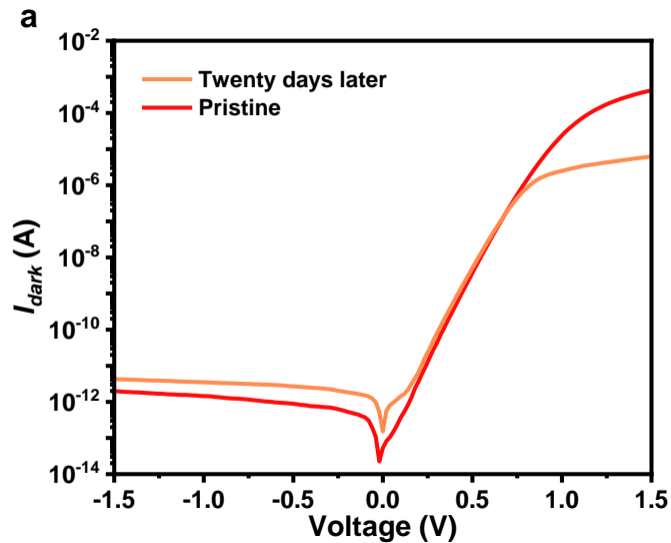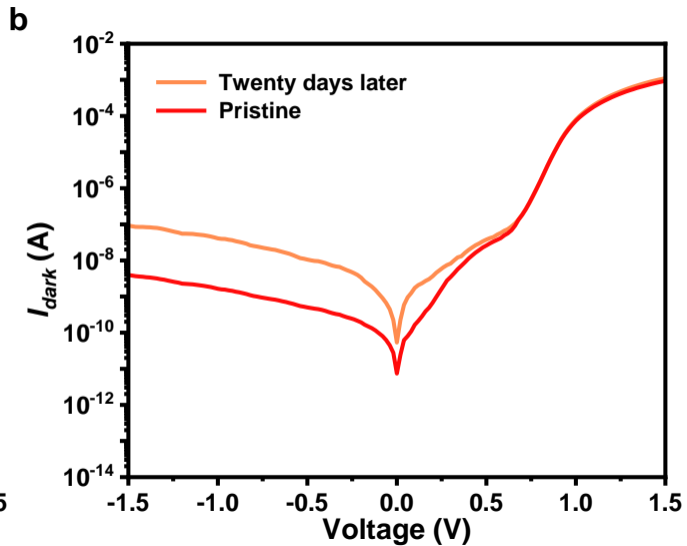

Supplement: Supplementary 1 — Texts S1 to S11 Tables S1 to S5 Figs. S1 to S42 References [56–105] [file research.0939.f1.zip › Figure S27.pdf]

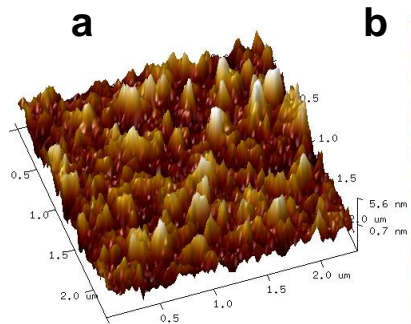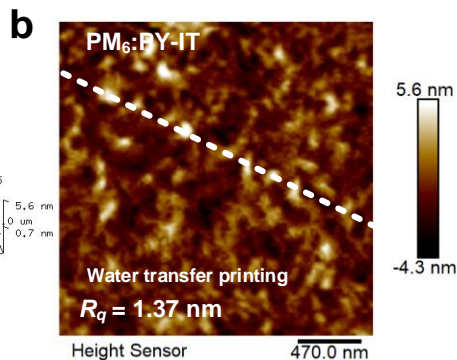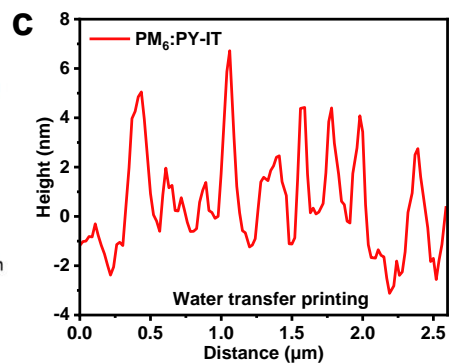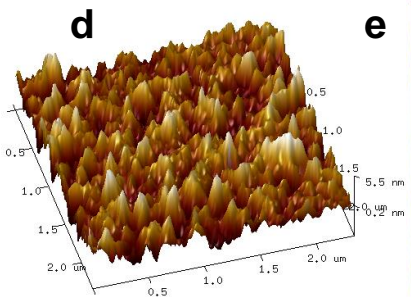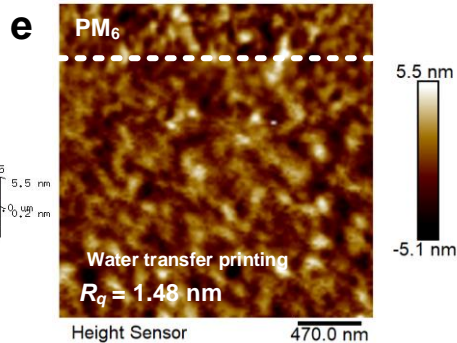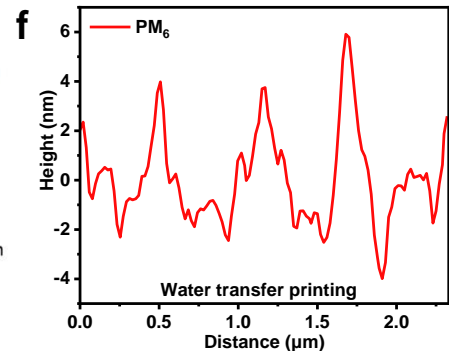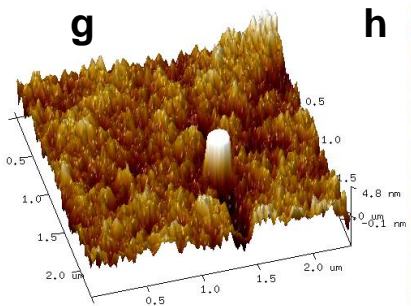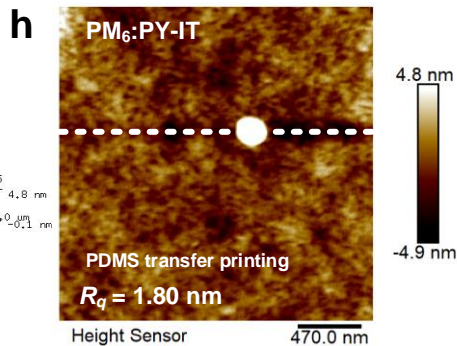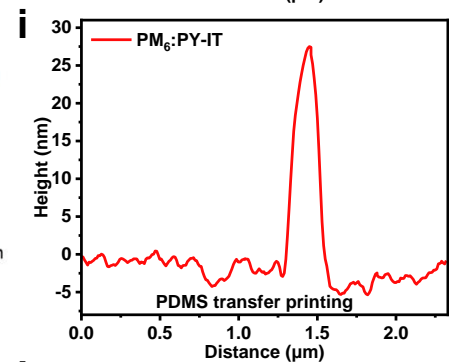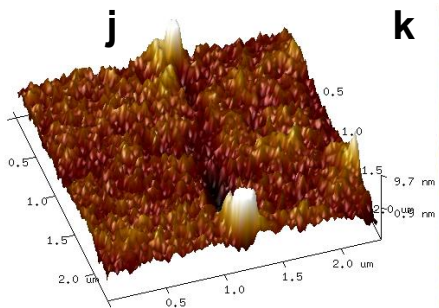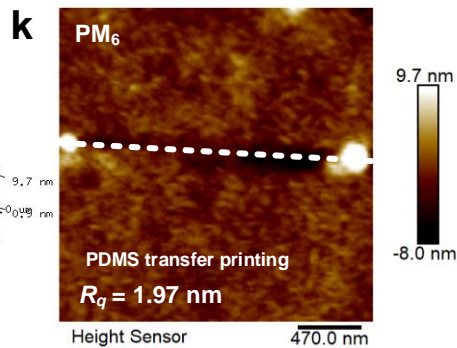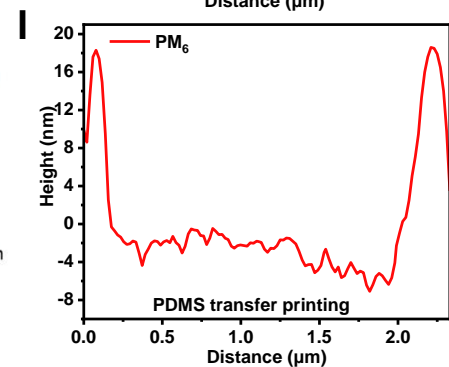

Supplement: Supplementary 1 — Texts S1 to S11 Tables S1 to S5 Figs. S1 to S42 References [56–105] [file research.0939.f1.zip › Figure S28.pdf]

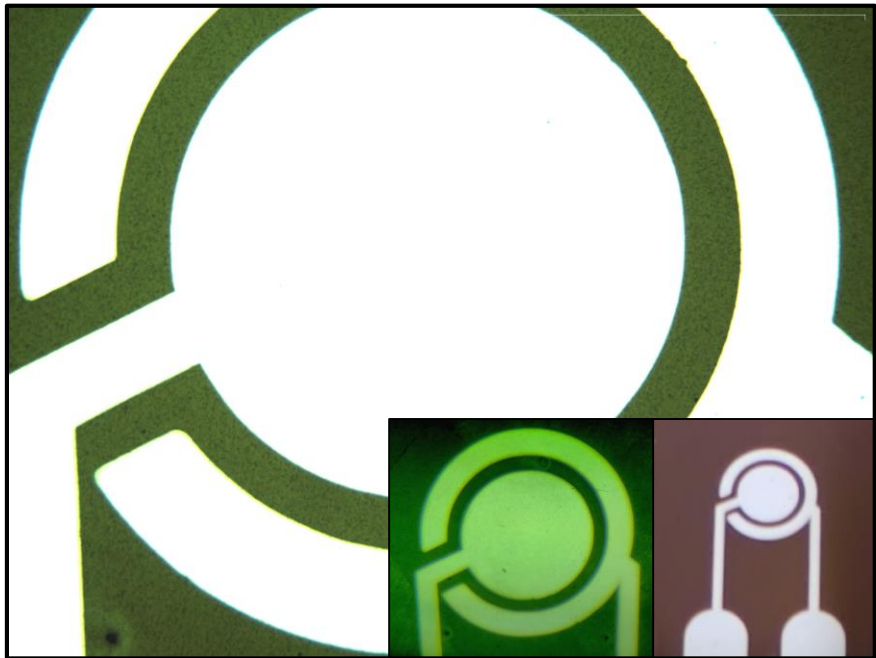

Supplement: Supplementary 1 — Texts S1 to S11 Tables S1 to S5 Figs. S1 to S42 References [56–105] [file research.0939.f1.zip › Figure S29.pdf]

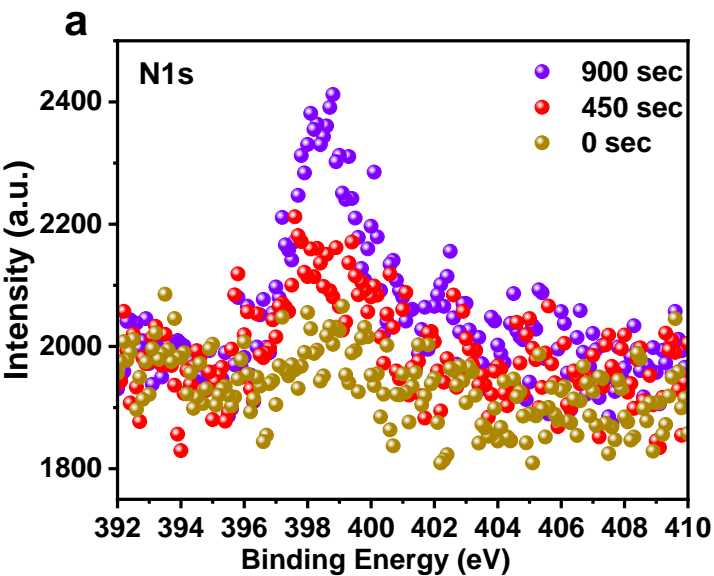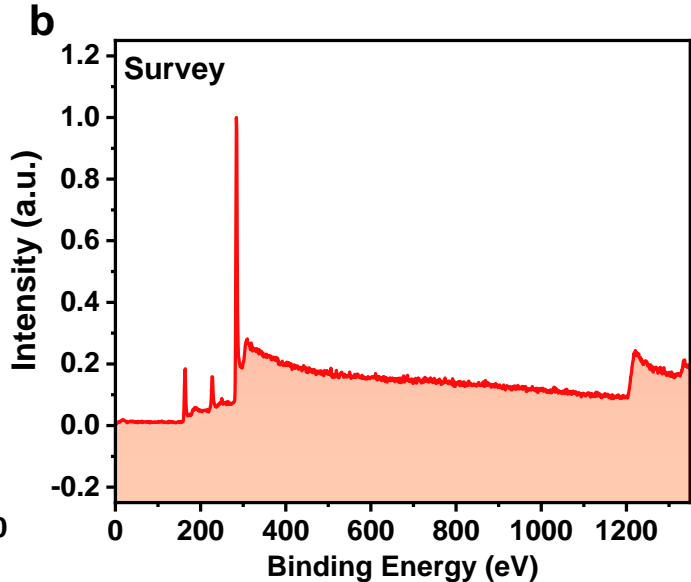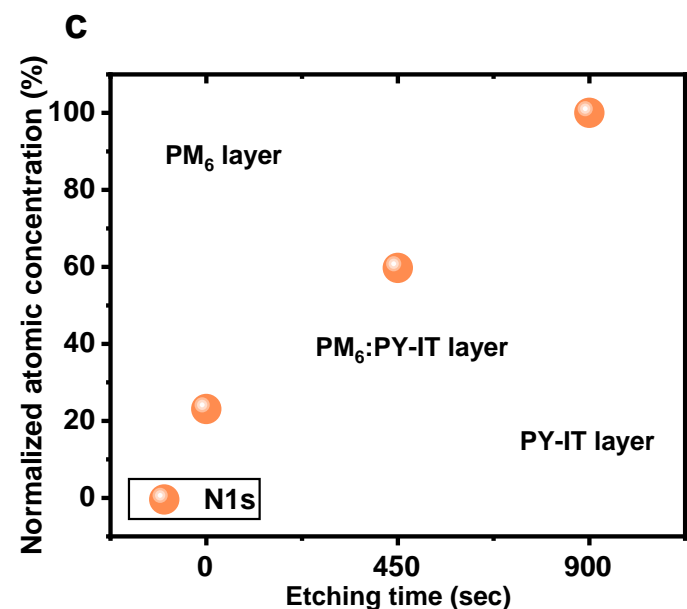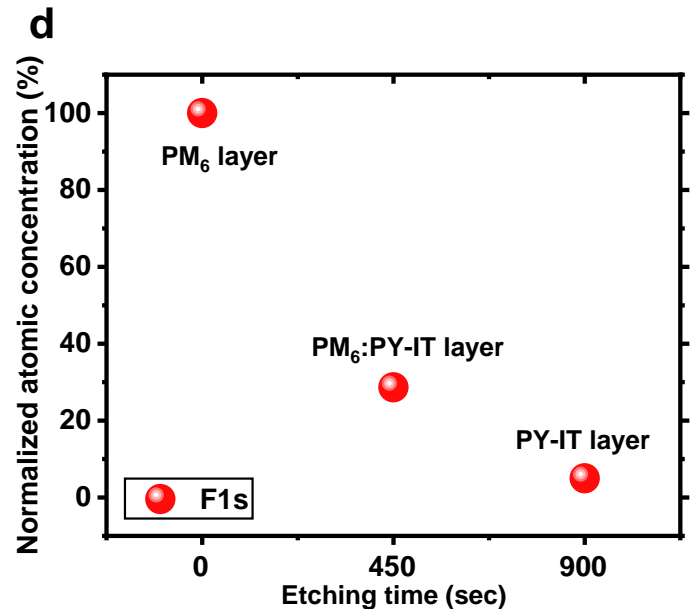

Supplement: Supplementary 1 — Texts S1 to S11 Tables S1 to S5 Figs. S1 to S42 References [56–105] [file research.0939.f1.zip › Figure S3.pdf]

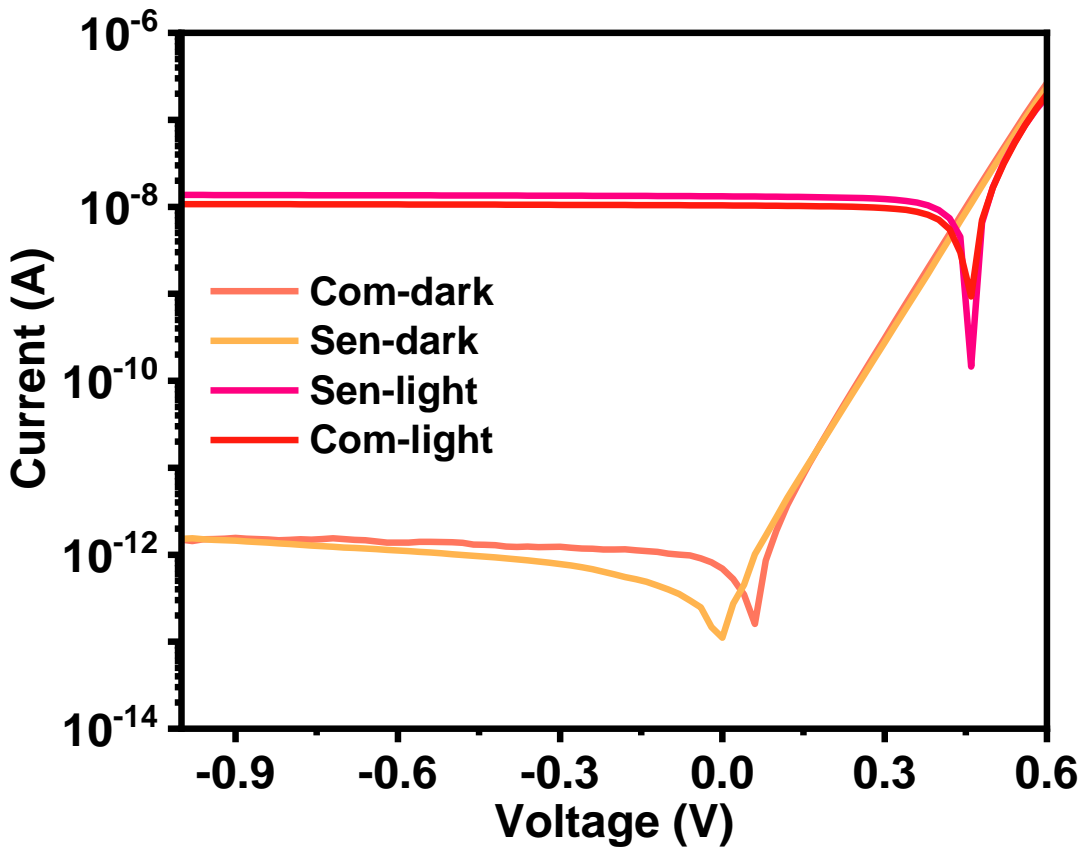

Supplement: Supplementary 1 — Texts S1 to S11 Tables S1 to S5 Figs. S1 to S42 References [56–105] [file research.0939.f1.zip › Figure S30.pdf]

**a**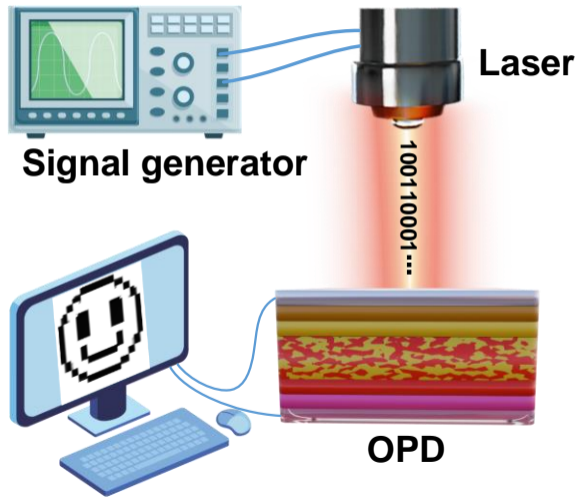**b**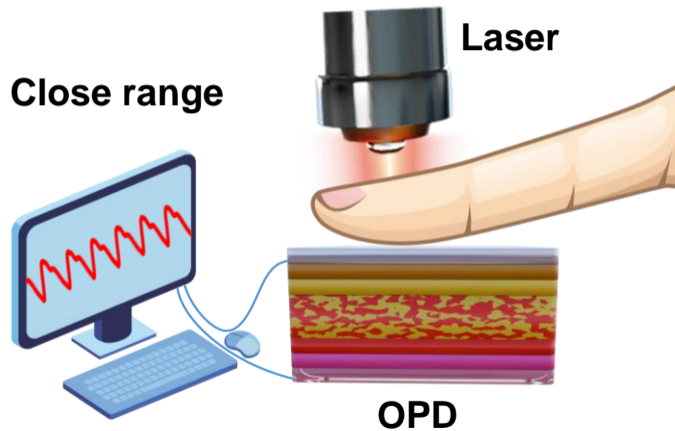

Supplement: Supplementary 1 — Texts S1 to S11 Tables S1 to S5 Figs. S1 to S42 References [56–105] [file research.0939.f1.zip › Figure S31.pdf]

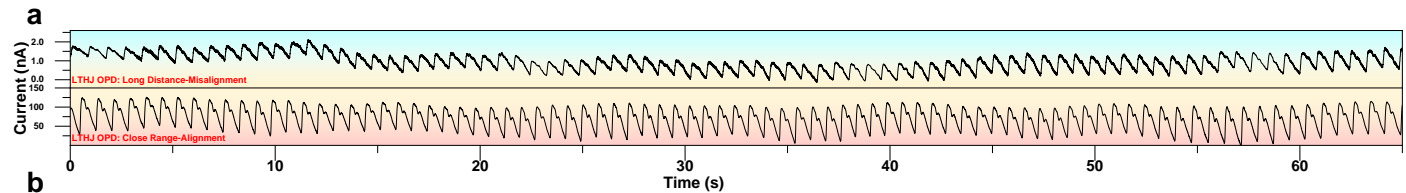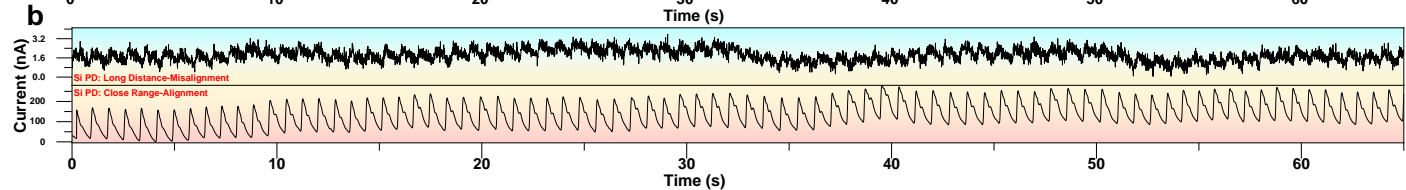

Supplement: Supplementary 1 — Texts S1 to S11 Tables S1 to S5 Figs. S1 to S42 References [56–105] [file research.0939.f1.zip › Figure S32.pdf]

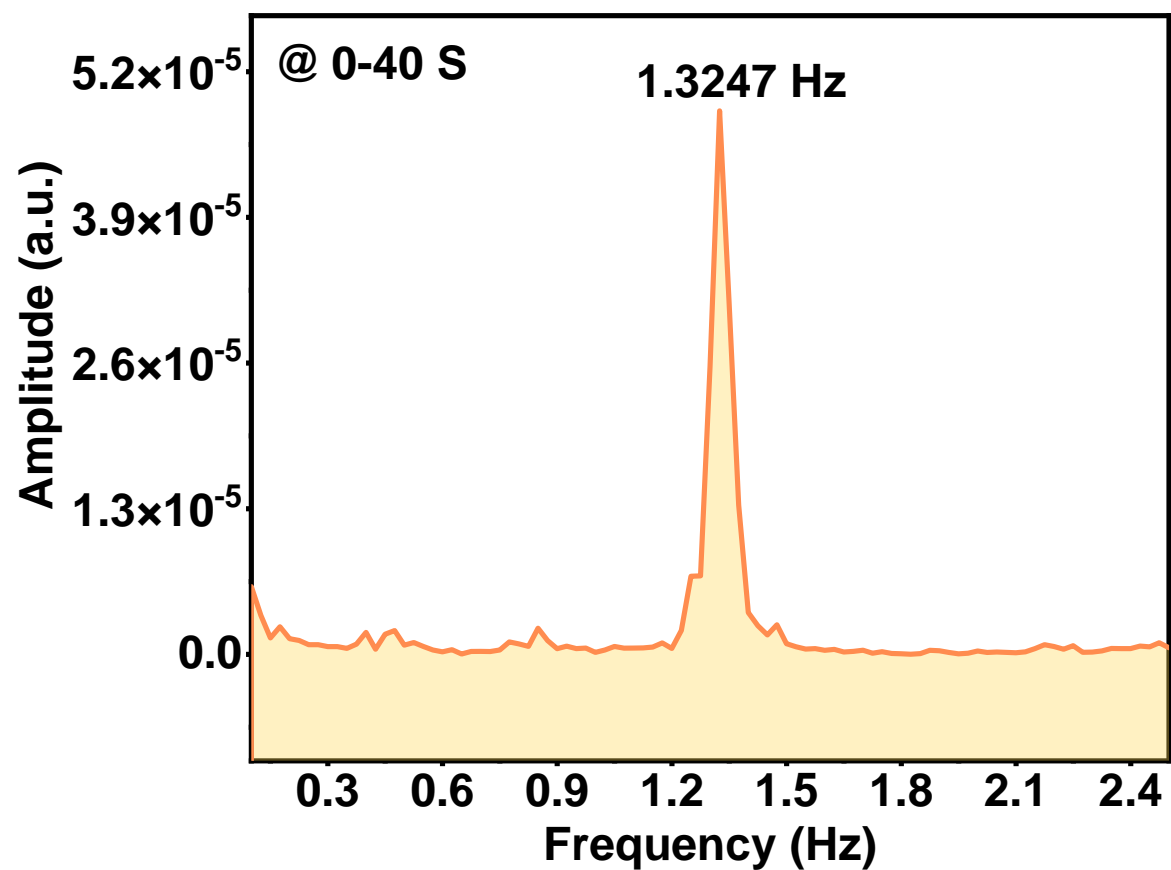

Supplement: Supplementary 1 — Texts S1 to S11 Tables S1 to S5 Figs. S1 to S42 References [56–105] [file research.0939.f1.zip › Figure S33.pdf]

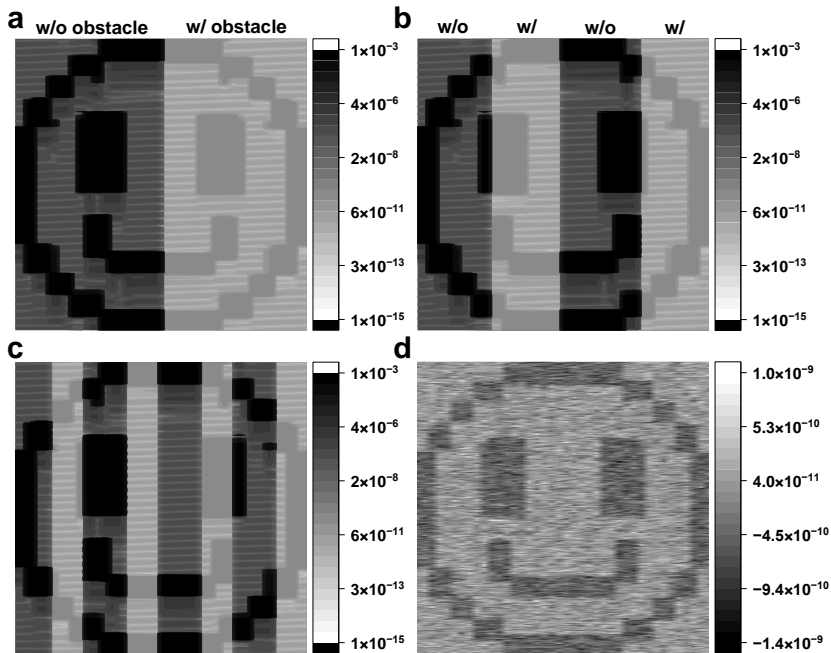

Supplement: Supplementary 1 — Texts S1 to S11 Tables S1 to S5 Figs. S1 to S42 References [56–105] [file research.0939.f1.zip › Figure S34.pdf]

**a**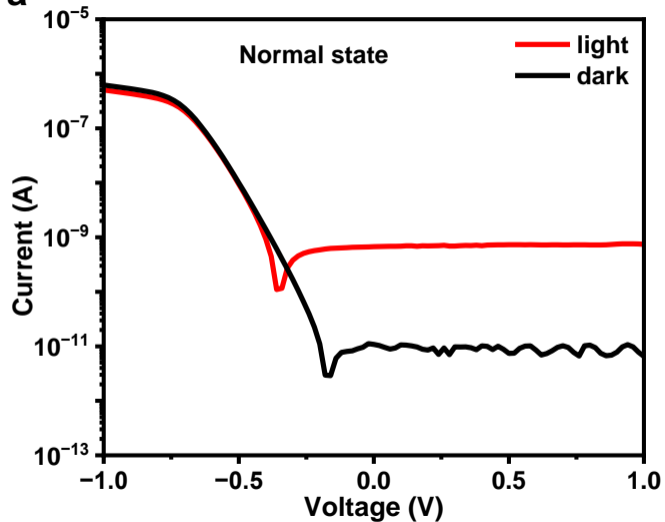**b**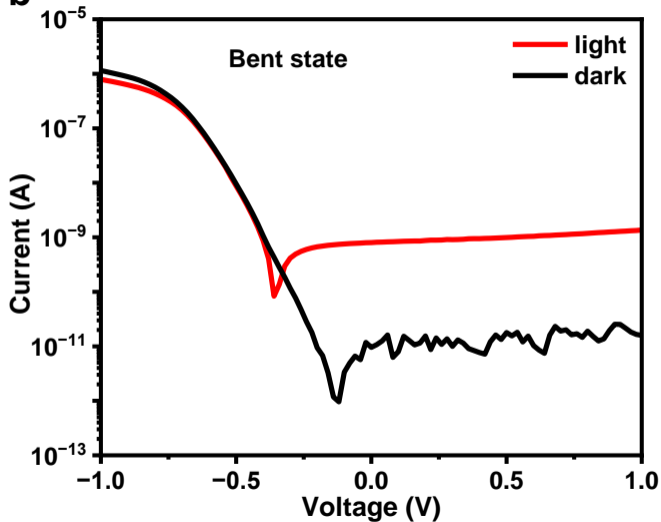

Supplement: Supplementary 1 — Texts S1 to S11 Tables S1 to S5 Figs. S1 to S42 References [56–105] [file research.0939.f1.zip › Figure S36.pdf]

**a**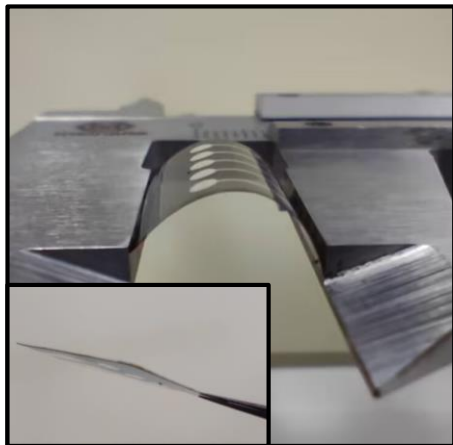**b**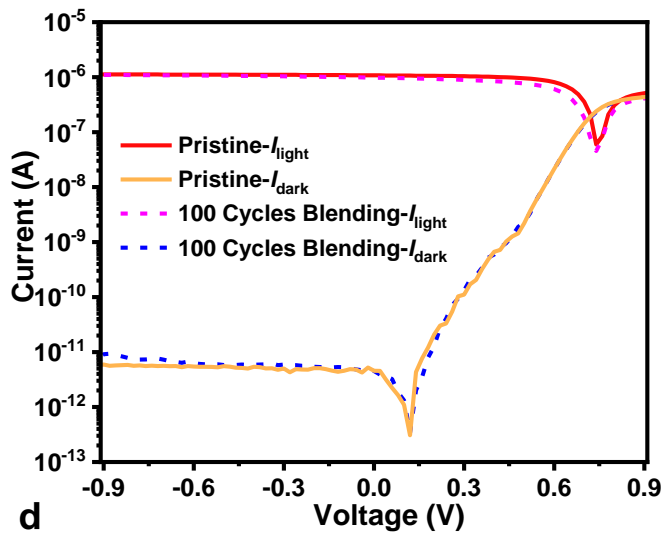**c**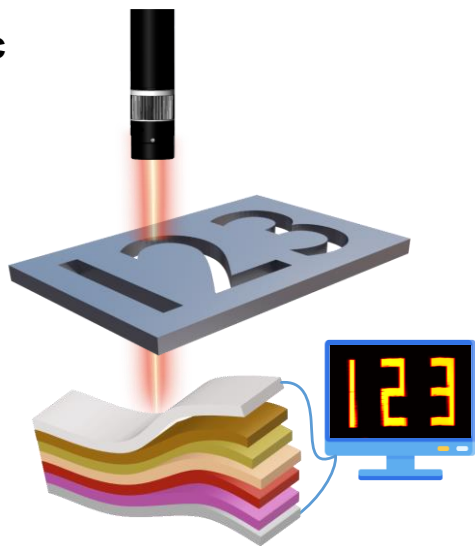**d**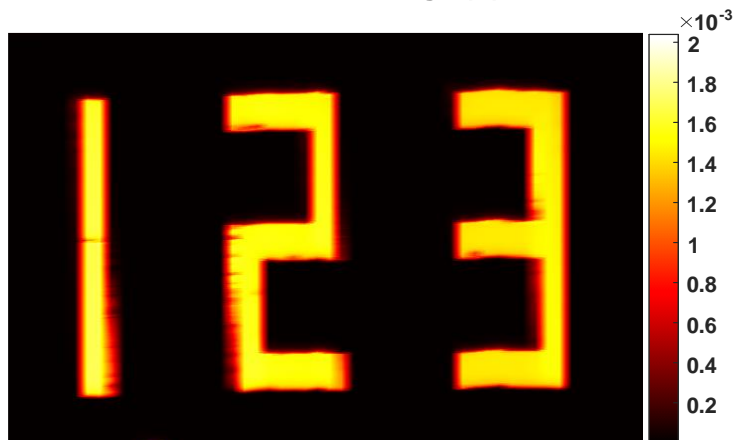

Supplement: Supplementary 1 — Texts S1 to S11 Tables S1 to S5 Figs. S1 to S42 References [56–105] [file research.0939.f1.zip › Figure S37.pdf]

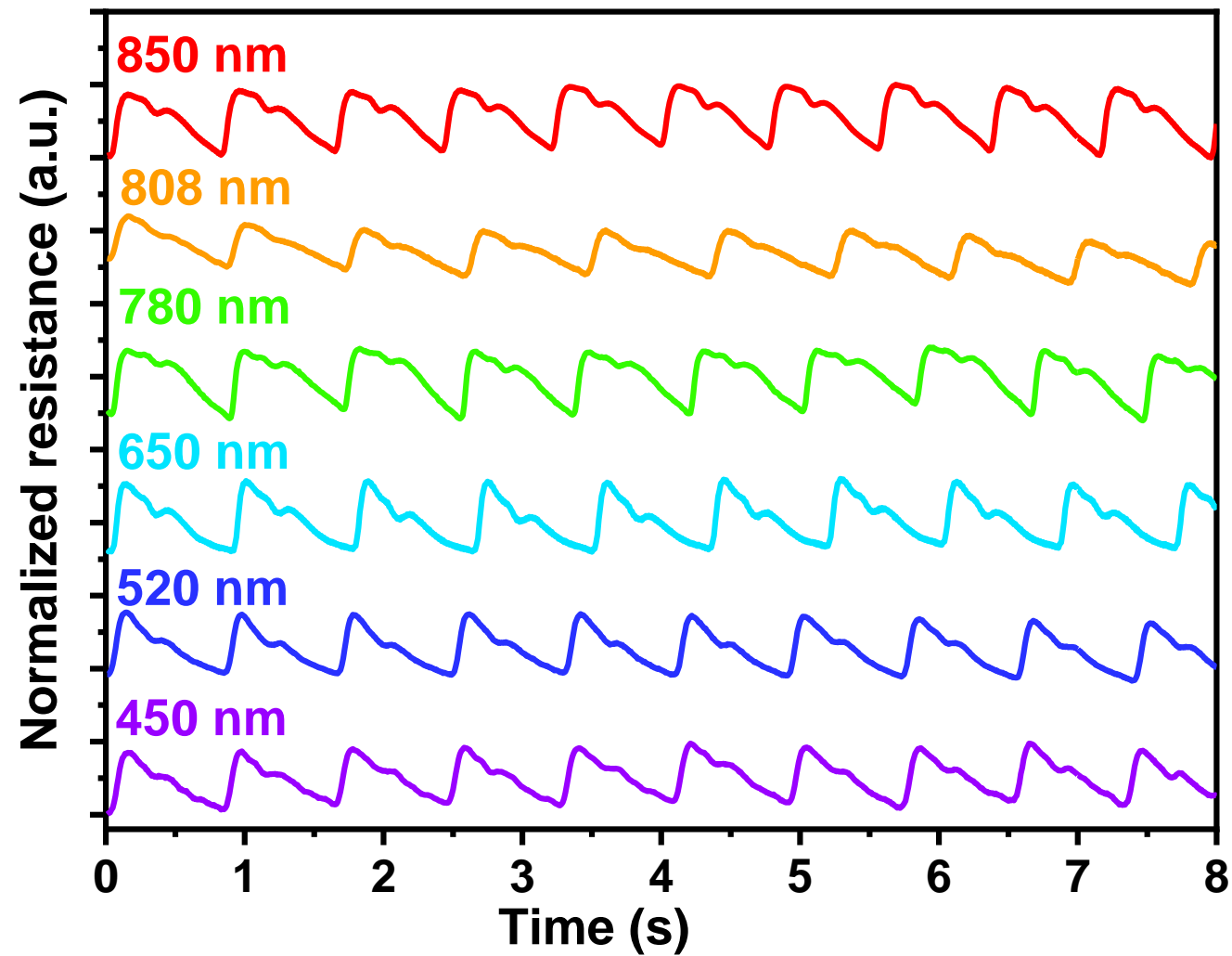

Supplement: Supplementary 1 — Texts S1 to S11 Tables S1 to S5 Figs. S1 to S42 References [56–105] [file research.0939.f1.zip › Figure S38.pdf]

**a**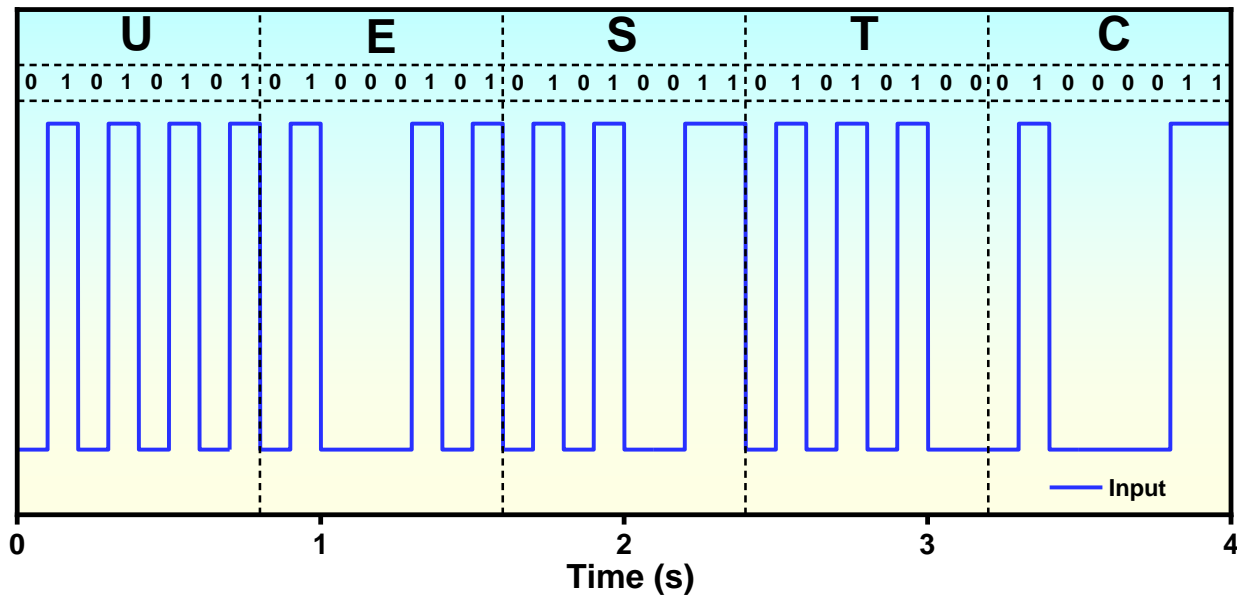**c**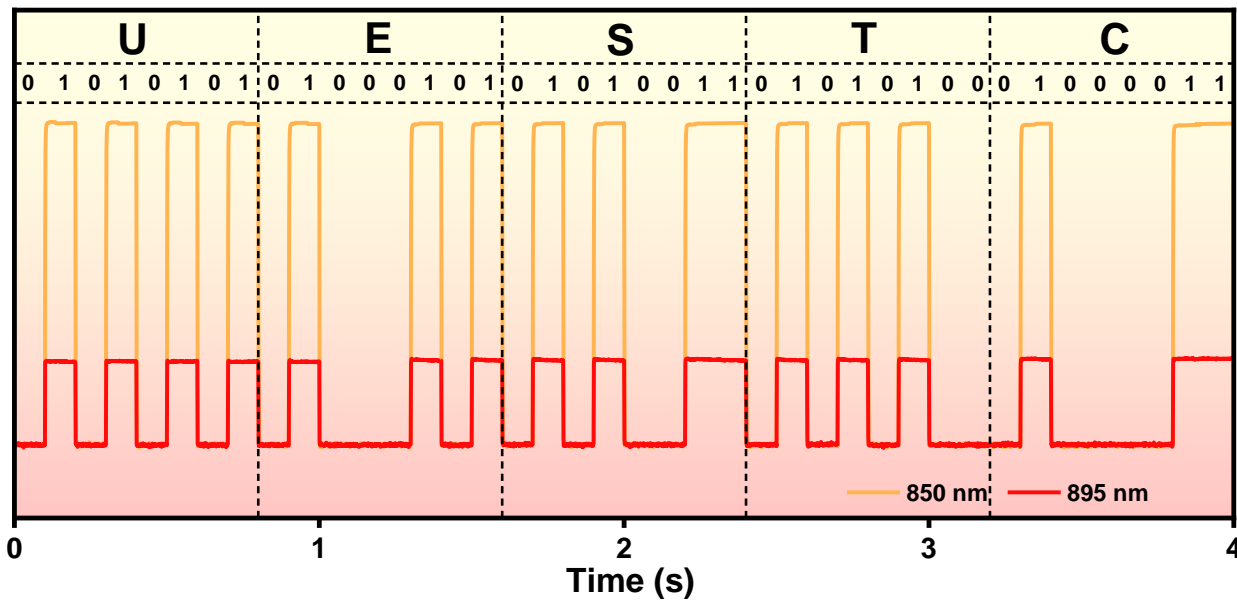

Supplement: Supplementary 1 — Texts S1 to S11 Tables S1 to S5 Figs. S1 to S42 References [56–105] [file research.0939.f1.zip › Figure S39.pdf]

**a**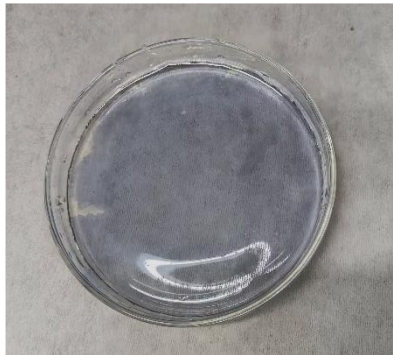**b**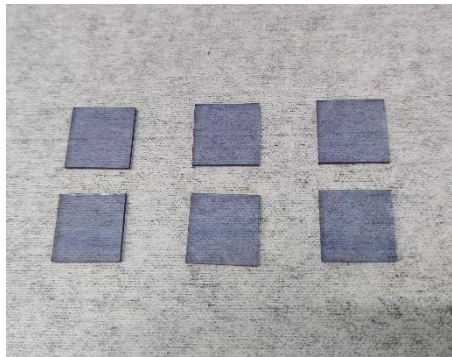**c**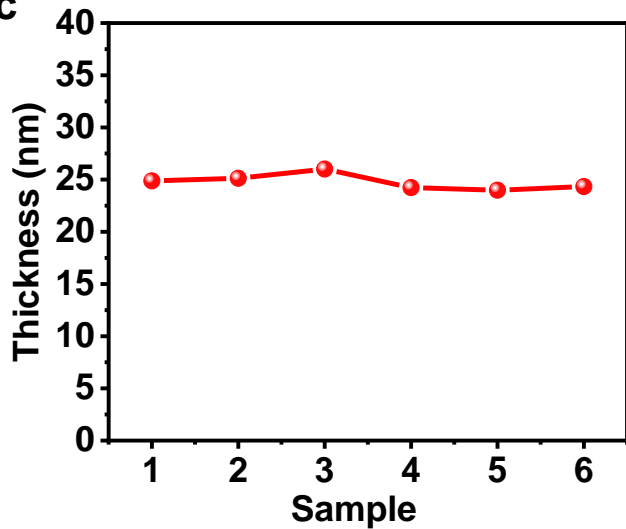

Supplement: Supplementary 1 — Texts S1 to S11 Tables S1 to S5 Figs. S1 to S42 References [56–105] [file research.0939.f1.zip › Figure S4.pdf]

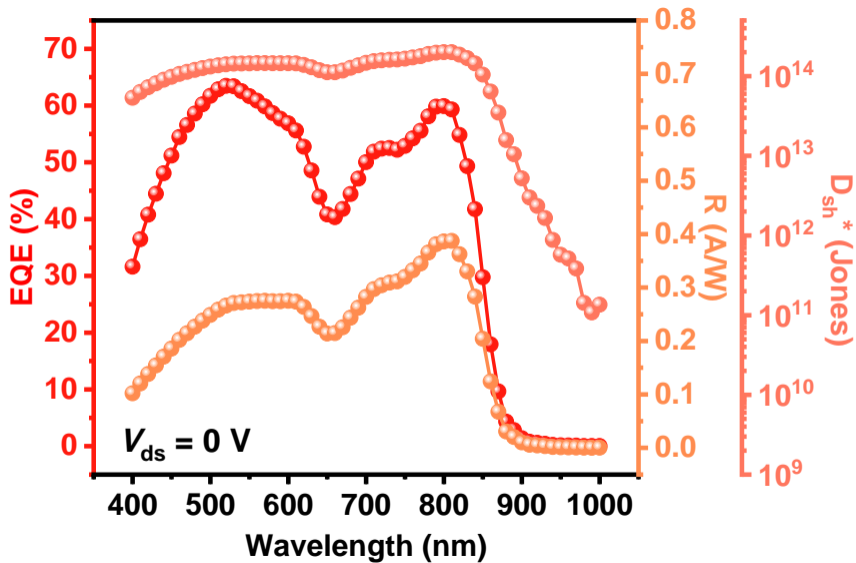

Supplement: Supplementary 1 — Texts S1 to S11 Tables S1 to S5 Figs. S1 to S42 References [56–105] [file research.0939.f1.zip › Figure S40.pdf]

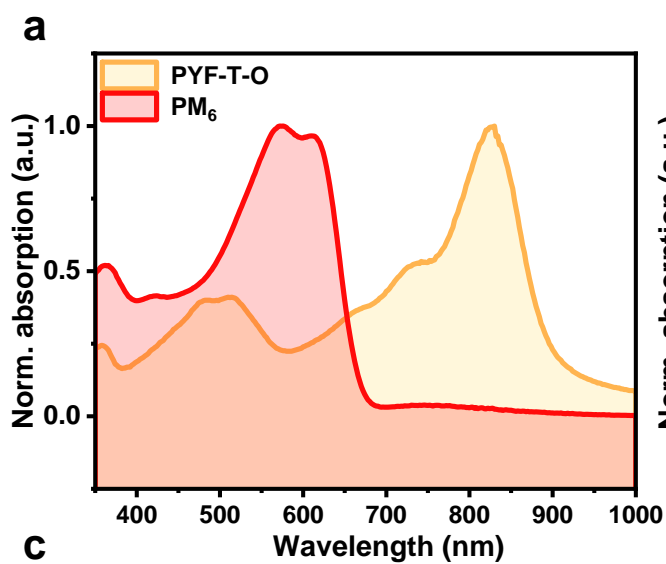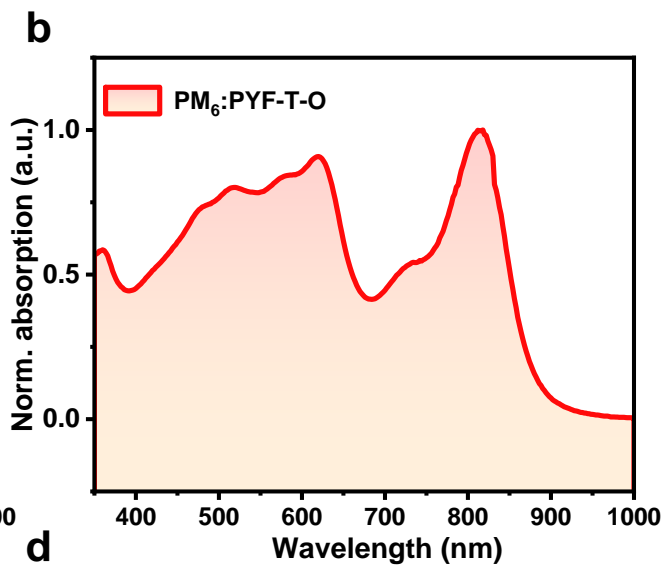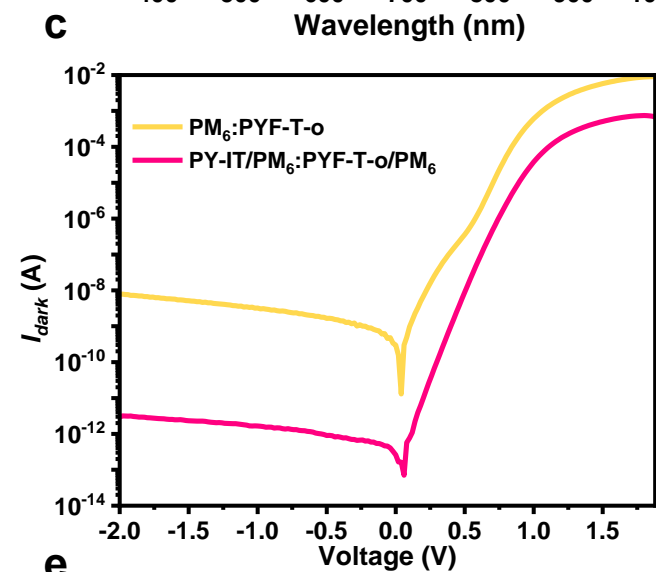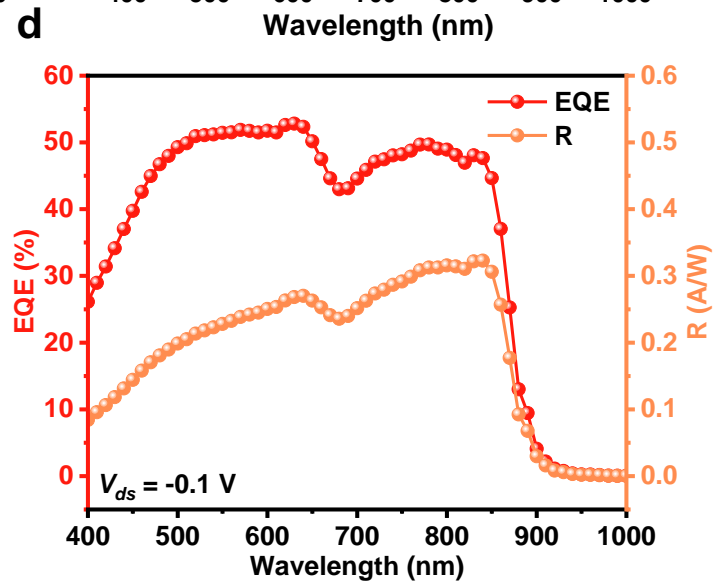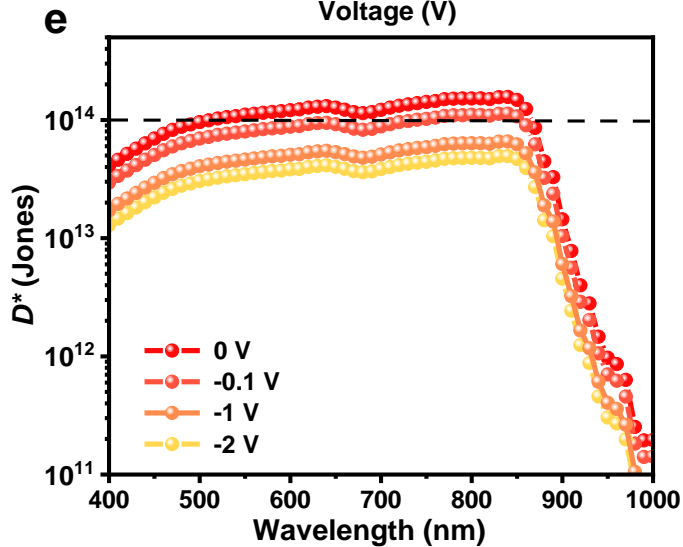

Supplement: Supplementary 1 — Texts S1 to S11 Tables S1 to S5 Figs. S1 to S42 References [56–105] [file research.0939.f1.zip › Figure S41.pdf]

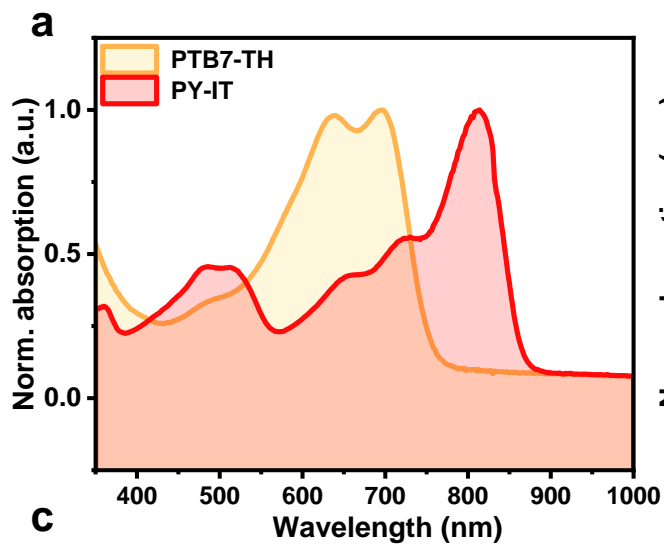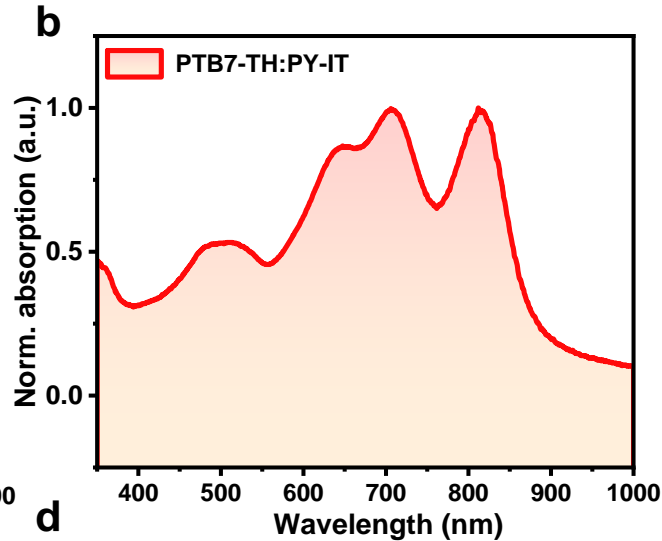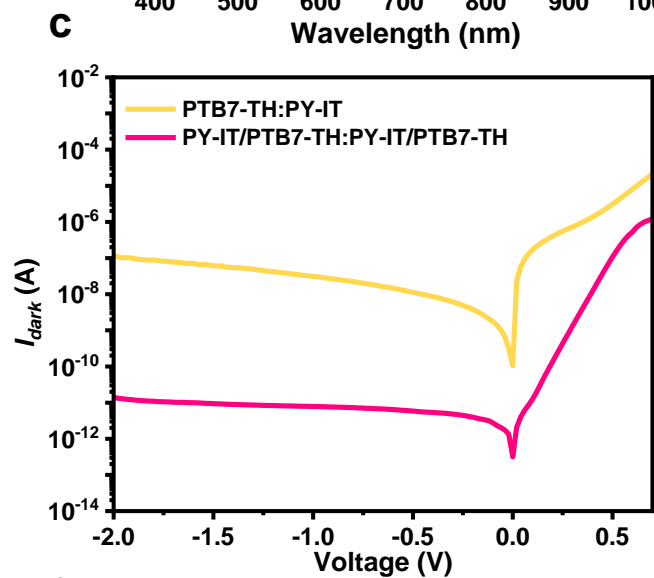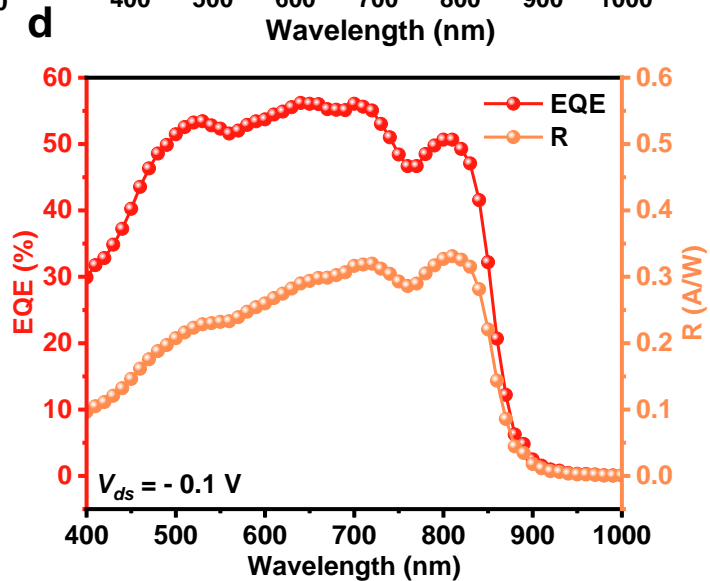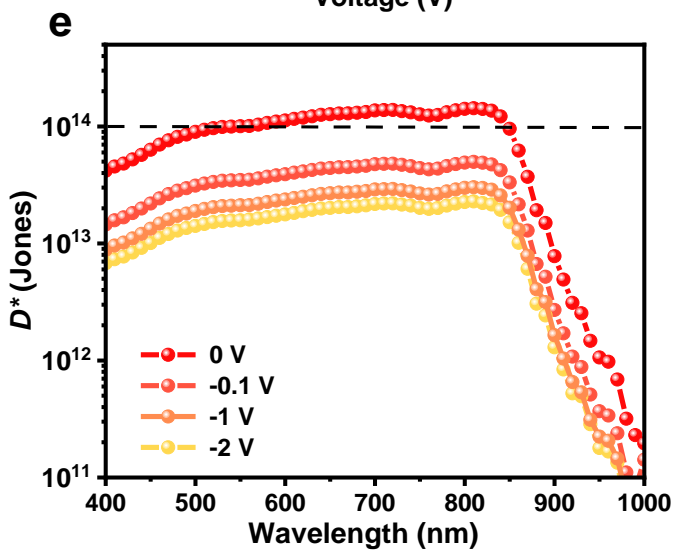

Supplement: Supplementary 1 — Texts S1 to S11 Tables S1 to S5 Figs. S1 to S42 References [56–105] [file research.0939.f1.zip › Figure S42.pdf]

**a**

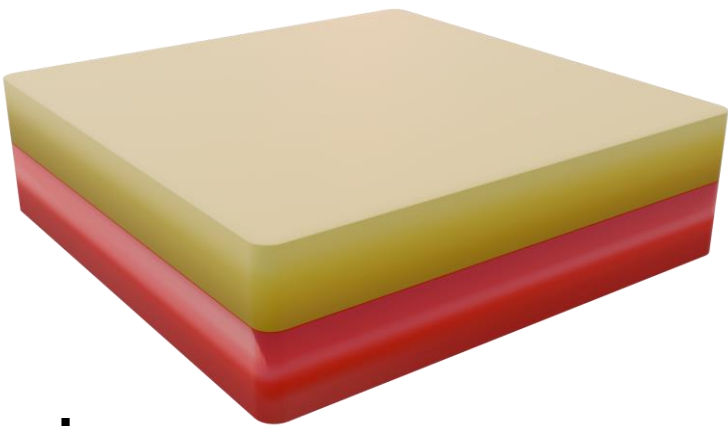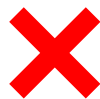

**Low responsivity**

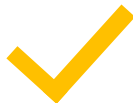

**Low dark current**

**b**

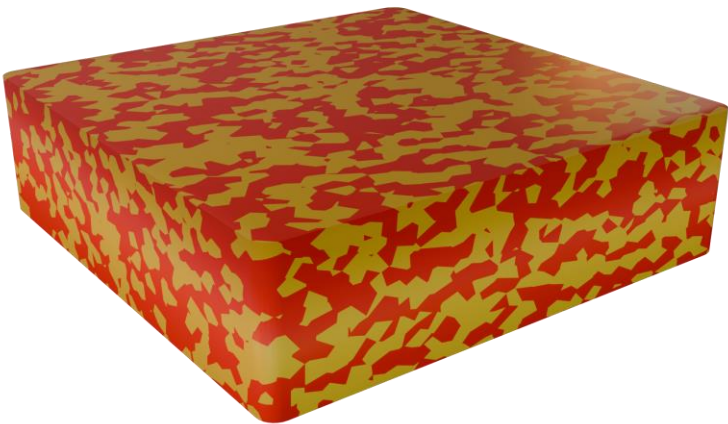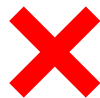

**High responsivity**

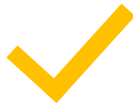

**High dark current**

Supplement: Supplementary 1 — Texts S1 to S11 Tables S1 to S5 Figs. S1 to S42 References [56–105] [file research.0939.f1.zip › Figure S5.pdf]

**a**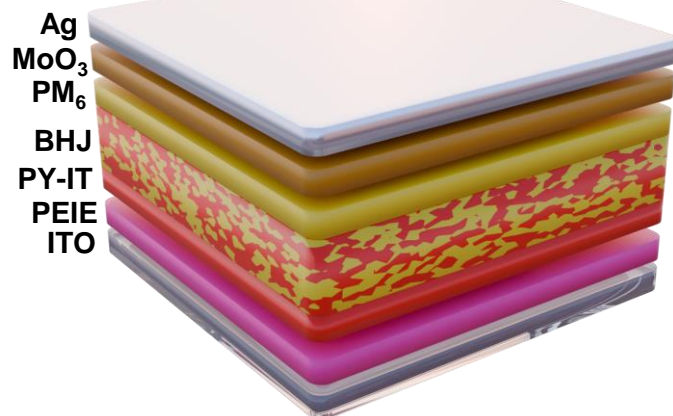**b**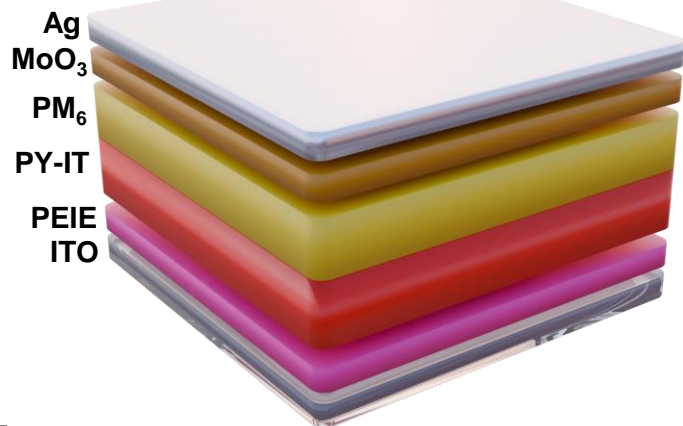**c**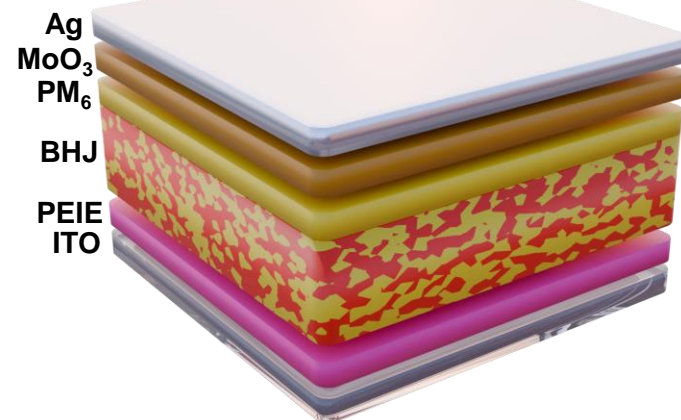**d**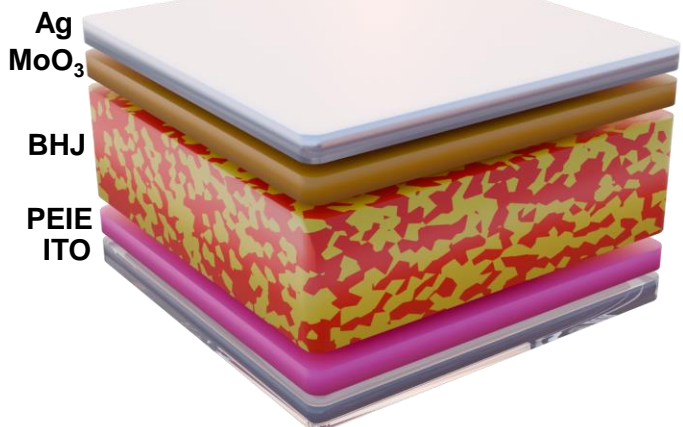**e**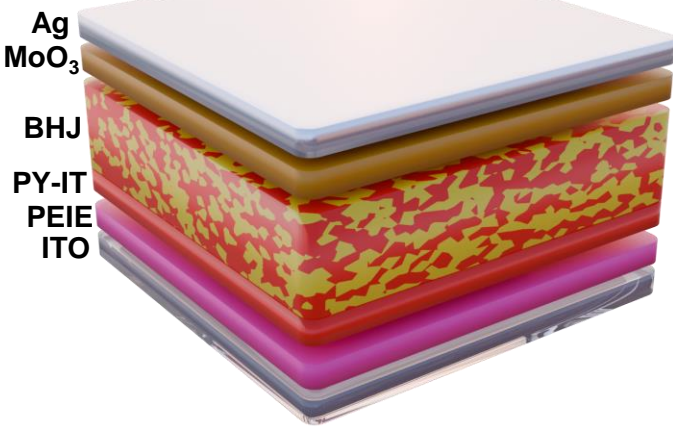

Supplement: Supplementary 1 — Texts S1 to S11 Tables S1 to S5 Figs. S1 to S42 References [56–105] [file research.0939.f1.zip › Figure S6.pdf]

**H<sub>2</sub>O****CH<sub>2</sub>I<sub>2</sub>****PEIE****a**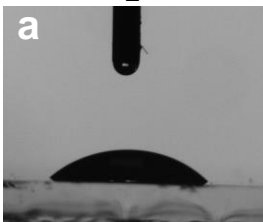**f**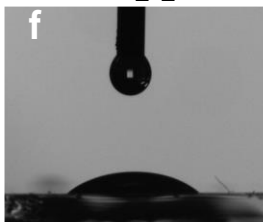**PY-IT****b**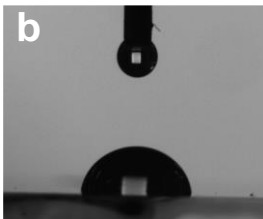**g**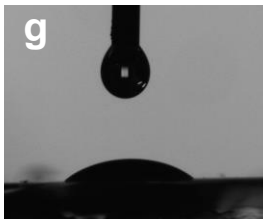**PY-  
IT:PM<sub>6</sub>****c**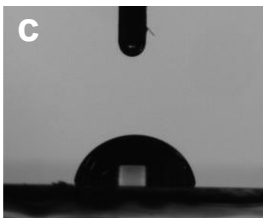**h**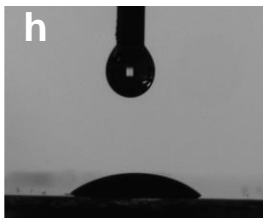**PM<sub>6</sub>****d**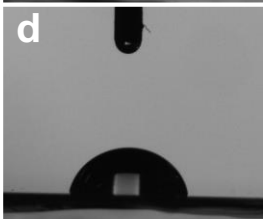**i**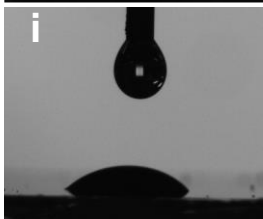**MoO<sub>3</sub>****(e)**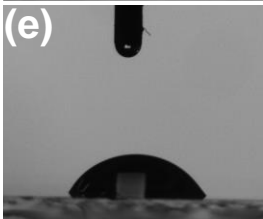**(j)**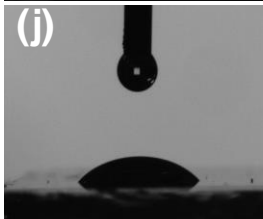

Supplement: Supplementary 1 — Texts S1 to S11 Tables S1 to S5 Figs. S1 to S42 References [56–105] [file research.0939.f1.zip › Figure S7.pdf]

**a**

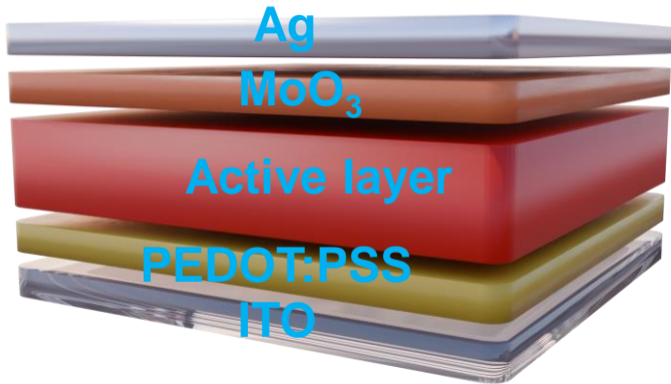

**b**

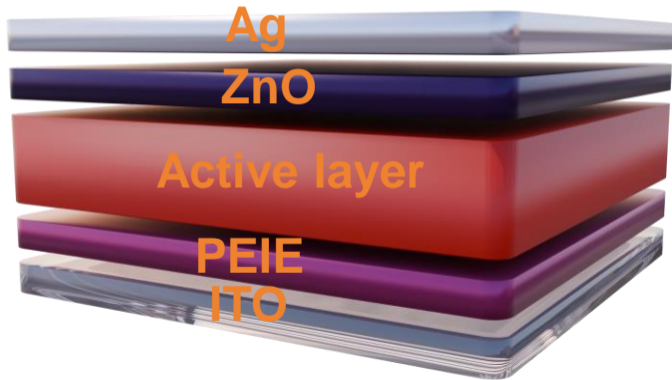

Supplement: Supplementary 1 — Texts S1 to S11 Tables S1 to S5 Figs. S1 to S42 References [56–105] [file research.0939.f1.zip › Figure S8.pdf]

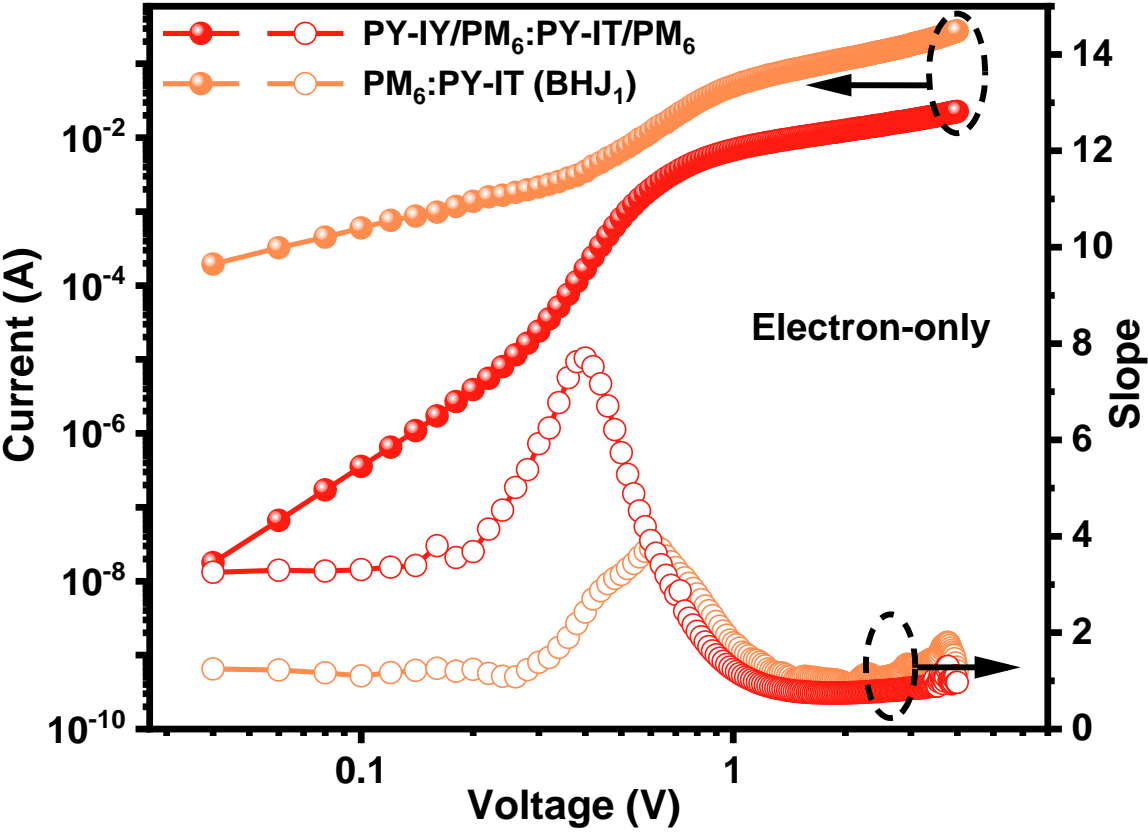

Supplement: Supplementary 1 — Texts S1 to S11 Tables S1 to S5 Figs. S1 to S42 References [56–105] [file research.0939.f1.zip › Figure S9.pdf]
